# Supplementary material for: Enhancing nutritional value and quality of cookies through pumpkin peel and seed powder fortification
Source: PLoS One. 2025 Feb 27;20(2):e0307506. doi: 10.1371/journal.pone.0307506 (PMC11867389; doi:10.1371/journal.pone.0307506)
Supplement: Attachment — Submitted filename: PONE-D-23-44103_MSA.pdf [file pone.0307506.s002.pdf]

PLOS ONE

Enhancing Nutritional Value and Quality of Cookies through Pumpkin Peel and Seed Powder Fortification: A Wheat Flour Replacement Approach

--Manuscript Draft--

**Manuscript Number:**	PONE-D-23-44103
**Article Type:**	Research Article
**Full Title:**	Enhancing Nutritional Value and Quality of Cookies through Pumpkin Peel and Seed Powder Fortification: A Wheat Flour Replacement Approach
**Short Title:**	Enhancing Nutritional Value and Quality of Cookies through Pumpkin Peel and Seed Powder Fortification
**Corresponding Author:**	Md. Asaduzzaman, M. Engg.<br>State University of Bangladesh<br>Dhaka, BANGLADESH
**Keywords:**	Pumpkin, Seeds, Peels, Cookies, Noodles, Nutritional Properties.
**Abstract:**	Background: The study was conducted to develop cookies and noodles using different pumpkin by-products (such as peels and seeds) powders in different concentrations and to evaluate the changes in quality characteristics. The powders were prepared from pumpkin seeds and peels.<br>Result: There were significant differences ($p<0.05$) in protein, fiber, beta-carotene, antioxidants such as total phenolic content (TPC), 2,2- diphenyl-1-picrylhydrazyl (DPPH scavenging activity), Ferric reducing ability (FRAP Ferric reducing power assay), ash content and hunter color values between powders from pumpkin seeds and peels powders. However, pumpkin seed powder had higher protein content than other powders but pumpkin peel powder had better fiber content than seeds powder. The highest amount of fiber content was found in 10% pumpkin peel cookies.<br>Conclusion: The best quality and overall acceptable product were obtained when samples were 10% pumpkin peel powder, 15% pumpkin seed powder, and, 10% pumpkin peel powder noodles.
**Order of Authors:**	Md. Asaduzzaman, M. Engg.
	Mahbubur Rahman, B. Sc.
	Mst. Sorifa Akter, Ph.D.
**Additional Information:**	
**Question**	**Response**
**Financial Disclosure**<br><br>Enter a financial disclosure statement that describes the sources of funding for the work included in this submission. Review the submission guidelines for detailed requirements. View published research articles from *PLOS ONE* for specific examples.<br><br>This statement is required for submission and **will appear in the published article** if the submission is accepted. Please make sure it is accurate.	Yes

Powered by Editorial Manager® and ProduXion Manager® from Aries Systems Corporation

**Funded studies**<br>Enter a statement with the following details:<br>• Initials of the authors who received each award<br>• Grant numbers awarded to each author<br>• The full name of each funder<br>• URL of each funder website<br>• Did the sponsors or funders play any role in the study design, data collection and analysis, decision to publish, or preparation of the manuscript?<br><br>Did you receive funding for this work?	
Please add funding details.<br>as follow-up to "**Financial Disclosure**<br><br>Enter a financial disclosure statement that describes the sources of funding for the work included in this submission. Review the submission guidelines for detailed requirements. View published research articles from *PLOS ONE* for specific examples.<br><br>This statement is required for submission and **will appear in the published article** if the submission is accepted. Please make sure it is accurate.<br><br>**Funded studies**<br>Enter a statement with the following details:<br>• Initials of the authors who received each award<br>• Grant numbers awarded to each author<br>• The full name of each funder<br>• URL of each funder website<br>• Did the sponsors or funders play any role in the study design, data collection and analysis, decision to publish, or preparation of the manuscript?<br><br>Did you receive funding for this work?"	Special Projects from Ministry of Science and Technology<br>Environmental Science: 382
Please select the country of your main research funder (please select carefully as in some cases this is used in fee calculation).	BANGLADESH - BD

as follow-up to "**Financial Disclosure**<br><br>Enter a financial disclosure statement that describes the sources of funding for the work included in this submission. Review the submission guidelines for detailed requirements. View published research articles from *PLOS ONE* for specific examples.<br><br>This statement is required for submission and **will appear in the published article** if the submission is accepted. Please make sure it is accurate.<br><br>**Funded studies**<br>Enter a statement with the following details:<br>• Initials of the authors who received each award<br>• Grant numbers awarded to each author<br>• The full name of each funder<br>• URL of each funder website<br>• Did the sponsors or funders play any role in the study design, data collection and analysis, decision to publish, or preparation of the manuscript?<br><br>Did you receive funding for this work?"	
**Competing Interests**<br><br>Use the instructions below to enter a competing interest statement for this submission. On behalf of all authors, disclose any competing interests that could be perceived to bias this work—acknowledging all financial support and any other relevant financial or non-financial competing interests.<br><br>This statement is required for submission and **will appear in the published article** if the submission is accepted. Please make sure it is accurate and that any funding sources listed in your Funding Information later in the submission form are also declared in your Financial Disclosure statement.	The authors have declared that no competing interests exist

View published research articles from *PLOS ONE* for specific examples.<br><br>**NO authors have competing interests**<br><br>Enter: *The authors have declared that no competing interests exist.*<br><br>**Authors with competing interests**<br><br>Enter competing interest details beginning with this statement:<br><br>*I have read the journal's policy and the authors of this manuscript have the following competing interests: [insert competing interests here]*<br><br>* typeset	
**Ethics Statement**<br><br>Enter an ethics statement for this submission. This statement is required if the study involved:<br><br>• Human participants<br>• Human specimens or tissue<br>• Vertebrate animals or cephalopods<br>• Vertebrate embryos or tissues<br>• Field research<br><br>Write "N/A" if the submission does not require an ethics statement.<br><br>General guidance is provided below. Consult the submission guidelines for detailed instructions. **Make sure that all information entered here is included in the Methods section of the manuscript.**	N/A

**Format for specific study types**<br><br>**Human Subject Research (involving human participants and/or tissue)**<br>• Give the name of the institutional review board or ethics committee that approved the study<br>• Include the approval number and/or a statement indicating approval of this research<br>• Indicate the form of consent obtained (written/oral) or the reason that consent was not obtained (e.g. the data were analyzed anonymously)<br><br>**Animal Research (involving vertebrate animals, embryos or tissues)**<br>• Provide the name of the Institutional Animal Care and Use Committee (IACUC) or other relevant ethics board that reviewed the study protocol, and indicate whether they approved this research or granted a formal waiver of ethical approval<br>• Include an approval number if one was obtained<br>• If the study involved *non-human primates*, add *additional details* about animal welfare and steps taken to ameliorate suffering<br>• If anesthesia, euthanasia, or any kind of animal sacrifice is part of the study, include briefly which substances and/or methods were applied<br><br>**Field Research**<br><br>Include the following details if this study involves the collection of plant, animal, or other materials from a natural setting:<br>• Field permit number<br>• Name of the institution or relevant body that granted permission	
**Data Availability**<br><br>Authors are required to make all data underlying the findings described fully available, without restriction, and from the time of publication. PLOS allows rare exceptions to address legal and ethical concerns. See the PLOS Data Policy and FAQ for detailed information.	Yes - all data are fully available without restriction

A Data Availability Statement describing where the data can be found is required at submission. Your answers to this question constitute the Data Availability Statement and **will be published in the article**, if accepted.<br><br>**Important:** Stating 'data available on request from the author' is not sufficient. If your data are only available upon request, select 'No' for the first question and explain your exceptional situation in the text box.<br><br>Do the authors confirm that all data underlying the findings described in their manuscript are fully available without restriction?	
**Describe where the data may be found in full sentences. If you are copying our sample text, replace any instances of XXX with the appropriate details.**<br><br>• If the data are **held or will be held in a public repository**, include URLs, accession numbers or DOIs. If this information will only be available after acceptance, indicate this by ticking the box below. For example: *All XXX files are available from the XXX database (accession number(s) XXX, XXX.).*<br>• If the data are all contained **within the manuscript and/or Supporting Information files**, enter the following: *All relevant data are within the manuscript and its Supporting Information files.*<br>• If neither of these applies but you are able to provide **details of access elsewhere**, with or without limitations, please do so. For example:<br><br>*Data cannot be shared publicly because of [XXX]. Data are available from the XXX Institutional Data Access / Ethics Committee (contact via XXX) for researchers who meet the criteria for access to confidential data.*<br><br>*The data underlying the results presented in the study are available from (include the name of the third party*	All relevant data are within the manuscript and its Supporting Information files

and contact information or URL).<br>• This text is appropriate if the data are owned by a third party and authors do not have permission to share the data.<br><br>* typeset	
Additional data availability information:	

Manuscript Click here to access/download;Manuscript;Manuscript - PLOS One.docx

Title: "Enhancing Nutritional Value and Quality of Cookies through Pumpkin Peel and Seed Powder Fortification: A Wheat Flour Replacement Approach"

Author List:

1) Md. Asaduzzaman

   Assistant Professor,

   Department of Food Processing and Preservation, Hajee Mohammad Danesh Science and Technology University, Dinajpur, Bangladesh

   Department of Food Engineering and Technology, State University of Bangladesh

   Email: bitatipu@gmail.com; mzaman@sub.edu.bd

2) Mahbubur Rahman

   Department of Food Engineering and Technology, State University of Bangladesh

   Email: rmahbub896@gmail.com

3) Mst. Sorifa Akter*

   Assistant Professor,

   Department of nutrition and food Engineering, Daffodil International University

   Email: sorifa.nfe@diu.edu.bd

* Corresponding Author

Corresponding Author email address: sorifa.nfe@diu.edu.bd

Abstract

Background: The study was conducted to develop cookies and noodles using different pumpkin by-products (such as peels and seeds) powders in different concentrations and to evaluate the changes in quality characteristics. The powders were prepared from pumpkin seeds and peels.

Result: There were significant differences ($p<0.05$) in protein, fiber, beta-carotene, antioxidants such as total phenolic content (TPC), 2,2- diphenyl-1-picrylhydrazyl (DPPH scavenging activity), Ferric reducing ability (FRAP Ferric reducing power assay), ash content and hunter color values between powders from pumpkin seeds and peels powders. However, pumpkin seed powder had higher protein content than other powders but pumpkin peel powder had better fiber content than seeds powder. The highest amount of fiber content was found in 10% pumpkin peel cookies.

Conclusion: The best quality and overall acceptable product were obtained when samples were 10% pumpkin peel powder, 15% pumpkin seed powder, and, 10% pumpkin peel powder noodles.

Keywords: Pumpkin, Seeds, Peels, Cookies, Noodles, Nutritional Properties.

1. INTRODUCTION

Pumpkin (*Cucurbita moschata*) is a fruit vegetable, not only grown in the Western Hemisphere but also in Tropical Asia in countries such as Bangladesh, India, Indonesia, Malaysia and Philippines [1]. The total world agricultural production has increased annually over the past 10 years from 50 million tons in 1996 to 203 million tons in 2005 while in Bangladesh pumpkin cultivation has an annual production of 340 thousand metric tons [1, 2].

Pumpkins have a large range of uses as a potentially valuable food source for both humans and animals. The edible flesh (pulp) of the pumpkin fruit may be eaten as a vegetable or incorporated into food products such as curry, pies, bread, soup, or jams. The food industry utilizes pumpkin powders and flakes as thickeners, flavoring and coloring agents in soups and sauces [1, 3]. However, food processing techniques create large quantities of waste in the form of peels (skin) and seeds. These waste streams can be utilized as an added value ingredient with the potential of being processed into food products in a similar way to other waste stream products and fiber-rich components [4-6].

Pumpkin peels and seeds are rich in vitamins, proteins, minerals and antioxidants (β-carotene and tocopherols) [7, 8]. In addition to nutrient composition, it is composed of various biological active components such as polysaccharides, protein, peptide, sterols and para-amino benzoic acids [7, 9]. These biological active components have shown to be wide range of medicinal properties such as anti-diabetic activity, antioxidant activity, anti-carcinogenic effect and anti-microbial effect [7, 10].

With these beneficial effects of pumpkin peels and seeds, it can be incorporated into different value-added products such as cookies and snacks (noodles). In recent times, fruits and vegetables by-products has attracted more attention due to their health benefits. Therefore, it has been focused on under-utilized indigenous crops waste, for example the pumpkin peels and seeds are useful in food industries for the formulation of value-added products. Although there have been studies with cookies and noodles formulations with pumpkin peels and seeds. Therefore, the objective of this study was to utilize pumpkin by-products and develop pumpkin peels and seeds powders mixed cookies and noodles and evaluation of its physical, chemical and sensory properties.

2. MATERIALS AND METHODS

2.1 **Raw Materials, chemicals and other ingredients**

Ripe pumpkins were sourced from a local market in Dhaka, chosen for uniformity, color, and lack of defects. Pumpkin seeds, wheat flour, oil, sugar, skimmed milk powder, butter, eggs, baking powder, salt, and margarine were obtained locally. Citric acid and packaging materials came from a local company. All items, except butter and eggs stored in a refrigerator, were kept at room temperature. High-density polyethylene bags stored peel powder, seed powder, cookies, and noodle samples.

2.2 Preparation of pumpkin peels and seeds powder

Ripe pumpkin was washed, sliced, and its peels collected. The peels were treated with 0.5% citric acid, dried at 65°C for 8 hours. Purchased seeds were oven-dried at 80°C for 22 hours. Both dried peels and seeds were blended, sieved through a 50-mesh size, and packed in high-density polyethylene bags for analysis.

2.3 Preparation of cookies and noodles from pumpkin seeds and peels

2.3.1 Powders Formulation of Cookies

There were two types of cookies were formulated. Cookies were prepared from pumpkin seeds and peels powders. Pumpkin seeds powders and peels powders were substituted at 5%, 10%, 15%, 20% and 5%, 10%, 20%, respectively with marketed wheat flour which were represented in the figure 01 and 02. Cookies prepared from market wheat flour serve as control.

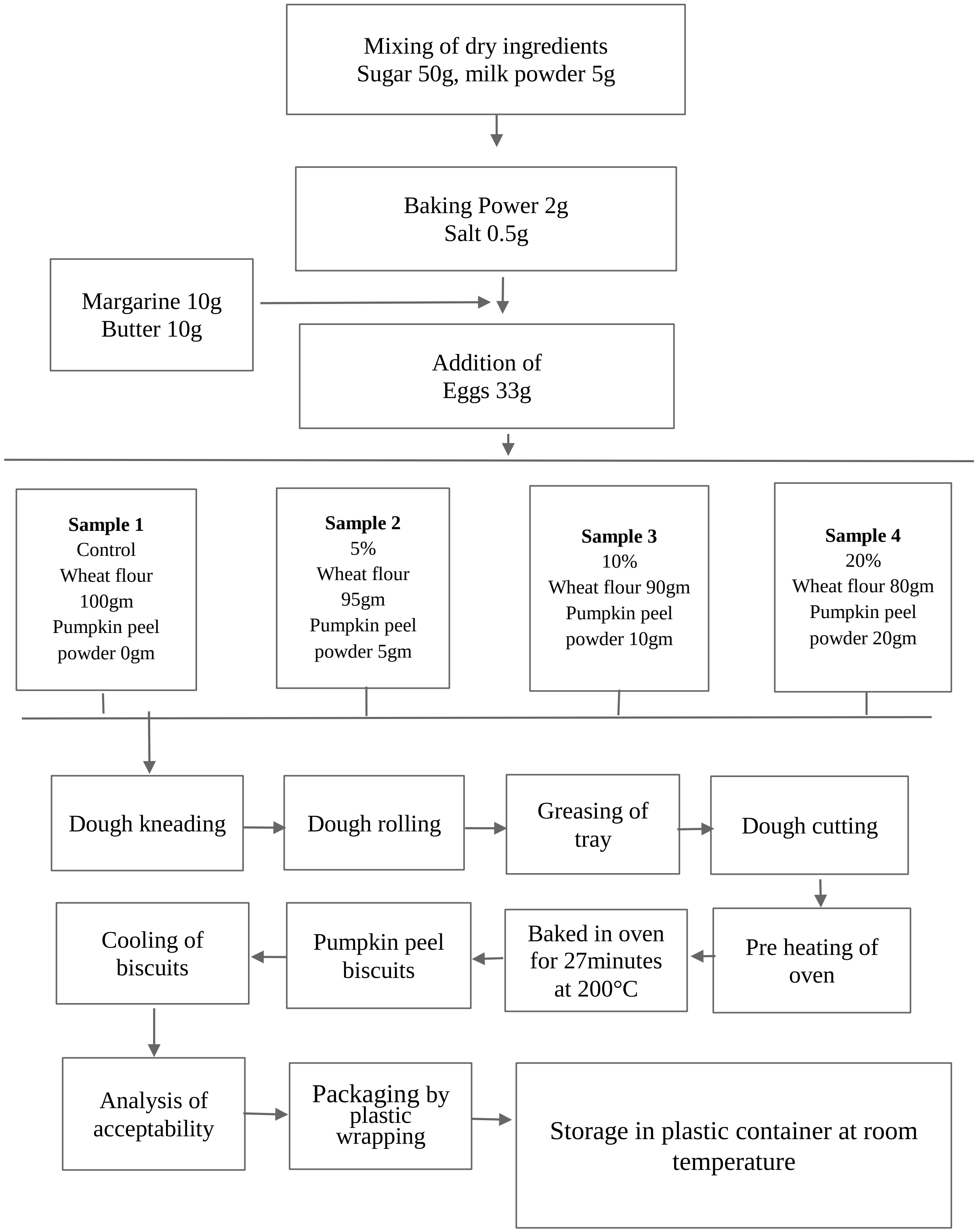

Figure 01: Flow diagram of preparation of pumpkin peel cookies samples

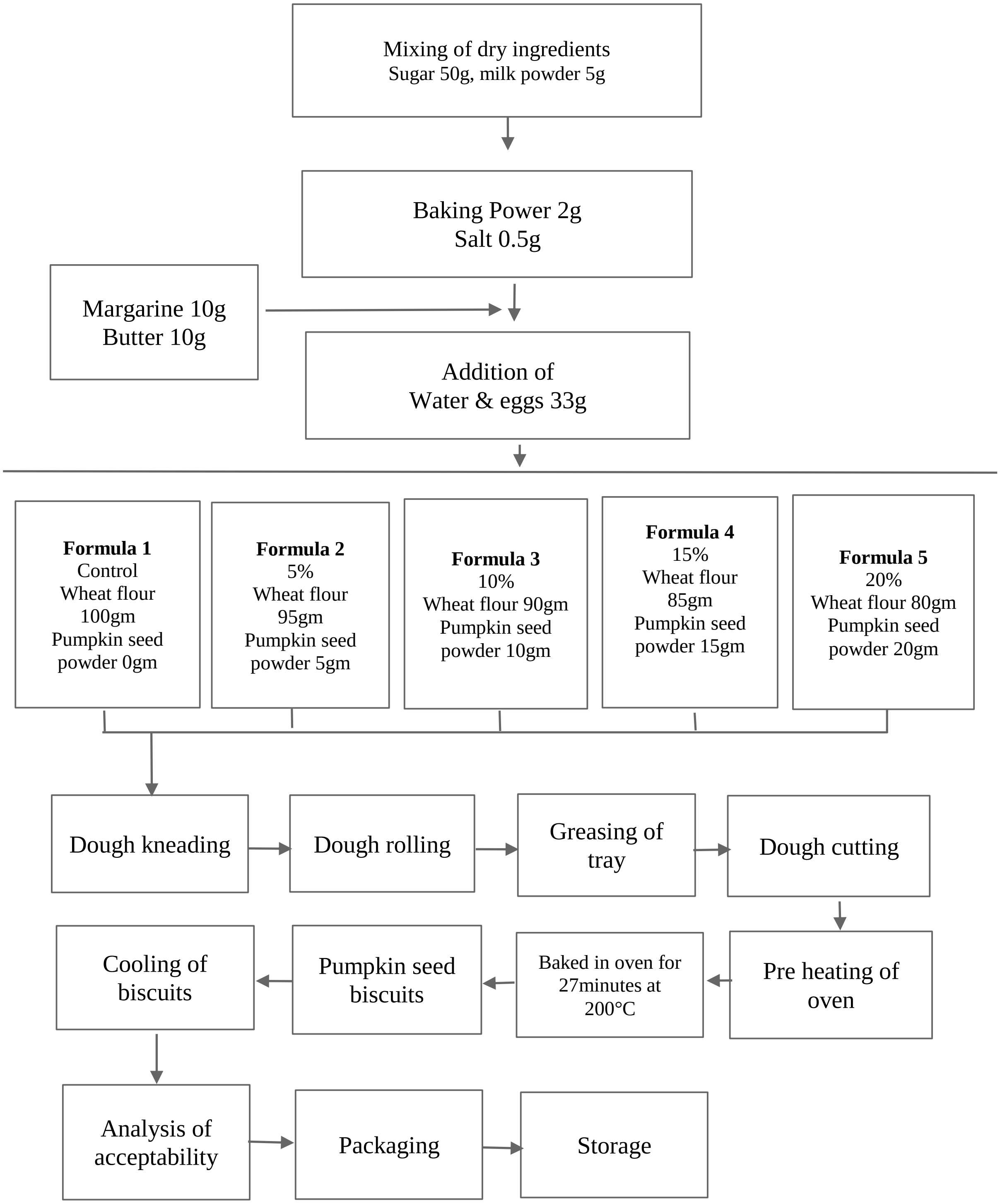

Flow diagram 02: Preparation of pumpkin seed cookies samples

2.3.2 Formulation of Noodles

Noodles were also made from pumpkin seeds powder and pumpkin peels powders. Three types of noodles were formulated such as wheat four fortified with 10% seed powder, 10% peel powder and 10% seed powder mixed with 10% peel powder which were represented in the figure 03. Noodles prepared from market wheat flour serve as control.

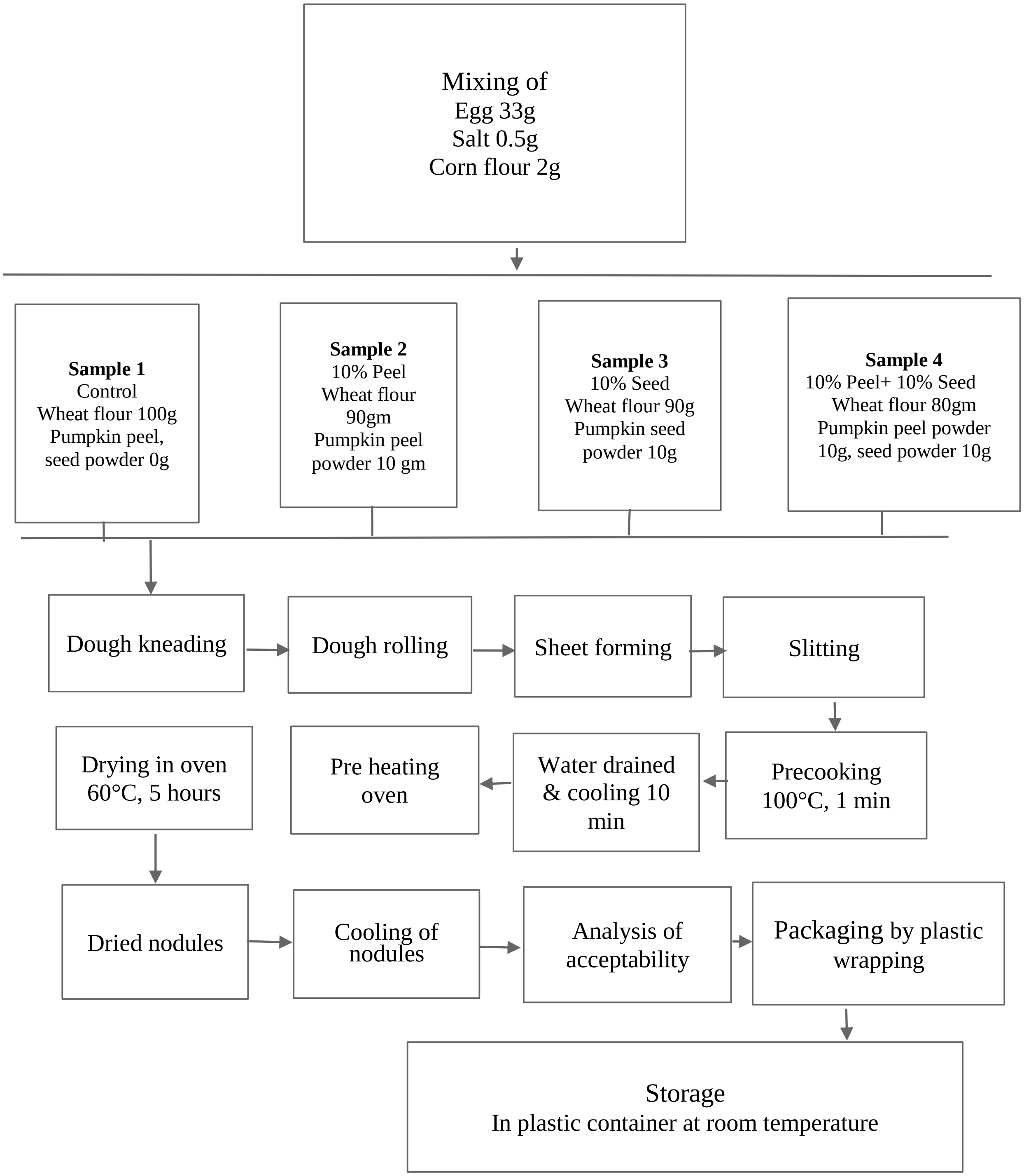

Figure 03: Flow diagram of Preparation of noodles samples

2.4 Proximate composition

Moisture, protein, fat and ash content of sweet potato peels powders and baked cakes were determined by official methods of AOAC 1998 [11]. Carbohydrate was expressed as the difference from moisture, protein, fat, and ash. The caloric value was calculated to conversion factors of 4 kcal g-1 for proteins, 9 kcal g-1 for fats and 4 kcal g-1 for carbohydrates [12]. All the analyses were done in triplicates.

2.5 Determination of crude fiber

Acid and alkali hydrolysis followed by ignition of the residue as described by the method AOAC (1984) was used to estimate the fiber content in sweet potato peels powders and cakes samples [11]. Then the fiber content was determined by following equation

$$\%\ of\ Fibre = \frac{Initial\ weight - Final\ Weight}{Sample\ weight} \times 100$$

2.6 Determination of total polyphenol content

Determination of the total polyphenol content was done by Sorifa et al., (2010) method with some modifications [13]. Total phenols were calculated based on standard curves of gallic acid and expressed as mg/1 g utilizing spectrophotometer (T80 U/VIS, United Kingdom).

2.7 Determination of DPPH:

The scavenging effects of samples for 1,1-diphenyl-2-picrylhydrazyl (DPPH) radical were monitored according to the modified method of the previous report by Sorifa et al., 2010 [13].

2.8 Determination of FRAP

The chelating capacity of PSE was determined with a method described by Sorifa et al., 2010 [13].

2.9 Cooking properties

The cooking properties of noodles were evaluated by a method similar to that described by Mestres et al., 1988 [14]. These evaluations of cooking properties were all done in duplicate.

2.10 Sensory evaluation

Cookies underwent sensory analysis using a 9-point Hedonic scale by 10 panelists from the Department of Food Engineering and Technology, State University of Bangladesh. Attributes such as color, flavor, taste, texture, and overall acceptability were assessed. Statistical analysis, including variance and Duncan's Multiple Range Test (DMRT), was performed using the Statistical Analysis System (SAS, 1985) procedures.

2.11 Statistical analysis

The results were expressed as the mean, standard error of the mean (SEM), and coefficient of variation of each species for each parameter was determined. Data were statistically analyzed (R statistical software 3.4.1) by one-way analysis of variance (ANOVA). Mean comparisons were performed using Duncan's multiple range tests for significant effect at $P < 0.05$.

3. RESULT AND DISCUSSION

3.1 Proximate analysis

3.1.1 Proximate analysis of wheat flour, pumpkin seeds and peels powder

The three kinds of powders were analyzed for proximate analyses which are wheat flour which collected from local market, pumpkin seed powder and pumpkin peel powder. The results showed in the table 01 where results showed significantly higher amounts of moisture, ash, protein, fat, fiber and beta-carotene contents in pumpkin seeds powder and peels powder as compared to marketed wheat flour (control). Similar results were observed for pumpkin seeds powder and peels powder by Ashik et al., 2022; who reported that moisture, ash, protein, fat and fiber content of pumpkin seeds powder was 2.75%, 5.20%, 34.19%, 44.01% and 4.12 %, respectively whereas pumpkin peels powder was 10.31%, 6.04%, 12.30 %, 5.77 % and 13.91%, respectively [15]. Sometimes the difference in protein and fat value in pumpkin seeds and peels were observed. This might be due to the variation in environment during harvesting such as application of fertilizer (nitrogen content), growing conditions and time and also location of growing areas, oxidation of the fat because most of the fat content in seeds and peels is unsaturated and undergo oxidation easily by atmospheric oxygen.

Table 01: Proximate compositions of powder samples

Powder Samples	Moisture Content (%)	Ash Content (%)	Protein Content (%)	Fat Content (%)	Fiber Content (%)	Beta- carotene (μg/g)
Wheat flour	10.60±0.87[a]	0.57±0.02[b]	9.62 ±0.01[b]	1.08±0.03[a]	0.51±0.09[b]	14.56±0.34[b]
Pumpkin seed powder	3.21±0.16[a]	5.18±0.03[a]	33.25±0.01[a]	43.96±0.33[a]	3.9.1±0.48[b]	39.46±0.36[b]
Pumpkin peel powder	10.21±0.13[a]	4.40±0.03[a]	15.09±0.30[ab]	2.94±0.01[a]	16.83±1.74[a]	123.55±0.33[a]

All values are express as mean ± SD.
Mean followed by different superscript letters in each column are significantly different ($p<0.05$)

3.1.2 Proximate analysis of pumpkin seed cookies

The proximate compositions of cookies from pumpkin seeds was analyzed for moisture, ash, protein, fat, fiber and beta-carotene which presented in Table 02. Results demonstrated that all pumpkin seed cookies had higher amount of protein, fat, fiber and beta-carotene content as compared to control (100% wheat flour cookie).

Protein content of different cookies varied from 8.09 to 12.03/100 g that was similar to found by

Syam et al. (2019) who reported protein value in pumpkin seed cookie 11.20/100g and also by Dhiman et al. (2018) who reported protein value in cookies as 8.47 to 12.56/100 g [16]. Sample E had highest protein value (12.03/100g) compared with other recipes. On the contrary, sample A which is backed by market wheat flour had the lowest value of protein (8.09/100 g) than other samples. The variations in protein value increase because pumpkin seed contain more protein than market wheat flour.

Fat values were appreciably different among cookies and highest was found in sample D (13.95/100g), where lowest content (11.62/100g) were studied in sample A. This observation was comparable to that found by Syam et al. (2019) who reported fat value in pumpkin seed cookies33.05/100g [17].The variation in fat value in different cookies may be due to oxidation of the fat because most of the fat content in cookies is unsaturated and undergo oxidation easily by atmospheric oxygen. The ash content of different cookies varied between 0.91/100 to 1.36/100g. This result was consistent to be found by Syam et al. (2019) who reported ash content in cookies as 1.65/100g [17]. Lowest ash value was found in sample A which is backed from market wheat flour (0.91/100 g) variety while highest value (1.36/100 g) was studied in sample E which backed from 85% market wheat flour /15%pumpkin seed cookie.

Fiber values were appreciably different among cookies and highest was found in sample D (1.61/100g), where lowest content (0.32/100g) were studied in sample A. This observation was comparable to that found by Syam et al. (2019) who reported fat value in pumpkin seed cookies 1.64/100g [17]. Low molecular weight components such as minerals, vitamins and sugars might be lost during washing treatment, leading to a relative increase in the dietary fibre content (Wenberg et al., 2006). The Beta-carotene content of different cookies varied between 4.07 ug/g to 6.36 ug/g. This result was consistent to be found by Dhiman et al. (2018) who reported Beta-carotene content in cookies as 3.22 to 3.78 mg/100g [16]. Lowest Beta-carotene content was found in sample A which is backed from market wheat flour (4.07 ug/g) variety while highest value (6.36 ug/g) was studied in sample E which backed from 80% market wheat flour 20%pumpkin seed cookie.

Table 02: Proximate compositions of pumpkin seeds cookies

Cookie Samples	Moisture content (%)	Ash content (%)	Protein content (%)	Fat content (%)	Fiber content (%)	Beta-carotene (ug/g)
A	6.68 ±0.19^{bc}	0.91±0.01^{c}	8.09 ±1.54^{c}	11.62±0.56^{d}	0.32±0.15^{c}	4.07±0.36^{c}
B	5.91±0.31^{c}	1.02±0.03^{c}	9.84±0.31^{b}	12.03±0.02^{cd}	0.62±0.19^{bc}	5.18±0.34^{b}
C	7.79±0.56^{ab}	1.24±0.01^{ab}	9.84 ±0.31^{b}	12.89±0.01^{bc}	0.92±0.06^{b}	5.27±0.39^{ab}
D	7.96± 0.06^{a}	1.15±0.02^{b}	11.15±0.31^{a}	13.95±0.70^{a}	1.61±0.31^{a}	5.77±0.74^{ab}
E	7.74±0.54^{ab}	1.36±0.03^{a}	12.03±0.31^{a}	13.78±0.86^{ab}	1.52±0.30^{a}	6.36±1.08^{a}

Values are means of triplicate analysis ± Standard deviation. A=Control (100% market wheat flour, Cookie), B=95% market wheat flour /5% pumpkin seed cookie, C=90% market wheat flour /10%pumpkin seed cookie, D=85% market wheat flour /15%pumpkin seed cookie, E=80% market wheat flour /20%pumpkin seed cookie)
All values are express as mean ± SD
Mean followed by different superscript letters in each column are significantly different ($p<0.05$)

3.1.3 Proximate compositions of pumpkin peel cookies

The composition of cookies from pumpkin peels was analyzed for moisture, ash, protein, fat, fiber and beta-carotene. The results presented in table 03 where sample D contained the highest amount of moisture and sample A had the lowest level of moisture. These values were higher to those reported by Mishra and Sharma (2019) who found that moisture content in cookies is 6.59/100g and also by Saleh and Ali (2020) found that moisture content in pumpkin peel cookie was 5.62/100g to 7.09/100g [18, 19].

Protein content of different pumpkin peel cookies varied from 8.09 to 9.04/100 g that was similar to found by Saleh and Ali (2020) who reported protein value in pumpkin peel cookie 7.73/100g to 9.42/100g and higher found by Mishra and Sharma (2019) who reported protein value in cookies as 0.08/100g [18]. Sample D had highest protein value (9.40/100g) compared with other recipes. On the contrary, sample A which is backed by market wheat flour had the lowest value of protein (8.09/100 g) than other samples. The variation in protein value increase because pumpkin peel contains more protein than market wheat flour.

Fat values were appreciably different among cookies and highest was found in sample C (11/100g), where lowest content (10.84/100g) were studied in sample A. This observation was comparable to that found by Mishra and Sharma (2019) who reported fat value in pumpkin peel cookies 21.75/100g and also found by Saleh and Ali (2020) who reported fat value in pumpkin peel cookie 14.82/100g to 15.14/100g [18, 19]. The variation in fat value in different cookies may be due to oxidation of the fat because most of the fat content in cookies is unsaturated and undergo oxidation easily by atmospheric oxygen. The ash content of different cookies varied

between 0.91/100 to1.34/100g. This result was consistent to be found by Saleh and Ali (2020) who reported ash content in cookies as 1.30/100g to 2.62/100g [19]. Lowest ash value was found in sample A which is backed from market wheat flour (0.91/100 g) variety while highest value (1.34/100 g) was studied in sample D which backed from 85% market wheat flour /15%pumpkin peel cookie.

Fiber values were appreciably different among cookies and highest was found in sample C (5.08/100g), where lowest content (0.32/100g) were studied in sample A. This observation was comparable to that found by Saleh and Ali (2020) who reported fiber value in pumpkin peel cookies 1.18/100g to 3.17/100g and higher then found by Mishra and Sharma (2019) who reported fiber value in pumpkin peel cookies 0.16/100g [18, 19].

The Beta-carotene content of different cookies varied between 4.07 ug/g to 29.38 ug/g. This result was higher to be found by Saleh and Ali (2020) who reported Beta-carotene content in cookies as 3.08 to 11.54 mg/100g [19]. Lowest Beta-carotene content was found in sample A which is backed from market wheat flour (4.07 ug/g) variety while highest value (39.38 ug/g) was studied in sample D which backed from 80% market wheat flour /20%pumpkin peel cookie. The variation in beta- carotene value increase because pumpkin peel contain more beta-carotene then market wheat flour.

Table 03: Proximate compositions of pumpkin peel cookies

Cookie Samples	Moisture Content (%)	Ash Content (%)	Protein Content (%)	Fat Content (%)	Fiber Content (%)	Beta-carotene (ug/g)
A	6.68 ±0.19^{c}	0.91±0.01^{c}	8.09 ±1.54^{a}	11.62±0.56^{a}	0.32±0.15^{c}	4.07±0.36^{c}
B	7.17±0.17^{bc}	1.04±0.01^{b}	8.75±0.61^{a}	10.84±0.16^{b}	2.07±0.19^{bc}	12.40±0.36^{b}
C	7.63±0.52^{b}	1.10±0.02^{b}	8.96 ±0.31^{a}	11.00±0.21^{ab}	5.08±0.06^{a}	25.52±0.19^{a}
D	9.02± 0.43^{a}	1.34±0.01^{a}	9.40±0.30^{a}	10.95±0.13^{ab}	3.08±0.31^{ab}	29.38±0.37^{a}

Values are means of triplicate analysis ± Standard deviation. A=Control (100% wheat, Cookie), B=95%wheat/5%pumpkin peel cookie, C=90%wheat/10%pumpkin peel cookie, D=80%wheat/20%pumpkin peel cookie)
All values are express as mean ± SD.
Mean followed by different superscript letters in each column are significantly different ($p<0.05$)

3.1.4 Proximate compositions of noodles samples:

The composition of noodles from pumpkin seeds and peels powder was analyzed for moisture content, protein, ash and fat content. The results obtained were presented in Table 04. Sample had lower moisture content as compared to all other peels and seeds fortified noodles. Although these values had no significant differences. The protein content of noodles sample ranged from 4.81 to 5.47 %. There were no significant differences in protein content among all noodles sample. All

noodles' samples had fat content ranged from 0.59 to 4.96 %. Sample D (80%wheat/10%pumpkin seeds and 10% pumpkin peels noodles) had highest fat content which is slightly higher than control. The ash content of noodles sample ranged from 1.60 to 2.2 7%. Sample D (80%wheat/10%pumpkin seeds and 10% pumpkin peels noodles) sample had highest ash content. There was significantly different in ash content was observed for sample D. This might be due to the higher amount of mineral content present in the seeds and peels samples. The fiber content of noodles sample ranged from 1.49 to 8.88%. Control (wheat flour noodles) sample had lower fiber content as compared to all other peels and seeds fortified noodles.

Table 04: Proximate compositions of noodles samples.

Noodles Samples	Moisture Content (%)	Ash Content (%)	Protein Content (%)	Fat Content (%)	Fiber Content (%)	Beta-carotene (ug/g)
A	8.94±0.62^{a}	1.60±0.026^{b}	5.47 ±0.31^{a}	4.30±0.47^{a}	1.49 ±0.23^{a}	0.324 ±0.003^{a}
B	9.67±0.97^{a}	1.85±0.20^{b}	5.03±0.30^{a}	0.59 ±0.01^{a}	6.36 ±1.58^{a}	0.315 ±0.004^{a}
C	9.44±0.85^{a}	1.72±0.034^{b}	4.81±0.02^{a}	4.96 ±0.44^{a}	8.88±1.13^{a}	0.222±0.004^{b}
D	9.23±0.42^{a}	2.27±0.140^{a}	5.25 ±0.03^{a}	0.59± 0.07^{a}	8.82 ± 0.19^{a}	0.318 ± 0.002^{a}

Values are means of triplicate analysis ± Standard deviation. A=Control (100% wheat flour noodles), B=90%wheat/10% pumpkin seeds noodles, C=90%wheat/10% pumpkin peels noodles, D=80%wheat/10%pumpkin seeds and 10% pumpkin peels noodles
All values are express as mean ± SD.
Mean followed by different superscript letters in each column are significantly different ($p<0.05$)

3.2 β-Carotene determination for noodles

Almeida et al. (2011) reported that, foods which are rich in antioxidants play an essential role in the prevention of carcinogenic diseases. The antioxidant capacities of leaves may vary depending on their contents of vitamin C, vitamin E, carotenoids, and particularly β-carotene [20], and lycopene [21] as well as other pigments such as flavonoids and other polyphenols [22]. The results for β-carotene content of the noodles are listed on Table 04. Carotenoids are tetraterpenoids found throughout not only the flowering plant kingdom but also in vegetables and fruits as a pigment mostly responsible for various color such as red, orange or yellow color in peels, seeds, flash leaves and fruits which are important as vitamin A precursors. As they are found widely in all kinds' plants and plant parts, it is not surprising that a large number of carotenoids have been reported in the pumpkin seeds and peels powder [23]. Noodles from pumpkin seeds powder showed the highest ($P < 0.05$) content of β-carotene except control. Noodles manufactured from peels powder and mixture of peels and seeds have also shown to be an excellent source of β-carotene. Similar levels of β-carotene have been previously reported for pumpkin flesh powder [24]. However, these pumpkin peels and seeds powder should not be

considered as a rich source of carotenoids, where values as high as 161 mg/100 g d.b. for wine palm (*Mauritia vinifera*) one of the most important vitamin A precursors has been represented [25, 26].

3.3 Cooking properties (Cooking yield)

Generally speaking, high cooking yield gains and low cooking loss would be desirable characteristics for high quality of any kinds of noodles. Flour noodles with added different types of pumpkin powder such as peels powder, seeds powder and mixture of peels and seeds powders had lower cooking yield gains than the flour noodle samples with no added pumpkin (Table 05).

It was found that control sample contained the highest amount of cooking yield and 10% seeds powder noodles sample had the lowest level of cooking yield while 10% pumpkin seeds powder has lowest cooking loss and the mix powder has highest cooking loss. Including controls all noodles' samples cooking loss is considerable higher then Mestres et al. (1988) [14]. But all noodles' samples cooking yield is much higher [24].

The parameters of cooked noodles varied considerably for gumminess and hardness. However, springiness, cohesiveness, and chewiness were not different among the samples, indicating the pumpkin powder did not affect such parameters [24, 27].

Table 05: Cooking properties of noodles

Sample	Noodles cooking Yield	Noodles cooking Loss
Control	275.254 ± 0.10^{a}	3.723 ± 0.010^{b}
10% seeds powder	256.368 ± 0.10^{b}	3.421 ± 0.010^{b}
10% Peels powder	250.885 ± 0.10^{b}	4.217 ± 0.010^{ab}
10% seeds + 10% Peels powder	260.089 ± 0.01^{b}	5.007 ± 0.002^{a}

All values are express as mean ± SD.
Mean followed by different superscript letters in each column are significantly different ($p<0.05$)

3.4 Hunter Color Values

Hunter color values were represented by L*, a*, b*, and ΔE where L* is a measure of lightness, a* redness, b* yellowness and ΔE color differences. The Hunter color parameters L*, a*, b*, and ΔE have been widely used to evaluate color changes during dehydration of fruits and vegetable products. There are three types of powdered samples such as wheat flour which collected from local market served as control, Pumpkin seed powder and Pumpkin peel powder. The results of Hunter Color Values (L*, a*, b*, ΔE) of pumpkin seeds and peels powder are represented in table 06, pumpkin seed cookies are shown in Table 7 & pumpkin peels cookies are shown in Table 8.

Results indicate slightly decrease in L*, b* and ΔE value while increase in a* value of all cookies samples prepared from both seeds and peels powders with the level of increasing sample concentrations. The increase of hunter a* values (redness) with increase of sample concentration

for all cookie samples might be due to the influence of beta-carotene concentration. The flour colors can best be described by the change in total color difference, ΔE values. The lower ΔE values may be due to the loss of phenolics and oxidation during drying, boiling treatment and oven temperature [28]. There were no significant differences in ΔE values among all cookie's samples.

Table 06: Hunter Color Values (L*, a*, b*, ΔE) of pumpkin seeds and peels powder samples

Powder Samples	L*	a*	b*	ΔE
Wheat Flour	80.53 ± 2.87^{a}	1.82 ± 0.19^{a}	10.57 ± 0.55^{b}	71.98 ± 13.59^{a}
Pumpkin seed powder	52.79 ± 1.24^{b}	3.57 ± 0.74^{a}	19.46 ± 1.35^{a}	12.62 ± 2.58^{a}
Pumpkin peel powder	65.09 ± 4.74^{ab}	1.34 ± 0.26^{a}	18.72 ± 4.27^{a}	17.51 ± 2.38^{a}

All values are express as mean ± SD.
Mean followed by different superscript letters in each column are significantly different ($p<0.05$).

Table 07: Hunter Color Values (L*, a*, b*, ΔE) of pumpkin seeds cookies

Cookie Samples	L*	a*	b*	ΔE
A	50.94 ± 1.85^{ab}	16.20 ± 0.80^{a}	28.46 ± 0.45^{a}	60.56 ± 1.98^{ab}
B	49.14 ± 1.90^{ab}	13.51 ± 0.96^{b}	26.88 ± 0.41^{ab}	57.621 ± 2.04^{ab}
C	44.04 ± 2.93^{b}	14.40 ± 1.45^{ab}	23.40 ± 2.43^{b}	51.91 ± 3.98^{b}
D	43.02 ± 0.41^{b}	14.46 ± 0.48^{ab}	24.50 ± 0.82^{ab}	$51.58 \pm .87^{b}$
E	54.07 ± 1.67^{a}	13.95 ± 1.18^{ab}	27.60 ± 0.13^{ab}	62.29 ± 1.77^{a}

Values are means of triplicate analysis ± Standard deviation. A=Control (100% wheat, Cookie), B=95%wheat/5%pumpkin seed cookie, C=90%wheat/10%pumpkin seed cookie, D=85%wheat/15%pumpkin seed cookie, E=80%wheat/20%pumpkin seed cookie)
All values are express as mean ± SD.
Mean followed by different superscript letters in each column are significantly different ($p<0.05$)

Table 08: Hunter Color Values (L*, a*, b*, ΔE) of pumpkin peels cookies

Cookie Samples	L*	a*	b*	ΔE
A	50.94 ± 1.85^{a}	16.20 ± 0.80^{a}	28.46 ± 0.45^{a}	60.56 ± 1.98^{a}
B	55.84 ± 1.60^{a}	10.67 ± 0.19^{b}	28.97 ± 0.24^{a}	63.81 ± 1.54^{a}
C	51.19 ± 1.13^{a}	9.70 ± 0.72^{b}	29.48 ± 0.86^{a}	59.86 ± 1.51^{a}
D	41.50 ± 2.34^{b}	13.25 ± 0.61^{ab}	24.08 ± 2.27^{b}	49.78 ± 3.21^{b}

Values are means of triplicate analysis ± Standard deviation. A=Control (100% wheat, Cookie), B=95%wheat/5%pumpkin peel cookie, C=90%wheat/10%pumpkin peel cookie, D=80%wheat/20%pumpkin peel cookie)
All values are express as mean ± SD.
Mean followed by different superscript letters in each column are significantly different ($p<0.05$)

3.5 Antioxidant Properties

3.5.1 Total Phenolic Compound and Antioxidant Properties (DPPH scavenging activity and FRAP Ferric reducing power assay) of powder samples

The total phenolic compound, DPPH and FRAP antioxidant properties of market wheat flour, pumpkin seed powder and pumpkin peel powder are shown in Table 09. The highest total phenolic compound (268.38 mg/100g) was found in pumpkin seed powder sample. It is commonly known as seeds are an excellent source of nutritional compositions and other bioactive compound specially phenols.

Table 09: Total phenolic compound and antioxidant properties (DPPH and FRAP) of market wheat flour, pumpkin seed powder and pumpkin peel powder sample

Powder Samples	Total Phenolic Compound (mg/100g)	DPPH scavenging activity (mmole/100g)	FRAP Ferric reducing power assay (mmole/100g)
Wheat Flour	83.62±2.85	122.23±6.86	279.75±37.12
Pumpkin seed powder	268.38±1.02	160.17±14.55	688.50±10.60
Pumpkin peel powder	87.82±1.03	217.52±27.76	1374.75±15.90

All values are express as mean ± SD.
Mean followed by different superscript letters in each column are significantly different ($p<0.05$)

3.5.2 Total Phenolic Compound and Antioxidant Properties (DPPH scavenging activity and FRAP Ferric reducing power assay) of pumpkin seeds cookies

Table 10 shows the total phenolic compound and antioxidant properties (DPPH and FRAP) of pumpkin seeds cookies. All pumpkin seeds cookies showed higher total phenolic compound as compared to control (market wheat flour). This is because of higher total phnolic compound present in pumpkin seeds samples. As a result, pumpkin seeds powders fortification in wheat flour cookies can be excellent substitute of wheat flour to enrich nutrients. It was observed that pumpkin seeds cookies had higher DPPH scavenging activity as compared to control (market wheat flour). On the other hand, FRAP antioxidant properties of pumpkin seeds cookies samples ranged from 1157.25 to 2079.75 m mole/100g. It was also evaluated that pumpkin seeds cookies had higher FRAP antioxidant properties as compared to control (market wheat flour). The higher values of DPPH and FRAP antioxidant activity of pumpkin seeds fortified cookies samples might be due to the presence of higher phenolic compounds in pumpkin seeds.

Table 10: Total phenolic compound and antioxidant properties (DPPH and FRAP) of pumpkin seeds cookies

Cookie Samples	Total Phenolic Compound (mg/100g)	DPPH scavenging activity (mmole/100g)	FRAP Ferric reducing power assay (mmole/100g)
A	24.51^{a}±0.57	162.52^{ab}±12.47	1187.25^{b}±58.33
B	82.57^{a}±0.01	117.97^{b}±18.09	1157.25^{b}±26.51
C	26.37^{a}±0.22	196.35^{ab}±39.09	1269.75^{b}±47.72
D	71.75^{a}±2.74	286.64^{a}±22.46	2079.75^{a}±79.54
E	60.28^{a}±1.14	179.00^{ab}±5.40	1873.50^{a}±265.1

Values are means of triplicate analysis ± Standard deviation. A=Control (100% wheat, Cookie), B=95%wheat/5%pumpkin seed cookie, C=90%wheat/10%pumpkin seed cookie, D=85%wheat/15% pumpkin seed cookie, E=80%wheat/20%pumpkin seed cookie)
All values are express as mean ± SD.
Mean followed by different superscript letters in each column are significantly different ($p<0.05$)

3.5.3 Total Phenolic Compound and Antioxidant Properties (DPPH scavenging activity and FRAP Ferric reducing power assay) of pumpkin peels cookies

Table 11 represents the total phenolic compound and antioxidant properties (DPPH and FRAP) of pumpkin peels cookies. All pumpkin peels cookies showed higher total phenolic compound as compared to control (market wheat flour). This is because of higher total phenolic compound present in pumpkin peels samples. DPPH scavenging activity of pumpkin peels cookies samples ranged from 111.50 to 199.29 mmole/100g. The highest amount of DPPH properties (199.29) was observed for the cookie sample containing 20% peel powder (sample D). Ferric reducing power (FRAP) assay was evaluated for pumpkin peels cookies which was ranged from 1029.75 to 1187. 25 mmole/100g. It was evaluated that the market wheat flour (control) had the highest (1187.25 mmole/100g) F RAP value.

Table 11: Total phenolic compound and antioxidant properties (DPPH and FRAP) of pumpkin peels cookies

Cookie Samples	Total Phenolic Compound (mg/100g)	DPPH scavenging activity (mmole/100g)	FRAP Ferric reducing power assay (mmole/100g)
A	$24.51^{b}\pm0.57$	$162.52^{a}\pm12.47$	$1187.25^{b}\pm58.33$
B	$80.47^{a}\pm2.51$	$138.55^{a}\pm13.51$	$1048.50^{a}\pm63.63$
C	$46.48^{ab}\pm1.02$	$111.50^{a}\pm7.27$	$1063.50^{a}\pm30.75$
D	$60.45^{ab}\pm5.02$	$199.294^{a}\pm25.37$	$1029.75^{a}\pm5.30$

Values are means of triplicate analysis ± Standard deviation. A=Control (100% wheat, Cookie), B=95%wheat/5%pumpkin peel cookie, C=90%wheat/10%pumpkin peel cookie, D=80%wheat/20%pumpkin peel cookie)
All values are express as mean ± SD.
Mean followed by different superscript letters in each column are significantly different ($p<0.05$)

3.5.4 Total Phenolic Compound and Antioxidant Properties (DPPH) of noodles samples

Table 12 displayed total phenolic compound of all noodles sample ranged from 9.55 to 34.96 mg/100g. Sample B had the highest total phenolic compound (34.96 mg/100g). On the other hand, DPPH scavenging activity of all noodles sample ranged from 14.03 to 27.08 mmole/100g. Sample C had the highest DPPH scavenging activity (27.08 mmole/100g). All noodles samples had higher DPPH scavenging activity than control. It indicated that wheat flour can be fortified with pumpkin seeds and peels powders to enrich nutritional properties specially antioxidant activities.

Table 12: Total phenolic compound and DPPH antioxidant properties of noodles samples.

Noodles Samples	Total Phenolic Compound (mg/100g)	DPPH scavenging activity (mmole/100g)
A	18.86 ± 0.49^{ab}	14.03 ± 2.54^{b}
B	34.96 ± 0.27^{a}	16.28 ± 1.79^{b}
C	21.82 ± 1.09^{ab}	27.08 ± 1.27^{a}
D	9.55 ± 1.09^{b}	18.75 ± 1.42^{ab}

Values are means of triplicate analysis ± Standard deviation. A=Control (100% wheat flour noodles), B=90%wheat/10% pumpkin seeds noodles, C=90%wheat/10% pumpkin peels noodles, D=80%wheat/10%pumpkin seeds and 10% pumpkin peels noodles)
All values are express as mean ± SD.
Mean followed by different superscript letters in each column are significantly different ($p<0.05$)

3.6 Hydration properties of powder samples:

Hydration properties such as water absorption index, water solubility index and swelling capacity of market wheat flour, pumpkin seed powder and pumpkin peel powder are shown in Table 13. The values of all powder samples ranged from 187.40 to 764.60 g/100g, 5.60 to 17.60 ml/100g and 5.93 to 21.37 ml/100g for water absorption index, water solubility index and swelling capacity, respectively. The data showed pumpkin peels powder and seeds powder had hydration properties which is close to the market wheat flour (control) sample. It indicates that these two-powder ingredients (seeds powder and peels powder) can be replaced by wheat flour for making less gluten cookie.

Table 13: Hydration Properties (water absorption index, water solubility index and swelling capacity of powder samples)

Cookie Samples	Water Absorption Index (g/100g)	Water Solubility Index (ml/100g)	Swelling Capacity (ml/100g)
Wheat Flour	764.60 ± 20.64^{a}	17.60 ± 1.13^{a}	21.37 ± 1.66^{a}
Pumpkin seed powder	240.40 ± 6.70^{b}	11.20 ± 0.56^{b}	9.53 ± 0.71^{b}
Pumpkin peel powder	187.40 ± 0.20^{b}	5.60 ± 0.01^{c}	5.93 ± 0.01^{c}

All values are express as mean ± SD.
Mean followed by different superscript letters in each column are significantly different ($p<0.05$)

3.7 Sensory evaluation

Twelve judges evaluated cookies and noodles made with different ratios of pumpkin powder based on texture, aroma, color, taste, and overall acceptability. Tables 13, 14, and 15 present mean scores for each criterion. Two-way ANOVA for color preference indicated significant differences ($p<0.05$) among the samples. However, DMRT showed no significant taste differences in pumpkin peel cookies. Among peel cookies, sample S3 scored highest in color, aroma, texture, and overall acceptability. In pumpkin seed cookies, sample S4 with 15% powder achieved the highest scores. For noodles, sample S3 with 10% pumpkin peel powder outperformed others, securing the highest scores in color, texture, aroma, taste, and overall acceptability. The findings suggest optimal ratios for enhancing sensory attributes in pumpkin-enriched cookies and noodles.

Table 14 Mean score for texture, aroma, color, taste and overall acceptability of Pumpkin peel cookies.

Cookie's type	Color	Aroma	Taste	Texture	Overall acceptability
Sample S_1	7.67^{a}	7.00^{ab}	7.00^{a}	7.33^{a}	7.33^{b}
Sample S_2	7.67^{a}	6.58^{bc}	6.91^{a}	7.33^{a}	7.66^{ab}
Sample S_3	8.00^{a}	7.33^{a}	7.67^{a}	8.00^{a}	8.33^{a}
Sample S_4	6.67^{b}	6.33^{c}	7.17^{a}	6.33^{b}	6.00c

All values are express as mean; Mean followed by different superscript letters in each column are significantly different ($p<0.05$)

Sample S_1 = Control (Market Flour); Sample S_2 = 05% pumpkin peel powder; Sample S_3 = 10% pumpkin peel powder; Sample S_4 = 20% pumpkin peel powder

Table 15 Mean score for texture, aroma, color, taste and overall acceptability of Pumpkin seed cookies.

Cookie's type	Color	Aroma	Taste	Texture	Overall acceptability
Sample S_1	6.42^{b}	7.33^{ab}	7.00^{b}	7.33^{a}	7.33^{b}
Sample S_2	6.58^{b}	6.67^{b}	6.33^{b}	6.33^{b}	7.33^{b}
Sample S_3	6.33^{b}	6.67^{b}	6.67^{b}	7.00^{ab}	7.00^{b}
Sample S_4	7.50^{a}	7.67^{a}	8.00^{a}	7.67^{a}	8.33^{a}
Sample S_5	6.58^{b}	7.00^{ab}	6.67^{b}	7.00^{ab}	7.33^{b}

All values are express as mean; Mean followed by different superscript letters in each column are significantly different ($p<0.05$)

Sample S_1 = Control (Market Flour); Sample S_2 = 05% pumpkin seed powder; Sample S_3 = 10% pumpkin seed powder; Sample S_4 = 15% pumpkin seed powder; Sample S_5 = 20% pumpkin seed powder

Table 16 Mean score for texture, aroma, color, taste and overall acceptability of Pumpkin seed and peel noodles.

Noodles type	Color	Aroma	Taste	Texture	Overall acceptability
Sample S_1	7.67^{a}	7.25^{a}	7.00^{a}	7.33^{a}	7.33^{bc}
Sample S_2	7.67^{a}	7.33^{a}	7.00^{a}	7.33^{a}	7.67^{ab}
Sample S_3	8.00^{a}	7.67^{a}	7.67^{a}	8.00^{a}	8.33^{a}
Sample S_4	6.67^{b}	6.33^{b}	6.00b	6.33b	6.58^{c}

All values are express as mean; Mean followed by different superscript letters in each column are significantly different ($p<0.05$) Sample S_1 = Control (Market Flour); Sample S_2 = 10% pumpkin seed powder noodles; Sample S_3 = 10% pumpkin peel powder noodles; Sample S_4 = 10% pumpkin seed powder + 10% pumpkin peel powder noodles

Minerals content

Table 17 outlines mineral content variations in different noodle samples, with significant differences observed in phosphorus, calcium, magnesium, zinc, and iron levels. Notably, 10% pumpkin seed powder noodles exhibited the highest calcium content, while pumpkin peel powder noodles had elevated magnesium concentrations. Zinc and iron levels were notably higher in samples with pumpkin seed and peel powders. These findings underscore the potential of these additives to enhance noodle nutrition. The study suggests that adjusting pumpkin-derived ingredient types and concentrations can optimize mineral enrichment, making these noodles a nutritious alternative to traditional counterparts. The research provides valuable insights into the mineral content of pumpkin-enriched noodles, encouraging further exploration of formulations for optimal nutritional benefits in staple foods.

Table 17: Mean score for mineral content of Pumpkin seed and peel noodles.

Sample	P (mg/kg)	Ca (mg/kg)	Mg (mg/kg)	Zn (mg/kg)	Fe (mg/kg)
**S1**	309.5 ± 0.990^{b}	7050.25 ± 3.748^{a}	7176.7 ± 2.687^{a}	37.82 ± 1.329^{b}	115.7 ± 1.216^{a}
**S2**	686.7 ± 5.515^{ab}	4953.9 ± 6.081^{b}	4958 ± 1.131^{b}	55.62 ± 1.329^{ab}	307.915 ± 1.096^{a}
**S3**	444.1 ± 5.798^{ab}	6025.6 ± 3.960^{ab}	6135 ± 21.213^{ab}	39.88 ± 0.509^{b}	78.94 ± 1.216^{a}
**S4**	816.5 ± 2.970^{a}	5690.5 ± 3.253^{ab}	5494 ± 5.091^{ab}	68.64 ± 0.735^{a}	131.61 ± 0.778^{a}

All values are express as mean; Mean followed by different superscript letters in each column are significantly different ($p<0.05$) Sample S_1 = Control (Market Flour); Sample S_2 = 10% pumpkin seed powder noodles; Sample S_3 = 10% pumpkin peel powder noodles; Sample S_4 = 10% pumpkin seed powder + 10% pumpkin peel powder noodles

4. Conclusions

The production of a pumpkin seeds and peels enriched makes any kinds of confectionary product more nutritious which we have enough satisfactory technological and sensory characteristics. The effects of seeds and peels on the physicochemical and nutritional characteristics of different confectionary products such as cookies and noodles were investigated. Pumpkin seed and peel powders could be used not only enrich nutritious value but also to enhance the quality of food products texture and other rheological properties such as color and flavor. The increase in fibre and ash content in the cookies and noodles showed qualitative and quantitative advantages when compared to the market flour. The sensory acceptability rate reached 78%, which refers to “Good” in the hedonic rating scale, which is considered good acceptability. The results demonstrate production viability for this alternative food due to its very low cost and easy to preparation but more importantly, it can be destined to different meals because it can improve the nutritional life.

References

[1] Norfezah, M.N., Hardacre, A. and Brennan, C.S., 2011. Comparison of waste pumpkin material and its potential use in extruded snack foods. Food Science and Technology International, 17(4), pp.367-373.

[2] Statistics, B.B.O., 2011. Statistical yearbook of Bangladesh. Statistics Division, Ministry of Planning, Dhaka, Government of the People’s Republic of Bangladesh.

[3] El-Adawy, T.A. and Taha, K.M., 2001. Characteristics and composition of watermelon, pumpkin, and paprika seed oils and flours. Journal of agricultural and food chemistry, 49(3), pp.1253-1259.

[4] Brennan, C.S. and Samyue, E., 2004. Evaluation of starch degradation and textural characteristics of dietary fiber enriched biscuits. International Journal of Food Properties, 7(3), pp.647-657.

[5] Fagan, C.C., O’Donnell, C.P., Cullen, P.J. and Brennan, C.S., 2006. The effect of dietary fibre inclusion on milk coagulation kinetics. Journal of Food Engineering, 77(2), pp.261-268.

[6] Tudorica, C.M., Jones, T.E.R., Kuri, V. and Brennan, C.S., 2004. The effects of refined barley β- glucan on the physico- structural properties of low- fat dairy products: curd yield, microstructure, texture and rheology. Journal of the Science of Food and Agriculture, 84(10), pp.1159-1169.

[7] Malkanthi, H.H.A. and Umadevi, S.H., 2018. Effect of dried pumpkin pulp and seed powder on physical, chemical and sensory properties of biscuits. Int J Sci Res, 7, pp.32-4.

[8] Kim, M.Y., Kim, E.J., Kim, Y.N., Choi, C. and Lee, B.H., 2012. Comparison of the chemical compositions and nutritive values of various pumpkin (Cucurbitaceae) species and parts. Nutrition research and practice, 6(1), pp.21-27.

[9] Adams, G.G., Imran, S., Wang, S., Mohammad, A., Kok, S., Gray, D.A., Channell, G.A., Morris, G.A. and Harding, S.E., 2011. The hypoglycaemic effect of pumpkins as anti-diabetic and functional medicines. Food Research International, 44(4), pp.862-867.

[10] Yadav, M., Jain, S., Tomar, R., Prasad, G.B.K.S. and Yadav, H., 2010. Medicinal and biological potential of pumpkin: an updated review. Nutrition research reviews, 23(2), pp.184-190.

[11] AOAC (1998). Official Methods of Analysis of AOAC International (14th ed.). Washington, DC, USA: Association of Official Analytical Chemistry. Methods, 950.46, 938.08, 960.39, 955.04

[12] Navacchi, M.F.P., de Carvalho, J.C.M., Takeuchi, K.P. and Danesi, E.D.G., 2012. Development of cassava cake enriched with its own bran and Spirulina platensis. Acta Scientiarum. Technology, 34(4), pp.465-472.

[13] Akter, M.S., Ahmed, M. and Eun, J.B., 2010. Solvent effects on antioxidant properties of persimmon (Diospyros kaki L. cv. Daebong) seeds. International journal of food science & technology, 45(11), pp.2258-2264.

[14] Mestres, C., Colonna, P. and Buléon, A., 1988. Characteristics of starch networks within rice flour noodles and mungbean starch vermicelli. Journal of food science, 53(6), pp.1809-1812.

[15] Hussain, A., Kausar, T., Sehar, S., Sarwar, A., Ashraf, A.H., Jamil, M.A., Noreen, S., Rafique, A., Iftikhar,

K., Quddoos, M.Y. and Aslam, J., 2022. A Comprehensive review of functional ingredients, especially bioactive compounds present in pumpkin peel, flesh and seeds, and their health benefits. Food Chemistry Advances, p.100067.

[16] Dhiman, A.K., Bavita, K., Attri, S. and Ramachandran, P., 2018. Preparation of pumpkin powder and pumpkin seed kernel powder for supplementation in weaning mix and cookies. International Journal of Chemical Studies, 6(5), pp.167-175.

[17] Syam, A., Zaenal, Y.K., Aulia, N.A., Wati, I.P. and Mansur, M.A., 2019. Development and biochemical analysis of pumpkin seed (Cucurbita moschata Durch) biscuits. Pak J Nutr, 18(8), pp.743-6.

[18] Mishra, S. and Sharma, K., 2019. Development of pumpkin peel cookies and its nutritional composition. Journal of Pharmacognosy and Phytochemistry, 8(4), pp.370-372.

[19] Saleh, S.M. and Ali, S., 2020. Physico-functional properties, nutritional quality, and sensory characteristics of pumpkin peel puree fortified biscuit. Egyptian Journal of Food Science, 48(2), pp.203-212.

[20] Von Lintig, J., 2010. Colors with functions: elucidating the biochemical and molecular basis of carotenoid metabolism. Annual review of nutrition, 30, pp.35-56.

[21] Shami, N.J.I.E. and Moreira, E.A.M., 2004. Lycopene as an antioxidant agent. Revista de nutrição, 17(2), pp.227-236.

[22] Saura-Calixto, F. and Goñi, I., 2006. Antioxidant capacity of the Spanish Mediterranean diet. Food chemistry, 94(3), pp.442-447.

[23] Pierson, J.T., Dietzgen, R.G., Shaw, P.N., Roberts- Thomson, S.J., Monteith, G.R. and Gidley, M.J., 2012. Major Australian tropical fruits biodiversity: Bioactive compounds and their bioactivities. Molecular nutrition & food research, 56(3), pp.357-387.

[24] Lee, C.H., Cho, J.K., Lee, S.J., Koh, W., Park, W. and Kim, C.H., 2002. Enhancing β- carotene content in Asian noodles by adding pumpkin powder. Cereal chemistry, 79(4), pp.593-595.

[25] Godoy, H.T. and Rodriguez-Amaya, D.B., 1998. Occurrence of cis isomers of provitamins A in Brazilian vegetables. Journal of Agricultural and Food Chemistry, 46(8), pp.3081-3086.

[26] Maria do Socorro, M.R., Alves, R.E., de Brito, E.S., Pérez-Jiménez, J., Saura-Calixto, F. and Mancini-Filho, J., 2010. Bioactive compounds and antioxidant capacities of 18 non-traditional tropical fruits from Brazil. Food chemistry, 121(4), pp.996-1002.

[27] Park, W., Shelton, D.R., Peterson, C.J., Wehling, R.L. and Kachman, S.D., 1997. Evaluation of Korean raw noodle (Saeng Myon) color and cooking properties among hard red winter and hard white wheat samples. Food Science and Biotechnology, 6(1), pp.20-25.

[28] Yap, E.S.P., Uthairatanakij, A., Laohakunjit, N. and Jitareerat, P., 2022. Influence of hot air drying on capsaicinoids, phenolics, flavonoids and antioxidant activities of 'Super Hot' chilies. PeerJ, 10, p.e13423.
