# Supplementary material for: Enhancing nutritional value and quality of cookies through pumpkin peel and seed powder fortification
Source: PLoS One. 2025 Feb 27;20(2):e0307506. doi: 10.1371/journal.pone.0307506 (PMC11867389; doi:10.1371/journal.pone.0307506)
Supplement: Attachment — Submitted filename: PONE-D-23-44103_R1 (1).pdf [file pone.0307506.s004.pdf]

PLOS ONE

Enhancing Nutritional Value and Quality of Cookies through Pumpkin Peel and Seed Powder Fortification

--Manuscript Draft--

**Manuscript Number:**	PONE-D-23-44103R1
**Article Type:**	Research Article
**Full Title:**	Enhancing Nutritional Value and Quality of Cookies through Pumpkin Peel and Seed Powder Fortification
**Short Title:**	Enhancing Nutritional Value and Quality of Cookies through Pumpkin Peel and Seed Powder Fortification
**Corresponding Author:**	Md. Asaduzzaman, M. Engg.<br>State University of Bangladesh<br>Dhaka, BANGLADESH
**Keywords:**	Pumpkin, Seeds, Peels, Cookies, Noodles, Nutritional Properties.
**Abstract:**	Background: Recently, the pumpkin by-products such as peels and seeds become more and more interesting for confectionary sector, because of the nutritional and bioactive composition. The study was conducted to develop cookies and noodles using different pumpkin by-products (such as peels and seeds) powders in different concentrations and to evaluate the changes in quality characteristics. The powders were prepared from pumpkin seeds and peels.<br>Methodology: In this study, various concentrations of pumpkin peel powder (5%, 10%, and 20%) and seeds powder (5%, 10%, 15%, and 20%) were mixed with commercially available wheat flour to produce cookies and noodles. Furthermore, the nutritional, antioxidant, functional and sensory properties of the developed cookies and noodles were evaluated.<br>Result: Pumkin peels and seeds powder and their respective products enrich significantly in protein, fiber, beta-carotene, antioxidants such as total phenolic content (TPC), 2,2- diphenyl-1-picrylhydrazyl (DPPH scavenging activity), Ferric reducing ability (FRAP Ferric reducing power assay), ash content and hunter color values. However, pumpkin seed powder had higher protein content (33.25%) than other powders but pumpkin peel powder (16.83%±1.74) had better fiber content than seeds powder. The highest amount of fiber content was found in 10% pumpkin peel cookies. . The analysis of the composition of pumpkin peels and seeds powder has been done in order to further promote their functionality in bakery products based, especially, on wheat flour.<br>Conclusion: The best quality and overall acceptable product were obtained when samples were 10% pumpkin peel powder, 15% pumpkin seed powder, and, 10% pumpkin peel powder noodles.
**Order of Authors:**	Md. Asaduzzaman, M. Engg.
	Mahbubur Rahman, B. Sc.
	Mst. Sorifa Akter, Ph.D.
**Opposed Reviewers:**	
**Response to Reviewers:**	Detailed response to reviewers<br>Manuscript Reference: PONE-D- 23-44103<br>Manuscript Title: Enhancing Nutritional Value and Quality of Cookies through Pumpkin Peel and Seed Powder Fortification.<br>Author(s): Md. Asaduzzaman1,2, Mahbubur Rahman1, Mst. Sorifa Akter3*<br>1)Department of Food Engineering and Technology, State University of Bangladesh, Dhaka, Bangladesh.<br>2)Department of Food Processing and Preservation, Hajee Mohammad Danesh Science and Technology University, Dinajpur, Bangladesh<br>3)Department of nutrition and food Engineering, Daffodil International University, Dhaka, Bangladesh.<br>*Corresponding Author

Powered by Editorial Manager® and ProduXion Manager® from Aries Systems Corporation

Dear Editor and Reviewers,
Thank you very much for your constructive and useful suggestions. Those comments were valuable and very helpful for revising and improving our paper, aswell as for increasing the significance of our research. The responses to editor andreviewer's comments/suggestions of our manuscript have been answered carefullypoint-by-point as follows. Some minor mistakes within the manuscript have alsobeen corrected.
Note: All the line numbers in our responses refer to the revised manuscript. Thered color in the manuscript indicates the changes made during revision.

Response to reviewers
Reviewer #1:
Thank you for your positive perspective on the manuscript.
The comments of the reviewer
Title:
Is it really going to be "A Wheat Flour Replacement Approach"? It's tough to say with such a low amount of seeds and peel powder. Besides, pumpkin seeds are very small amounts, considering the massive amount of wheat flour produced worldwide. I suggest deleting this portion: "A Wheat Flour Replacement Approach."
Comments of the answer:
According to the suggestion of the reviewer, "A Wheat Flour Replacement Approach" is deleting from the title.
Abstract:
-"Significant differences (p<0.05) in protein, fibre, beta……."---This statement is not clear. For this sentence, we do not know if the amounts are higher or lower or if they have a positive or negative impact on product quality. Please make it clear.
As you mentioned % of peel, seed, etc., in the results and conclusion of the abstract, you should add this to the methodology, too.
You have also mentioned Overall acceptability in the results of the abstract but did not mention it in the methods of the abstract.
Comments of the answer:
The abstract is rewritten according to the suggestion of the reviewer 01. The methodology is added in the abstract.
Introduction:
-Use updated data. You used data from 2005, and now it's 2024!
-The introduction section is very short; it lacks literature study and references. The novelty of the work has not been stated properly.
Comments of the answer:
The updated data added in the introduction section and data are updated. There more literature and references added in the introduction.

Material and Methods:
-Section 2.2: Were the seeds husked before milling?
Comments of the answer: we did not husked the seeds before milling, we add the husked to in the cookies and noodles development.
-Using both Figures 1 and 2 is unnecessary. You can merge them and mention the seed/peel portion in different samples. Otherwise, you may discard both of the figures and illustrate them in your writing.
Comments of the answer: While it is true that both figures share many commonalities, combining them would result in overly complicated design and complexity in understanding. Hence, we have chosen to maintain them as separate figures. However, should you suggest amalgamating them into one figure; we will provide detailed representation of the leveling process in the methodology section.
The samples in Figures 1 to 3 originally had disparate labeling, which has now been standardized uniformly under the same notation.
SA = Control (100% market wheat flour, Cookie)

Powered by Editorial Manager® and ProduXion Manager® from Aries Systems Corporation

SB = 95% market wheat flour /5%pumpkin seed cookie
SC = 90% market wheat flour /10%pumpkin seed cookie
SD = 85% market wheat flour /15%pumpkin seed cookie
SE = 80%market wheat flour /20%pumpkin seed cookie
PA = Control (100% market wheat flour, Cookie)
PB = 95%wheat/5%pumpkin peel cookie
PC = 90%wheat/10%pumpkin peel cookie
PD = 80%wheat/20%pumpkin peel cookie
NA = Control (100% wheat flour noodles)
NB = 90%wheat/10% pumpkin seeds noodles
NC = 90%wheat/10% pumpkin peels noodles
ND = 80%wheat/10%pumpkin seeds and 10% pumpkin peels noodles

-Section 2.4 and 2.5: Where does sweet potato powder come here?
Comments of the answer: Mistakenly we wrote "sweet potato peel" which corrected to pumpkin seeds powder, peels powders, cookies and noodles in the section 2.4 and 2.5.
-Section 2.4: If the Protein determination process is through the Kjeidhal method, please mention the N conversion factor.
Comments of the answer: In the section 2.4 – the N conversion factor is added.
-Section 2.5-2.9: You must have to add all the details about the methodology. These methods are not reproducible and not suitable for publication.
Comments of the answer: according to your suggestion all section 2.5 to 2.8 are described. One section is added to the previous section, so that, one section is minimized.
-Section 2.10: The sensory analysis should be vividly described. Also, the number of panellists needs to be higher. Add whether they were trained/semi-trained, how samples were presented to them, etc. The statistical analysis portion should be moved to section 2.11.
Comments of the answer: The sensory analysis methodology has been updated in accordance with the reviewer's suggestions. The statistical analysis section has been relocated to Section 2.10. All panelists are faculty members of the Department of Food Engineering and Technology, and prior to the sensory analysis, they underwent training for the tests. I regret the limited number of panelists; however, currently, our department lacks qualified sensory panelists. We are actively working to increase the number of panelists and are organizing training sessions to recruit and train new members.
-Section 2.11: Mention how many replicates for each observation were used.

Comments of the answer: All evaluations of samples were all done in triplicate.

Results and Discussion:
-Table 01: Check 3.9.1±0.48b?

Comments of the answer: the data was corrected. It will be 3.91±0.48b
-In methodology, you mentioned Sample 1, 2, 3, etc…in Results and Discussion, you are saying Sample A, B, C, etc…
Comments of the answer: The sample indications were wrong which was corrected and described according to the data in the methodology section.
-If every result of your study is comparable to those of Syam et al. (2019), then what is the justification of your study?
Comments of the answer: Along with syam et al. (2019), more literature added in the section 3.1.2. such as Alshehry (2020) and Apostol et al. (2020)

-Where is the Carbohydrate content in your results???
Comments of the answer: There are no carbohydrate discussions in this study, so the carbohydrate portion was removed from methodology.

-Section 3.2: "The antioxidant capacities of leaves????????? may vary………..”? You should be careful while writing a manuscript for publication.
Comments of the answer: thank you the suggestion, we will be more careful to write the manuscript for publishing in this renowned journal.

-Section 3.3: Add the units of cooking loss and cooking yield. From the standard deviation, it seems the indications of significant differences are not correct.
Comments of the answer: We add more literature in this section to differentiate differences.

-Table 12: The results of sample D are not homogenous with the other results. Besides, where are the FRAP results of Noodles?
Comments of the answer: The description is corrected and due to the some difficulties in the laboratory the FRAP test are not include for the noodles sample.

-Section 3.6: You should mention the significance of Hydration properties. The discussion of this section is also inadequate. Like other parameters, you didn't measure the hydration properties of your samples.
Comments of the answer: in this section firstly we discussed the importance of the hydration properties and then we add more discussion and literatures.

-Section 3.7: Previously, you mentioned 10 panellists!
Comments of the answer: Mistakenly we count our self as a panelist which was corrected.

-Table 14, 15, 16: You need to add the standard deviations in the results.
-Besides, this section lacks discussion.
Comments of the answer: The standard deviation is added in table 14 to 16. Description and literature is also added.

-Minerals content: You did not mention this in the methodology. If you would like to keep it, you should add it before sensory evaluation. Obviously, you should add mineral contents to all samples and ingredients as well. You should improve the discussion section also.
Comments of the answer: The section on mineral content has been removed because no other confectionery items besides noodles and samples were examined for minerals, which is not relevant to this study.

Conclusions:
-The first sentence is not clear. Make it simple.
Comments of the answer: The conclusion sentence has been simplified for brevity. The antioxidant activity is discussed in the corresponding section.

References:
Most of the references used in the manuscripts are too old. You should use more updated references.
Comments of the answer: In the references section, updated refinances are added which cited in the study.

Reviewer #2:
P11 2.1.the variety and degree of ripeness should be mentioned. The city of Dhaka should also be mentioned with the country. The wheat flour used should be characterised more precisely, gliadin, protein, starch content or technological property should be mentioned.
Comments of the answer:
In Section 2.1, in compliance with the reviewer's suggestion, Dhaka is now accompanied by the country name in the author's affiliation and the materials and methods section. Additionally, the gluten value was included in the manuscript following the powder test as per the reviewer's recommendation. Furthermore, the scientific name of the pumpkin variety and the degree of ripeness are detailed in the materials and methods section.

Figures 1-3 have different labelling of the samples, there are mixed samples1-4, formula1-4 and in the results are discus samples A-B. This should be standardised. This figure also shows the abbreviation gm, this should be explained in the note, or if it is g, it should be changed.

Powered by Editorial Manager® and ProduXion Manager® from Aries Systems Corporation

	Comments of the answer:<br>Figure 1 to 3 had different labeling of the samples which now converted in to same notation and standardised uniformly where<br>SA = Control (100% market wheat flour, Cookie)<br>SB = 95% market wheat flour /5%pumpkin seed cookie<br>SC = 90% market wheat flour /10%pumpkin seed cookie<br>SD = 85% market wheat flour /15%pumpkin seed cookie<br>SE = 80%market wheat flour /20%pumpkin seed cookie<br>PA = Control (100% market wheat flour, Cookie)<br>PB = 95%wheat/5%pumpkin peel cookie<br>PC = 90%wheat/10%pumpkin peel cookie<br>PD = 80%wheat/20%pumpkin peel cookie<br>NA = Control (100% wheat flour noodles)<br>NB = 90%wheat/10% pumpkin seeds noodles<br>NC = 90%wheat/10% pumpkin peels noodles<br>ND = 80%wheat/10%pumpkin seeds and 10% pumpkin peels noodles<br><br>P13 Figure 2 the weight of the ingredients should be stated separately "Water & eggs 33g".<br>Comments of the answer: In the figure 02, the weight of the ingredients mistakenly wrote water and egg 33g. It should be only egg 33 g which is corrected in the figure.<br><br>P15 2.4 and 25 why is it written "sweet potato peel"?<br>Comments of the answer: Mistakenly we wrote “sweet potato peel” which corrected to pumpkin seeds powder, peels powders, cookies and noodles in the section 2.4 and 2.5.<br><br>P16 For used equipment the manufacturer and country of origin should be stated. The source of the chemical used should also be mentioned.<br>Comments of the answer: The equipment manufacturer and origin are added in the 2.2 section including country. The source of the chemical used in the study also included in the section 2.1.<br><br>P17 Table 1 shows no statistical differences between the samples, but the mean is different and the SD is low. The author should explain this.<br>Comments of the answer: The statistical differences between the samples are corrected and the explanation is added in the section 3.1.1.<br><br>P18 biscuits33.05/100g - missing space<br>Comments of the answer: the space is added between biscuits 33.05/100g in the section 3.1.2.<br><br>P18 2nd column (C) and P19 3C - the author should explain how fat oxidation can change fat content or delete this sentence.<br>Comments of the answer: The explanation of fat oxidation is added in the section 3.1.1. This also repeated in the section 3.1.2.<br><br>P18 "Dietary fibre values were significantly different between the cookies and the highest was found in sample D (1.61/100g), while the lowest content (0.32/100g) was investigated in sample A. This observation was comparable to that of Syam et al. (2019) who reported a fat value of 1.64/100g in pumpkin seed cookies [17]." - Correctly described ingredients<br>Comments of the answer: The sample indications were wrong which was corrected and described according to the data.<br><br>P21 Chapter 3.2 should be part of 3.1 as in previous biscuits.<br>Comments of the answer: chapter 3.2 is changed to 3.1.5<br><br>P22 Table 05 sample labelling should be identical in manuscript,<br>Comments of the answer: Table 05 sample labeling is corrected and wrote according to the formulation figure and data.<br><br>P23 Table 06 use the same orientation in the cell, for Delta E there are no significant

differences but the values are different and the SD is low, this should be explained.
Comments of the answer: in the table 06, the leveling data is corrected and the explanations are added in the section 3.3.4. More literature is added describe in the section.

P24 1C I did not agree that seeds are a better source of polyphenols. This should be confirmed by literature or discussed in more detail. In my opinion there are interferences with proteins in seeds.
Comments of the answer: the pumpkin peels are more better source of the total phenolic compounds then pumpkin seeds which was discussed in the section 3.4.1
P24 Table 09 were there no statistical differences?
Comments of the answer: the statistical significance added in the table 09 and discussed in the section 3.4.1.

P24 3.5.2 and Table 10 should explain why Sample C has the lowest TPC and the highest antioxidant activity, this is not consistent with the results in Table 09.
Comments of the answer: the value of the sample Sc was incorrectly imputed.

P25 F RAP value - correct to FRAP value.
Comments of the answer: the “F RAP value” corrected to “FRAP value”.

P25 The sentence "showed higher total phenolic compound compared to control" is misleading, the statistical differences were not found for all samples.
Comments of the answer: the sentence was corrected.

P26 3.5.4 and Table 12 do not describe FRAP, it should be stated why it is not used or added.
Comments of the answer: Due to the some difficulties in the laboratory the FRAP test are not include for the noodles sample.

P27 The fact that the water absorption index, water solubility index and swelling capacity are high does not mean that they are capable of forming a 3D network. The sentence "It indicates that these two powdered ingredients (seed powder and hull powder) can be replaced by wheat flour for making less gluten biscuits" should be more clearly described or deleted.
Comments of the answer: in the section 3.5, the sentence was changed and more literature and description was added.

P28 Tables 14 and 15: standardise the labelling of the samples, 6.00c- use the superscript.
Comments of the answer: the tables labeling and the superscript are corrected.
P29 Effect of mineral compounds on nutrition should be discussed with literature.
Comments of the answer: the tables labeling and the superscript are corrected.

P30 Conclusion, antioxidant activity should be mentioned, rheological properties were not instrumentally analysed so should not be mentioned in the conclusion.
Comments of the answer: The sentence of the conclusion describe briefly and make it simple. The antioxidant activity is mentioned in the section.

Powered by Editorial Manager® and ProduXion Manager® from Aries Systems Corporation

	Reviewer #3:<br>Rewrite the abstract, introduction and conclusions.<br>Need to add photographs of the products and raw material used in the materials.<br>Comments of the answer:<br>Thanks you for your positive view about the manuscript. We rewrite the abstract, introduction and conclusion section according to your suggestion. We add more information and references and more literature in the introduction and discussion section.<br>The photo of the samples are added in “Supporting Information files - Photo”
**Additional Information:**	
**Question**	**Response**
**Financial Disclosure**<br><br>Enter a financial disclosure statement that describes the sources of funding for the work included in this submission. Review the submission guidelines for detailed requirements. View published research articles from *PLOS ONE* for specific examples.<br><br>This statement is required for submission and **will appear in the published article** if the submission is accepted. Please make sure it is accurate.<br><br>**Funded studies**<br>Enter a statement with the following details:<br>• Initials of the authors who received each award<br>• Grant numbers awarded to each author<br>• The full name of each funder<br>• URL of each funder website<br>• Did the sponsors or funders play any role in the study design, data collection and analysis, decision to publish, or preparation of the manuscript?<br><br>Did you receive funding for this work?	Yes
Please add funding details.<br>as follow-up to "**Financial Disclosure**<br><br>Enter a financial disclosure statement that describes the sources of funding for the	Special Projects from Ministry of Science and Technology<br>Environmental Science: 382

Powered by Editorial Manager® and ProduXion Manager® from Aries Systems Corporation

work included in this submission. Review the submission guidelines for detailed requirements. View published research articles from *PLOS ONE* for specific examples.<br><br>This statement is required for submission and **will appear in the published article** if the submission is accepted. Please make sure it is accurate.<br><br>**Funded studies**<br>Enter a statement with the following details:<br>• Initials of the authors who received each award<br>• Grant numbers awarded to each author<br>• The full name of each funder<br>• URL of each funder website<br>• Did the sponsors or funders play any role in the study design, data collection and analysis, decision to publish, or preparation of the manuscript?<br><br>Did you receive funding for this work?"	
Please select the country of your main research funder (please select carefully as in some cases this is used in fee calculation).<br>as follow-up to "**Financial Disclosure**<br><br>Enter a financial disclosure statement that describes the sources of funding for the work included in this submission. Review the submission guidelines for detailed requirements. View published research articles from *PLOS ONE* for specific examples.<br><br>This statement is required for submission and **will appear in the published article** if the submission is accepted. Please make sure it is accurate.	BANGLADESH - BD

**Funded studies**<br>Enter a statement with the following details:<br>• Initials of the authors who received each award<br>• Grant numbers awarded to each author<br>• The full name of each funder<br>• URL of each funder website<br>• Did the sponsors or funders play any role in the study design, data collection and analysis, decision to publish, or preparation of the manuscript?<br><br>Did you receive funding for this work?"	
**Competing Interests**<br><br>Use the instructions below to enter a competing interest statement for this submission. On behalf of all authors, disclose any competing interests that could be perceived to bias this work—acknowledging all financial support and any other relevant financial or non-financial competing interests.<br><br>This statement is required for submission and **will appear in the published article** if the submission is accepted. Please make sure it is accurate and that any funding sources listed in your Funding Information later in the submission form are also declared in your Financial Disclosure statement.<br><br>View published research articles from *PLOS ONE* for specific examples.	The authors have declared that no competing interests exist

Powered by Editorial Manager® and ProduXion Manager® from Aries Systems Corporation

**NO authors have competing interests**<br><br>Enter: *The authors have declared that no competing interests exist.*<br><br>**Authors with competing interests**<br><br>Enter competing interest details beginning with this statement:<br><br>*I have read the journal's policy and the authors of this manuscript have the following competing interests: [insert competing interests here]*<br><br>* typeset	
**Ethics Statement**<br><br>Enter an ethics statement for this submission. This statement is required if the study involved:<br><br>• Human participants<br>• Human specimens or tissue<br>• Vertebrate animals or cephalopods<br>• Vertebrate embryos or tissues<br>• Field research<br><br>Write "N/A" if the submission does not require an ethics statement.<br><br>General guidance is provided below. Consult the submission guidelines for detailed instructions. **Make sure that all information entered here is included in the Methods section of the manuscript.**	N/A

**Format for specific study types**<br><br>**Human Subject Research (involving human participants and/or tissue)**<br>• Give the name of the institutional review board or ethics committee that approved the study<br>• Include the approval number and/or a statement indicating approval of this research<br>• Indicate the form of consent obtained (written/oral) or the reason that consent was not obtained (e.g. the data were analyzed anonymously)<br><br>**Animal Research (involving vertebrate animals, embryos or tissues)**<br>• Provide the name of the Institutional Animal Care and Use Committee (IACUC) or other relevant ethics board that reviewed the study protocol, and indicate whether they approved this research or granted a formal waiver of ethical approval<br>• Include an approval number if one was obtained<br>• If the study involved *non-human primates*, add *additional details* about animal welfare and steps taken to ameliorate suffering<br>• If anesthesia, euthanasia, or any kind of animal sacrifice is part of the study, include briefly which substances and/or methods were applied<br><br>**Field Research**<br><br>Include the following details if this study involves the collection of plant, animal, or other materials from a natural setting:<br>• Field permit number<br>• Name of the institution or relevant body that granted permission	
**Data Availability**<br><br>Authors are required to make all data underlying the findings described fully available, without restriction, and from the time of publication. PLOS allows rare exceptions to address legal and ethical concerns. See the PLOS Data Policy and FAQ for detailed information.	Yes - all data are fully available without restriction

A Data Availability Statement describing where the data can be found is required at submission. Your answers to this question constitute the Data Availability Statement and **will be published in the article**, if accepted.<br><br>**Important:** Stating 'data available on request from the author' is not sufficient. If your data are only available upon request, select 'No' for the first question and explain your exceptional situation in the text box.<br><br>Do the authors confirm that all data underlying the findings described in their manuscript are fully available without restriction?	
**Describe where the data may be found in full sentences. If you are copying our sample text, replace any instances of XXX with the appropriate details.**<br><br>• If the data are **held or will be held in a public repository**, include URLs, accession numbers or DOIs. If this information will only be available after acceptance, indicate this by ticking the box below. For example: *All XXX files are available from the XXX database (accession number(s) XXX, XXX.).*<br>• If the data are all contained **within the manuscript and/or Supporting Information files**, enter the following: *All relevant data are within the manuscript and its Supporting Information files.*<br>• If neither of these applies but you are able to provide **details of access elsewhere**, with or without limitations, please do so. For example:<br><br>*Data cannot be shared publicly because of [XXX]. Data are available from the XXX Institutional Data Access / Ethics Committee (contact via XXX) for researchers who meet the criteria for access to confidential data.*<br><br>*The data underlying the results presented in the study are available from (include the name of the third party*	All relevant data are within the manuscript and its Supporting Information files

and contact information or URL).<br>• This text is appropriate if the data are owned by a third party and authors do not have permission to share the data.<br><br>* typeset	
Additional data availability information:	

17th March 2024

Editor-in-Chief

Editorial Department of PLOS One,

PLOS.

Dear Sir,

I am resubmitting a manuscript for consideration of publication in PLOS One. This renowned journal covers important studies on the fortification of natural components and natural bio-active compounds. The manuscript is entitled “Enhancing Nutritional Value and Quality of Cookies through Pumpkin Peel and Seed Powder Fortification” It was submitted to PLOS ONE for consideration of publication on January 3, 2024, and received a major revision on February 16th. I have carefully revised my manuscript in response to the reviewers' comments, and the annotated version has been uploaded to your esteemed journal's dashboard. Additionally, I have meticulously formatted my manuscript following the journal's recommendations and guidelines.

This study was supported by the Ministry of Science and Technology, Government of the Peoples’ Republic of Bangladesh Special Allocation (Grant number ES 382; 2020-2021). The funders had no role in study design, data collection and analysis, decision to publish, or preparation of the manuscript.

I kindly request your prompt attention to the necessary steps for its publication. Thank you very much for your consideration.

Yours Sincerely,

Md. Asaduzzaman

Assistant Professor and academic coordinator,

Department of Food Engineering and Technology,

Kanchan Bridge, Purbacahal 1461, Bangladesh,

State university of Bangladesh.

Orcid ID: 0000-0001-7202-961X

Tel.: +8801710481107

E-mail: bitatipu@gmail.com

Manuscript Click here to access/download;Manuscript;Manuscript.docx

Title: "Enhancing Nutritional Value and Quality of Cookies through Pumpkin Peel and Seed Powder Fortification"

Author List:

1) Md. Asaduzzaman
   Assistant Professor,
   Department of Food Processing and Preservation, Hajee Mohammad Danesh Science and Technology University, Dinajpur, Bangladesh
   Department of Food Engineering and Technology, State University of Bangladesh, Dhaka, Bangladesh.
   Email: bitatipu@gmail.com; mzaman@sub.edu.bd

2) Mahbubur Rahman
   Department of Food Engineering and Technology, State University of Bangladesh, Dhaka, Bangladesh.
   Email:rmahbub896@gmail.com

3) Mst. Sorifa Akter*
   Assistant Professor,
   Department of nutrition and food Engineering, Daffodil International University, Dhaka, Bangladesh.
   Email: sorifa.nfe@diu.edu.bd

* Corresponding Author
Corresponding Author email address: sorifa.nfe@diu.edu.bd

Abstract

Background: Recently, the pumpkin by-products such as peels and seeds become more and more interesting for confectionary sector, because of the nutritional and bioactive composition. The study was conducted to develop cookies and noodles using different pumpkin by-products (such as peels and seeds) powders in different concentrations and to evaluate the changes in quality characteristics. The powders were prepared from pumpkin seeds and peels.

Methodology: In this study, various concentrations of pumpkin peel powder (5%, 10%, and 20%) and seeds powder (5%, 10%, 15%, and 20%) were mixed with commercially available wheat flour to produce cookies and noodles. Furthermore, the nutritional, antioxidant, functional and sensory properties of the developed cookies and noodles were evaluated.

Result: Pumkin peels and seeds powder and their respective products enrich significantly in protein, fiber, beta-carotene, antioxidants such as total phenolic content (TPC), 2,2- diphenyl-1-picrylhydrazyl (DPPH scavenging activity), Ferric reducing ability (FRAP Ferric reducing power assay), ash content and hunter color values. However, pumpkin seed powder had higher protein content (33.25%) than other powders but pumpkin peel powder (16.83%±1.74) had better fiber content than seeds powder. The highest amount of fiber content was found in 10% pumpkin peel cookies. . The analysis of the composition of pumpkin peels and seeds powder has been done in order to further promote their functionality in bakery products based, especially, on wheat flour.

Conclusion: The best quality and overall acceptable product were obtained when samples were 10% pumpkin peel powder, 15% pumpkin seed powder, and, 10% pumpkin peel powder noodles.

Keywords: Pumpkin,Seeds,Peels,Cookies,Noodles,NutritionalProperties.

1. INTRODUCTION

Pumpkin (*Cucurbita moschata*) is a fruit vegetable, not only grown in the Western Hemisphere but also in Tropical Asia in countries such as Bangladesh, India, Indonesia, Malaysia and Philippines [1].The total world agricultural production has increased annually over the years from 111.986 million tons in 2018 to 136.27 million tons in 2020 while in Bangladesh pumpkin cultivation has an annual production of 340 thousand metric tons [1,2].

Pumpkins have a large range of uses as a potentially valuable food source for both humans and animals. The edible flesh (pulp) of the pumpkin fruit may be eaten as a vegetable or incorporated into food products such as curry, pies, bread, soup, or jams. The food industry utilizes pumpkin powders and flakes as thickeners, flavoring and coloring agents in soups and sauces [1, 3]. However, food processing techniques create large quantities of waste in the form of peels (skin) and seeds. These waste streams can be utilized as an added value ingredient with the potential of being processed into food products in a similar way to other waste stream products and fiber-rich components [4-6].

Pumpkin peels and seeds are rich in vitamins, proteins, minerals and antioxidants (β-carotene and tocopherols) [7,8]. In addition to nutrient composition, it is composed of various biological active components such as polysaccharides, protein, peptide, sterols and para-amino benzoic acids [7, 9]. These biological active components have shown to be wide range of medicinal properties such as anti-diabetic activity, antioxidant activity, anti-carcinogenic effect and anti-microbial effect [7, 10].

With these beneficial effects of pumpkin peels and seeds, it can be incorporated into different value-added products such as cookies and snacks (noodles). In recent times, fruits and vegetables by-products have attracted more attention due to their health benefits including pumpkin pie, biscuits, bread, desserts, puddings, beverages, and soups. Pumpkins are celebrated in festivals and in flower and vegetable shows in many countries. Therefore, it has been focused on under-utilized indigenous crops waste, for example the pumpkin peels and seeds are useful in food industries for the formulation of value-added products. "Several previous studies have demonstrated the strong antioxidant activity of pumpkin peels and seeds (Apostol et al., 2017; Stevenson et al., 2007) and their potential role in preventing hypertension and carcinogenic diseases (Zuhair et al., 2000; Jian et al., 2005) [37, 39-41]. Although there have been studies on formulations of cookies and noodles incorporating pumpkin peels and seeds, mixtures of wheat flour with various added ingredients are known to be rich in numerous active principles such as antioxidants, macro elements, microelements, and fiber, making them highly valuable raw materials for the confectionery industry [37].

In recent times, there has been increasing attention toward finding uses for by-products and waste generated from food processing. Utilizing these by-products would not only help maximize available resources but also introduce new food products to the market, while simultaneously addressing waste disposal issues."

Therefore, the objective of this study was to utilize pumpkin by-products and develop pumpkin peels and seeds powders mixed cookies and noodles and evaluation of its physical, chemical and sensory properties.

2. MATERIALS AND METHODS

2.1 **Raw Materials, chemicals and other ingredients**

Fully ripe pumpkins (Cucurbita moschata) were sourced from a local market in Dhaka, Bangladesh chosen for uniformity, color, and lack of defects during July-august, 2023 harvest season. Pumpkin seeds, wheat flour (Local Brand: ACI Pure flour), oil, sugar, skimmed milk powder, butter, eggs, baking powder, salt, and margarine were obtained locally. Citric acid and packaging materials came from a local company. All items, except butter and eggs stored in a refrigerator, were kept at room temperature. High-density polyethylene bags stored peel powder, seed powder, cookies, and noodle samples. All analytical grade chemicals were supplied by the food analytical laboratory and food processing laboratory, State University of Bangladesh, Dhaka.

2.2 Preparation of pumpkin peels and seeds powder

Ripe pumpkin was washed, sliced, and its peels collected. The peels were treated with 0.5% citric acid, dried at 65°C for 8 hours. Purchased seeds were oven-dried at 80°C for 22 hours. Both dried peels and seeds (With husk) were blended by blender machine (Jaipan CM/L - 7360065), sieved by A.S.T.M. (E-11), Endecotts limited, London, England through a 50-mesh size, and packed in high-density polyethylene bags for analysis.

2.3 Preparation of cookies and noodles from pumpkin seeds and peels

2.3.1 Powders Formulation of Cookies

There were two types of cookies were formulated. Cookies were prepared from pumpkin seeds and peels powders. Pumpkin seeds powders and peels powders were substituted at 5%, 10%, 15%, 20% and 5%, 10%, 20%, respectively with marketed wheat flour which were represented in the figure 01 and 02. Cookies prepared from market wheat flour serve as control. The lists of the samples are

S_A = P_A = Control (100% market wheat flour, Cookie)
S_B = 95% market wheat flour /5%pumpkin seed cookie
S_C = 90% market wheat flour /10%pumpkin seed cookie
S_D = 85% market wheat flour /15%pumpkin seed cookie
S_E = 80%market wheat flour /20%pumpkin seed cookie

P_B = 95%wheat/5%pumpkin peel cookie
P_C = 90%wheat/10%pumpkin peel cookie
P_D = 80%wheat/20%pumpkin peel cookie

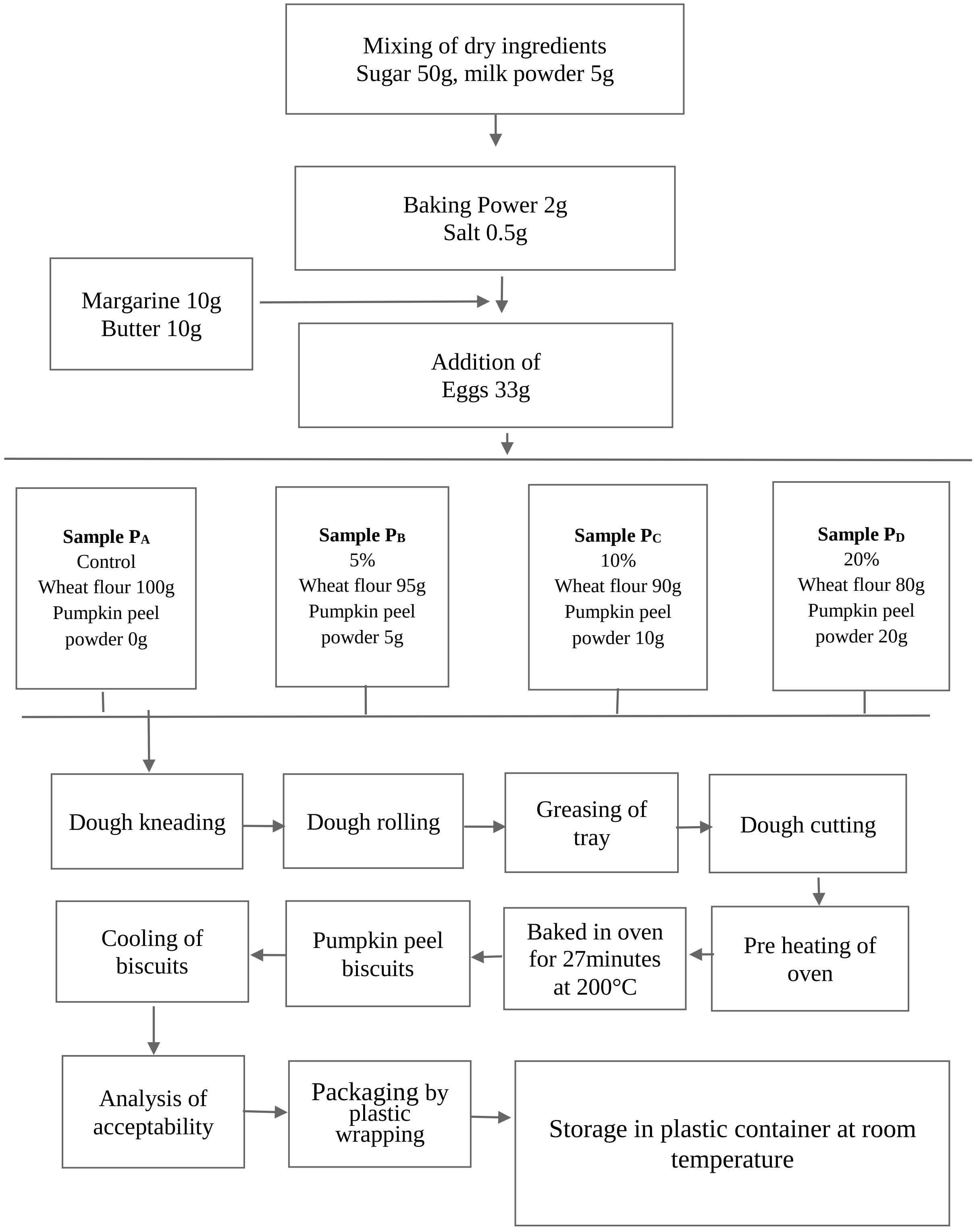

Figure 01: Flow diagram of preparation of pumpkin peel cookies samples

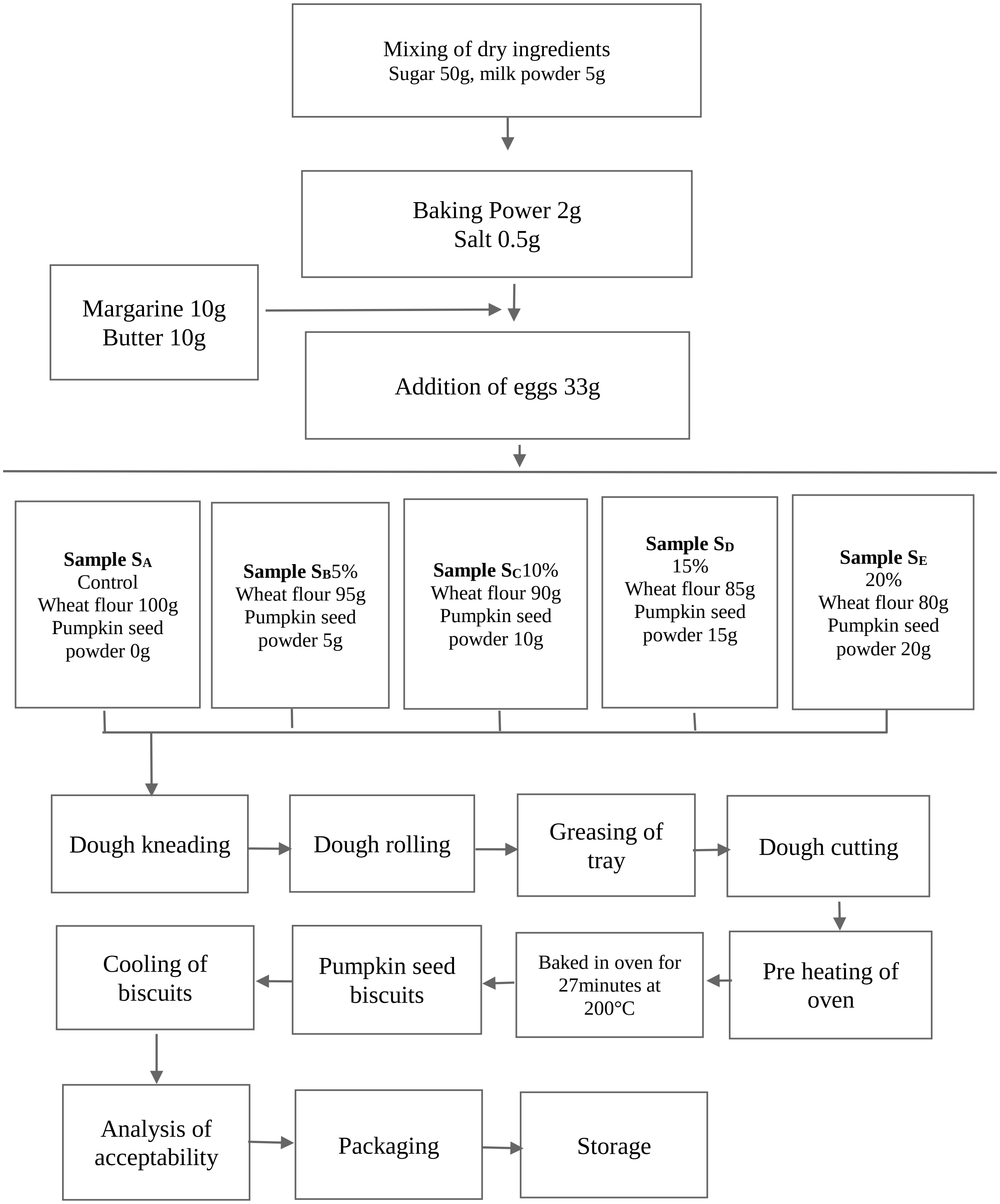

Flow diagram 02: Preparation of pumpkin seed cookies samples

2.3.2 Formulation of Noodles

Noodles were also made from pumpkin seeds powder and pumpkin peels powders. Three types of noodles were formulated such as wheat four fortified with 10% seed powder, 10% peel powder and 10% seed powder mixed with 10% peel powder which were represented in the figure 03.Noodles prepared from market wheat flour serve as control. The list of the noodles samples are

N_A = Control (100% wheat flour noodles)
N_B = 90%wheat/10% pumpkin seeds noodles
N_C = 90%wheat/10% pumpkin peels noodles
N_D = 80%wheat/10%pumpkin seeds and 10% pumpkin peels noodles

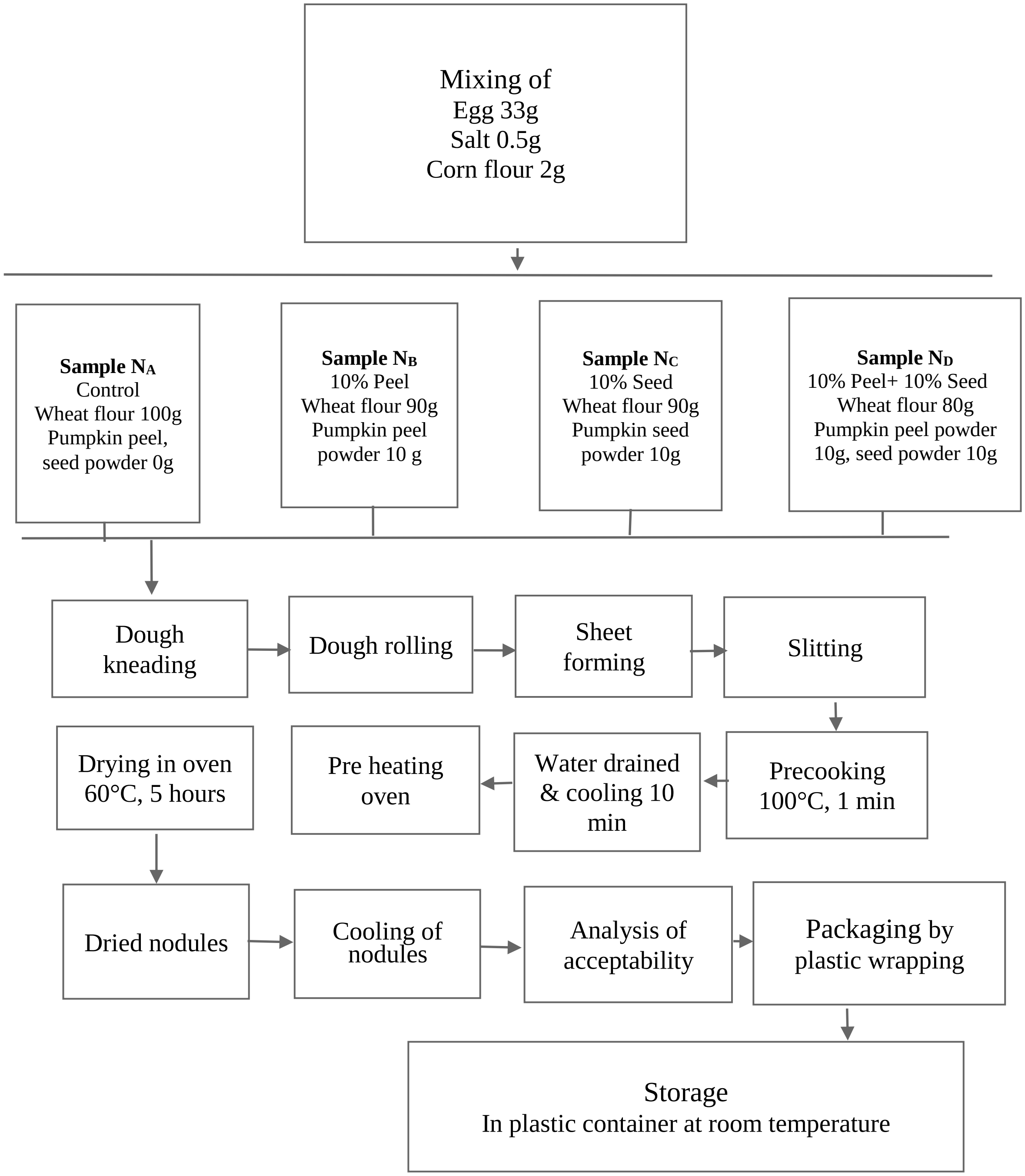

Figure 03: Flow diagram of Preparation of noodles samples

2.4 Proximate composition

Moisture (oven drying method), protein (Kjeldahl method, where the N conversion factor is 6.25), fat (Soxhlet method), ash (Muffle furnace method) and crude fiber content of pumpkin seeds powder, peels powders, cookies and noodles were determined by official methods of Association of Official Analytical chemists (AOAC, 1984; 1998) [11] and the gluten content was determined according to the method reported by Taneya et al. (2014) [30]. .

Acid and alkali hydrolysis followed by ignition of the residue as described by the method AOAC (1984) was used to estimate the fiber content in pumpkin seeds & peels powders and cookies and noodles samples [11]. All the analyses were done in triplicates. Then the fiber content was determined by following equation

$$\%\ of Fibre = \frac{Initial\ weight - Final\ Weight}{Sample\ weight} \times 100$$

2.5 Determination of total polyphenol content

Determination of the total polyphenol content was done by Sorifa et al., (2010) method with some modifications [13]. 1 gram of samples was extracted using 20 ml of 25% ethanol for 15 minutes and subsequently filtered through Whatman no. 2 filter paper. Following this, a mixture of 1 ml of the sample, 1 ml of Folin reagent, and 5 ml of Na_2CO_3 was transferred to a volumetric flask and left at room temperature for 1 hour. Total phenols were calculated based on standard curves of gallic acid and expressed as mg/1 g utilizing spectrophotometer (T80 U/VIS, United Kingdom).

2.6 Determination of DPPH:

The scavenging effects of the samples on 1,1-diphenyl-2-picrylhydrazyl (DPPH) radical were evaluated following a modified method based on the previous report by Sorifa et al. (2010). Each sample (1 milliliter) was combined with 5 milliliters of freshly prepared 0.1 mM DPPH methanolic solution. The resulting mixture was shaken and incubated in darkness for 50 minutes at room temperature. Absorbance was subsequently measured at 517 nm using a spectrophotometer (T80 U/VIS, United Kingdom The DPPH radical scavenging activity was then calculated as follows:

$$Scavenging\ effect\ (\%) = \left[Abs_{control} - \left(Abs_{sample} - Abs_{sample\ backgroung}\right)\right] \times 100/Abs_{control}$$

2.7 Determination of FRAP

The chelating capacity of PSE was assessed using a method outlined by Sorifa et al. (2010). Various concentrations of samples (0.1 mL) were combined with 0.1 mL of 2 mM $FeCl_2 \cdot 4H_2O$ and 0.2 mL of 5 mM ferrozine. Subsequently, 3.7 mL of MeOH was added to the mixture in a test tube and allowed to react for 10 minutes. The absorbance at 562 nm was then measured, and chelating activity was determined using the following equation:

$$Chelating\ activity\ (\%) = \left[Abs_{control} - \left(Abs_{sample} - Abs_{sample\ backgroung}\right)\right] \times 100/Abs_{control}$$

2.8 Cooking properties

The cooking properties of noodles were evaluated by a method similar to that described by Mestres et al., 1988 [14]. Noodles weighing 1g were added to 150mL of boiling water and cooked for 1 minute to achieve optimal cooking. The sample was subsequently drained for 5 minutes and swiftly weighed (W_1, g). The cooked product was then dried in an oven at 130°C until a constant weight was achieved (W_2,

g). The cooking water was centrifuged at 7500 rpm for 10 minutes, and the dry matter contents of the sediment and supernatant (W_3, g and W_4, g, respectively) were determined. These evaluations of cooking properties were all done in duplicate.

Cooking yield and losses were calculated with the following equations (DM = dry matter ratio of crude samples):

$$Cooking\ yield\ (\%) = \frac{(W_2) \times 100}{W_1}$$

$$Cooking\ loss\ (\%) = \frac{(DM - W_2) \times 100}{DM}$$

2.9 Sensory evaluation

A sensory analysis of cookies was conducted using a 9-point Hedonic scale by a panel of 10 members from the Department of Food Engineering and Technology at the State University of Bangladesh, Dhaka. The assessment covered attributes such as color, flavor, taste, texture, and overall acceptability. Similarly, sensory evaluations of both cookies and noodle samples were performed by a taste-testing panel, employing a hedonic rating test with ten panelists. They were tasked with evaluating various sensory parameters and assigning scores on a nine-point hedonic scale, where 9 represented 'Like extremely,' 8 for 'Like very much,' and so forth. Preference differences were subsequently evaluated through statistical analysis of the data for variance.

.

2.10 Statistical analysis

The results were expressed as the mean, standard deviation, and coefficient of variation of each species for each parameter was determined. All evaluations of samples were all done in triplicate. Data were statistically analyzed (R statistical software 3.4.1) by one-way analysis of variance (ANOVA). Statistical analysis for the sensory analysis, including variance and Duncan’s Multiple Range Test (DMRT), was performed using the Statistical Analysis System (SAS, 1985) procedures. Mean comparisons were performed using Duncan’s multiple range tests for significant effect at $P < 0.05$.

3. RESULT AND DISCUSSION

3.1 Proximate analysis

3.1.1 Proximate analysis of wheat flour, pumpkin seeds and peels powder

The three kinds of powders were analyzed for proximate analyses which are wheat flour which collected from local market, pumpkin seed powder and pumpkin peel powder. The results showed in the table 01 where results showed significantly higher amounts of moisture, ash, protein, fat, fiber and beta-carotene contents in pumpkin seeds powder and peels powder as compared to marketed wheat flour (control). Similar results were observed for pumpkin seeds powder and peels powder by Ashik et al., 2022; who reported that moisture, ash, protein, fat and fiber content of pumpkin seeds powder was 2.75%, 5.20%, 34.19%, 44.01% and 4.12 %, respectively where as pumpkin peels powder was 10.31%, 6.04%, 12.30 %, 5.77 % and 13.91%, respectively [15].Sometimes the difference in protein and fat value in pumpkin seeds and peels were observed. This might be due to the variation in environment during harvesting such as application of fertilizer (nitrogen content), growing conditions and time and also location of growing areas, oxidation of the fat because most of the fat content in seeds and peels is unsaturated and undergo oxidation easily by atmospheric oxygen. Atmospheric oxidation normally product of alkyl peroxyls of hydrocarbons. Normally when the concentration is low, they can react with themselves which is called self-reaction of alkyl peroxyl radicals. If the concentration is high, in the unsaturated condition the oxygen molecules are added in the subsequent stage, and the self reactions can be lead to aldehydes and alcohols [29]. The gluten content of the wheat flour which collected from local renowned company was 14.47% while pumpkin seeds and peels powder was no gluten, which also we found that the gluten value of Bangladesh around 11% to 15% [31].

Table01: Proximate compositions of powder samples

Powder Samples	Moisture Content (%)	Ash Content (%)	Protein Content (%)	Fat Content (%)	Fiber Content (%)	Beta- carotene (µg/g)	Gluten (%)
Wheat flour	10.60±0.87^{a}	0.57±0.02^{b}	9.62 ±0.01^{b}	1.08±0.03^{b}	0.51±0.09^{b}	14.56±0.34^{b}	14.45±0.76^{a}
Pumpkin seed powder	3.21±0.16^{b}	5.18±0.03^{a}	33.25±0.01^{a}	43.96±0.33^{a}	3.91±0.48^{b}	39.46±0.36^{b}	0
Pumpkin peel powder	10.21±0.13^{a}	4.40±0.03^{a}	15.09±0.30^{ab}	2.94±0.01^{b}	16.83±1.74^{a}	123.55±0.33^{a}	0

All values are express as mean ± SD.
Mean followed by different superscript letters in each column are significantly different ($p<0.05$)

3.1.2 Proximate analysis of pumpkin seed cookies

The proximate compositions of cookies from pumpkin seeds was analyzed for moisture, ash, protein, fat, fiber and beta-carotene which presented in Table 02. Results demonstrated that all pumpkin seed cookies had higher amount of protein, fat, fiber and beta-carotene content as compared to control (100% wheat flour cookie).

Protein content of different cookies varied from 8.09 to 12.03/100 g that was similar to found by Alshehry (2020), reported protein value of in pumpkin seed cookies 12.62 to 13.75% on the variation of the percentage of the pumpkin seeds powder using to develop, Syam et al. (2019) who reported protein value in pumpkin seed cookie 11.20/100 g and also by Dhiman et al. (2018) who reported protein value in cookies as 8.47 to 12.56/100g [16, 17, 32]. Sample S_E had highest protein value (12.03/100g) compared with other recipes. On the contrary, sample S_A which is backed by market wheat flour had the lowest value of protein (8.09/100 g) than other samples. The variations in protein value increase because pumpkin seed contain more protein than market wheat flour.

Fat values were appreciably different among cookies and highest was found in sample S_D (13.95/100g), where lowest content (11.62/100g) were studied in sample S_A. This observation was comparable to that found by Alshehry (2020) who reported fat value in pumpkin seed cookies 7.13/100g [32].The variation in fat value in different cookies may be due to oxidation of the fat because most of the fat content in cookies is unsaturated and undergo oxidation easily by atmospheric oxygen. The ash content of different cookies varied between 0.91/100 to 1.36/100g. This result was consistent to be found by Alshehry (2020) and Syam et al. (2019) who reported ash content in cookies as 1.62/100g and 1.65/100g respectively [17]. Lowest ash value was found in sample S_A which is backed from market wheat flour (0.91/100g) variety while highest value (1.36/100g) was studied in sample S_E which backed from 85% market wheat flour /15% pumpkin seed cookie.

Fiber values were appreciably different among cookies and highest was found in sample S_D (1.61/100g), where lowest content (0.32/100g) were studied in sample S_A. This observation was comparable to that found by Apostol et al. (2020), Alshehry (2020) and Syam et al. (2019) who reported fat value in pumpkin seed cookies 5.74/100g, 6.24/100g and 1.64/100g respectively [17, 32, 37]. Low molecular weight components such as minerals, vitamins and sugars might be lost during washing treatment, leading to a relative increase in the dietary fibre content (Wenberg et al., 2006). The Beta-carotene content of different cookies varied between 4.07 ug/g to 6.36 ug/g. This result was consistent to be found by Dhiman et al. (2018) who reported Beta-carotene

content in cookies as 3.22 to 3.78 mg/100g [16]. Lowest Beta-carotene content was found in sample S_A which is backed from market wheat flour (4.07ug/g) variety while highest value (6.36ug/g) was studied in sample S_E which backed from 80% market wheat flour 20% pumpkin seed cookie.

Table 02: Proximate compositions of pumpkin seeds cookies

Cookie Samples	Moisture content (%)	Ash content (%)	Protein content (%)	Fat content (%)	Fiber content (%)	Beta-carotene (ug/g)
S_A	6.68 ±0.19^{bc}	0.91±0.01^{c}	8.09 ±1.54^{c}	11.62±0.56^{d}	0.32±0.15^{c}	4.07±0.36^{c}
S_B	5.91±0.31^{c}	1.02±0.03^{c}	9.84±0.31^{b}	12.03±0.02^{cd}	0.62±0.19^{bc}	5.18±0.34^{b}
S_C	7.79±0.56^{ab}	1.24±0.01^{ab}	9.84 ±0.31^{b}	12.89±0.01^{bc}	0.92±0.06^{b}	5.27±0.39^{ab}
S_D	7.96± 0.06^{a}	1.15±0.02^{b}	11.15±0.31^{a}	13.95±0.70^{a}	1.61±0.31^{a}	5.77±0.74^{ab}
S_E	7.74±0.54^{ab}	1.36±0.03^{a}	12.03±0.31^{a}	13.78±0.86^{ab}	1.52±0.30^{a}	6.36±1.08^{a}

Values are means of triplicate analysis ± Standard deviation. S_A=Control (100% market wheat flour, Cookie), S_B=95% market wheat flour /5%pumpkin seed cookie, S_C=90% market wheat flour /10%pumpkin seed cookie, S_D=85% market wheat flour /15%pumpkin seed cookie, S_E=80%market wheat flour /20%pumpkin seed cookie)
All values are express as mean ± SD
Mean followed by different superscript letters in each column are significantly different ($p<0.05$)

3.1.3 Proximate compositions of pumpkin peel cookies

The composition of cookies from pumpkin peels was analyzed for moisture, ash, protein, fat, fiber and beta-carotene. The results presented in table 03 where sample P_D contained the highest amount of moisture and sample P_A had the lowest level of moisture. These values were higher to those reported by Mishra and Sharma (2019) who found that moisture content in cookies is 6.59/100g and also by Saleh and Ali (2020) found that moisture content in pumpkin peel cookie was 5.62/100g to 7.09/100g [18, 19].

Protein content of different pumpkin peel cookies varied from 8.09 to 9.04/100g that was similar to found by Saleh and Ali (2020) who reported protein value in pumpkin peel cookie 7.73/100g to 9.42/100g and higher found by Mishra and Sharma (2019) who reported protein value in cookies as 0.08/100g [18]. Sample P_D had highest protein value (9.40/100g) compared with other recipes. On the contrary, sample P_A which is backed by market wheat flour had the lowest value of protein (8.09/100 g) than other samples. The variations in protein value increase because pumpkin peel contains more protein than market wheat flour.

Fat values were appreciably different among cookies and highest was found in sample P_C (11/100g), where lowest content (10.84/100g) were studied in sample P_A. This observation was comparable to that found by Mishra and Sharma (2019) who reported fat value in pumpkin peel

cookies 21.75/100g and also found by Saleh and Ali (2020) who reported fat value in pumpkin peel cookie 14.82/100g to 15.14/100g [18, 19]. The variation in fat value in different cookies may be due to oxidation of the fat because most of the fat content in cookies is unsaturated and undergo oxidation easily by atmospheric oxygen. The ash content of different cookies varied between 0.91/100 to 1.34/100g. This result was consistent to be found by Saleh and Ali (2020) who reported ash content in cookies as 1.30/100g to 2.62/100g [19].Lowest ash value was found in sample P_A which is backed from market wheat flour (0.91/100 g) variety while highest value (1.34/100 g) was studied in sample P_D which backed from 85% market wheat flour /15%pumpkin peel cookie.

Fiber values were appreciably different among cookies and highest was found in sample P_C (5.08/100g), where lowest content (0.32/100g) were studied in sample P_A. This observation was comparable to that found by Saleh and Ali (2020) who reported fiber value in pumpkin peel cookies1.18/100g to 3.17/100 g and higher then found by Mishra and Sharma (2019) who reported fiber value in pumpkin peel cookies 0.16/100g [18, 19].

The Beta-carotene content of different cookies varied between 4.07ug/g to 29.38ug/g. This result was higher to be found by Saleh and Ali (2020) who reported Beta-carotene content in cookies as3.08 to 11.54 mg/100g [19]. Lowest Beta-carotene content was found in sample A which is backed from market wheat flour (4.07ug/g) variety while highest value (39.38ug/g) was studied in sample P_D which backed from 80% market wheat flour /20%pumpkin peel cookie. The variation in beta- carotene value increase because pumpkin peel contain more beta-carotene then market wheat flour.

Table 03: Proximate compositions of pumpkin peel cookies

Cookie Samples	Moisture Content (%)	Ash Content (%)	Protein Content (%)	Fat Content (%)	Fiber Content (%)	Beta-carotene (ug/g)
P_A	6.68 ±0.19^{c}	0.91±0.01^{c}	8.09 ±1.54^{a}	11.62±0.56^{a}	0.32±0.15^{c}	4.07±0.36^{c}
P_B	7.17±0.17^{bc}	1.04±0.01^{b}	8.75±0.61^{a}	10.84±0.16^{b}	2.07±0.19^{bc}	12.40±0.36^{b}
P_C	7.63±0.52^{b}	1.10±0.02^{b}	8.96 ±0.31^{a}	11.00±0.21^{ab}	5.08±0.06^{a}	25.52±0.19^{a}
P_D	9.02± 0.43^{a}	1.34±0.01^{a}	9.40±0.30^{a}	10.95±0.13^{ab}	3.08±0.31^{ab}	29.38±0.37^{a}

Values are means of triplicate analysis ± Standard deviation. P_A=Control (100% wheat, Cookie), P_B=95%wheat/5%pumpkin peel cookie, P_C=90%wheat/10%pumpkin peel cookie, P_D=80%wheat/20%pumpkin peel cookie)
All values are express as mean ± SD.
Mean followed by different superscript letters in each column are significantly different ($p<0.05$)

3.1.4 Proximate compositions of noodles samples:

The composition of noodles from pumpkin seeds and peels powder was analyzed for moisture content, protein, ash and fat content. The results obtained were presented in Table 04. Sample had lower moisture content as compared to all other peels and seeds fortified noodles. Although these values had no significant differences. The protein content of noodles sample ranged from 4.81 to 5.47 %. There were no significant differences in protein content among all noodles sample. All noodles' samples had fat content ranged from 0.59 to 4.96%. Sample N_D (80% wheat/ 10% pumpkin seeds and 10% pumpkin peels noodles) had highest fat content which is slightly higher than control. The ash content of noodles sample ranged from 1.60 to 2.2 7%. Sample N_D (80%wheat/10%pumpkin seeds and 10% pumpkin peels noodles) sample had highest ash content. There was significantly different in ash content was observed for sample N_D. This might be due to the higher amount of mineral content present in the seeds and peels samples. The fiber content of noodles sample ranged from 1.49 to 8.88%. Control (wheat flour noodles) sample had lower fiber content as compared to all other peels and seeds fortified noodles.

Table04: Proximate compositions of noodles samples.

Noodles Samples	Moisture Content(%)	AshContent (%)	Protein Content (%)	Fat Content (%)	Fiber Content (%)	Beta-carotene (ug/g)
N_A	8.94±0.62^a	1.60±0.026^b	5.47 ±0.31^a	4.30±0.47^a	1.49 ±0.23^a	0.324 ±0.003^a
N_B	9.67±0.97^a	1.85±0.20^b	5.03±0.30^a	0.59 ±0.01^a	6.36 ±1.58^a	0.315 ±0.004^a
N_C	9.44±0.85^a	1.72±0.034^b	4.81±0.02^a	4.96 ±0.44^a	8.88±1.13^a	0.222±0.004^b
N_D	9.23±0.42^a	2.27±0.140^a	5.25 ±0.03^a	0.59± 0.07^a	8.82 ± 0.19^a	0.318 ± 0.002^a

Values are means of triplicate analysis ± Standard deviation. N_A=Control (100% wheat flour noodles), N_B=90%wheat/10% pumpkin seeds noodles, N_C=90%wheat/10% pumpkin peels noodles, N_D=80%wheat/10%pumpkin seeds and 10% pumpkin peels noodles
All values are express as mean ± SD.
Mean followed by different superscript letters in each column are significantly different ($p<0.05$)

3.1.5 β-Carotene determination for noodles

Almeida et al. (2011) reported that, foods which are rich in antioxidants play an essential role in the prevention of carcinogenic diseases. The antioxidant capacities of samples such as pumpkin peels and seeds may vary depending on their contents of vitamin C, vitamin E, carotenoids, and particularly β-carotene [20], and lycopene [21] as well as other pigments such as flavonoids and other polyphenols [22]. The results for β-carotene content of the noodles are listed on Table 04. Carotenoids are tetraterpenoids found throughout not only the flowering plant kingdom but also in vegetables and fruits as a pigment mostly responsible for various color such as red, orange or yellow color in peels, seeds, flash leaves and fruits which are important as vitamin A precursors.

As they are found widely in all kinds' plants and plant parts, it is not surprising that a large number of carotenoids have been reported in the pumpkin seeds and peels powder [23]. Noodles from pumpkin seeds powder showed the highest ($P < 0.05$) content of β-carotene except control. Noodles manufactured from peels powder and mixture of peels and seeds have also shown to be an excellent source of β-carotene. Similar levels of β-carotene have been previously reported for pumpkin flesh powder [24]. However, these pumpkin peels and seeds powder should not be considered as a rich source of carotenoids, where values as high as 161mg/100 gd.b. for wine palm (*Mauritiavinifera*)one of the most important vitamin A precursors has been represented [25, 26].

3.2 Cooking properties (Cooking yield)

Generally speaking, high cooking yield gains and low cooking loss would be desirable characteristics for high quality of any kinds of noodles. Flour noodles with added different types of pumpkin powder such as peels powder, seeds powder and mixture of peels and seeds powders had lower cooking yield gains than the flour noodle samples with no added pumpkin (Table 05).

It was found that control sample (N_A) contained the highest amount of cooking yield and 10% seeds powder noodles sample (N_B) had the lowest level of cooking yield while 10% pumpkin seeds powder has lowest cooking loss and the mix powder has highest cooking loss. Including controls all noodles' samples cooking loss is considerable higher then Apostol et al. (2020) & Mestres et al. (1988) [14]. But all noodles' samples cooking yield is much higher [24].

The parameters of cooked noodles varied considerably for gumminess and hardness. However, springiness, cohesiveness, and chewiness were not different among the samples, indicating the pumpkin powder did not affect such parameters [24, 27].

Table 05: Cooking properties of noodles

Sample	Noodles cooking Yield (%)	Noodles cooking Loss (%)
N_A	275.254±0.10^a	3.723±0.010^b
N_B	256.368±0.10^b	3.421 ±0.010^b
N_C	250.885±0.10^b	4.217±0.010^{ab}
N_D	260.089±0.01^b	5.007±0.002^a

Values are means of triplicate analysis ± Standard deviation. N_A=Control (100% wheat flour noodles), N_B=90%wheat/10% pumpkin seeds noodles, N_C=90%wheat/10% pumpkin peels noodles, N_D=80%wheat/10%pumpkin seeds and 10% pumpkin peels noodles

All values are express as mean±SD.

Mean followed by different superscript letters in each column are significantly different ($p<0.05$)

3.3 Hunter Color Values

Hunter color values were represented by L*, a*, b*, and ΔE where L* is a measure of lightness, a* redness, b* yellowness and ΔE color differences. The Hunter color parameters L*, a*, b*, and ΔE have been widely used to evaluate color changes during dehydration of fruits and vegetable products. There are three types of powdered samples such as wheat flour which collected from local market served as control, Pumpkin seed powder and Pumpkin peel powder. The results of Hunter Color Values (L*,a*,b*,ΔE) of pumpkin seeds and peels powder are represented in table 06, pumpkin seed cookies are shown in Table 07 & pumpkin peels cookies are shown in Table 08.

Results indicates lightly decrease in L*, b* and ΔE value while increase in a* value of all cookies samples prepared from both seeds and peels powders with the level of increasing sample concentrations. The increase of hunter a* values (redness) with increase of sample concentration for all cookie samples might be due to the influence of beta-carotene concentration. The flour colors and cookies color can best be described by the change in total color difference, ΔE values, where locally collected wheat flour has a significance difference with both pumpkin seeds and peels powder. Pumpkin seeds cookies S_A, S_E and pumpkin peels cookies P_A, P_B and P_c shows almost similar results The lower ΔE values may be due to the loss of phenolics and oxidation during drying, boiling treatment and oven temperature [28].

Table 06: Hunter Color Values (L*, a*, b*,ΔE) of pumpkin seeds and peels powder samples

Powder Samples	L*	a*	b*	ΔE
Wheat Flour	80.53±2.87^{a}	1.82±0.19^{a}	10.57±0.55^{b}	81.24±2.92^{a}
Pumpkin seed powder	52.79±1.24^{b}	3.57±0.74^{a}	19.46±1.35^{a}	56.39±1.77^{b}
Pumpkin peel powder	65.09±4.74^{ab}	1.34±0.26^{a}	18.72±4.27^{a}	67.78±5.73^{ab}

All values are express as mean ± SD.
Mean followed by different superscript letters in each column are significantly different ($p<0.05$).

Table 07: Hunter Color Values (L*, a*,b*,ΔE) of pumpkin seeds cookies

Cookie Samples	L*	a*	b*	ΔE
S_A	50.94±1.85^{ab}	16.20±0.80^{a}	28.46±0.45^{a}	60.56±1.98^{ab}
S_B	49.14±1.90^{ab}	13.51±0.96^{b}	26.88±0.41^{ab}	57.621±2.04^{ab}
S_C	44.04±2.93^{b}	14.40±1.45^{ab}	23.40±2.43^{b}	51.91±3.98^{b}
S_D	43.02±0.41^{b}	14.46±0.48^{ab}	24.50±0.82^{ab}	51.58±.87^{b}
S_E	54.07±1.67^{a}	13.95±1.18^{ab}	27.60±0.13^{ab}	62.29±1.77^{a}

Values are means of triplicate analysis ± Standard deviation. S_A=Control (100% market wheat flour, Cookie), S_B=95% market wheat flour /5%pumpkin seed cookie, S_C=90% market wheat flour /10%pumpkin seed cookie, S_D=85% market wheat flour /15%pumpkin seed cookie, S_E=80%market wheat flour /20%pumpkin seed cookie)
All values are express as mean ± SD
Mean followed by different superscript letters in each column are significantly different ($p<0.05$)

Table 08: Hunter Color Values (L*, a*,b*,ΔE) of pumpkin peels cookies

Cookie Samples	L*	a*	b*	ΔE
P_A	50.94±1.85^{a}	16.20±0.80^{a}	28.46±0.45^{a}	60.56±1.98^{a}
P_B	55.84±1.60^{a}	10.67±0.19^{b}	28.97±0.24^{a}	63.81±1.54^{a}
P_C	51.19±1.13^{a}	9.70±0.72^{b}	29.48±0.86^{a}	59.86±1.51^{a}
P_D	41.50±2.34^{b}	13.25±0.61^{ab}	24.08±2.27^{b}	49.78±3.21^{b}

Values are means of triplicate analysis ± Standard deviation. P_A=Control (100% wheat, Cookie), P_B=95%wheat/5%pumpkin peel cookie, P_C=90%wheat/10%pumpkin peel cookie, P_D=80%wheat/20%pumpkin peel cookie)
All values are express as mean ± SD.
Mean followed by different superscript letters in each column are significantly different ($p<0.05$)

3.4 Antioxidant Properties

3.4.1 Total Phenolic Compound and Antioxidant Properties (DPPH scavenging activity and FRAP Ferric reducing power assay) of powder samples

The total phenolic compound, DPPH and FRAP antioxidant properties of market wheat flour, pumpkin seed powder and pumpkin peel powder are shown in Table 09. The highest total phenolic compound (268.38mg/100g) was found in pumpkin seed powder sample. It is commonly known as seeds are an excellent source of nutritional compositions and other bioactive compound specially phenols but pumpkin peels also very excellent dource of antioxidant which also supported by the Hagos et al. 2023 where Hagos et al. 2023 reported the phenolic compound are almost four times higher the pumpkin seeds in pumpkin peels [32]. The variety of growth circumstances, plant parts, processing, analytical methods and environment,

could all be considered in explaining the considerable variances in the phenolic contents [32].

Table 09: Total phenolic compound and antioxidant properties (DPPH and FRAP) of market wheat flour, pumpkin seed powder and pumpkin peel powder sample

Powder Samples	Total Phenolic Compound (mg/100g)	DPPH scavenging activity (m mole/100g)	FRAP Ferric reducing power assay (m mole/100g)
Wheat Flour	83.62±2.85^{b}	122.23±6.86^{c}	279.75±37.12^{c}
Pumpkin seed powder	268.38±1.02^{a}	160.17±14.55^{b}	688.50±10.60^{b}
Pumpkin peel powder	87.82±1.03^{b}	217.52±27.76^{a}	1374.75±15.90^{a}

All values are express as mean ± SD.
Mean followed by different superscript letters in each column are significantly different ($p<0.05$)

3.4.2 Total Phenolic Compound and Antioxidant Properties (DPPH scavenging activity and FRAP Ferric reducing power assay) of pumpkin seeds cookies

Table 10 shows the total phenolic compound and antioxidant properties (DPPH and FRAP) of pumpkin seeds cookies. All pumpkin seeds cookies comparatively showed higher total phenolic compound as compared to control (market wheat flour). This is because of higher total phnolic compound present in pumpkin seeds samples. As a result, pumpkin seeds powders fortification in wheat flour cookies can be excellent substitute of wheat flour to enrich nutrients. It was observed that pumpkin seeds cookies had higher DPPH scavenging activity as compared to control (market wheat flour). On the other hand, FRAP antioxidant properties of pumpkin seeds cookies samples ranged from 1157.25 to 2079.75 m mole/100g. It was also evaluated that pumpkin seeds cookies had higher FRAP antioxidant properties as compared to control (market wheat flour). The higher values of DPPH and FRAP antioxidant activity of pumpkin seeds fortified cookies samples might be due to the presence of higher phenolic compounds in pumpkin seeds.

Table 10: Total phenolic compound and antioxidant properties (DPPH and FRAP) of pumpkin seeds cookies

Cookie Samples	Total Phenolic Compound (mg/100g)	DPPH scavenging activity (m mole/100g)	FRAP Ferric reducing power assay (m mole/100g)
S_A	24.51^{a}±0.57	162.52^{ab}±12.47	1187.25^{b}±58.33
S_B	82.57^{a}±0.01	117.97^{b}±18.09	1157.25^{b}±26.51
S_C	76.37^{a}±0.22	196.35^{ab}±39.09	1269.75^{b}±47.72
S_D	71.75^{a}±2.74	286.64^{a}±22.46	2079.75^{a}±79.54
S_E	60.28^{a}±1.14	179.00^{ab}±5.40	1873.50^{a}±265.1

Values are means of triplicate analysis ± Standard deviation. S_A=Control (100% market wheat flour, Cookie), S_B=95% market wheat flour /5%pumpkin seed cookie, S_C=90% market wheat flour /10%pumpkin seed cookie, S_D=85% market wheat flour /15%pumpkin seed cookie, S_E=80%market wheat flour /20%pumpkin seed cookie)
All values are express as mean ± SD
Mean followed by different superscript letters in each column are significantly different ($p<0.05$)

3.4.3 Total Phenolic Compound and Antioxidant Properties (DPPH scavenging activity and FRAP Ferric reducing power assay) of pumpkin peels cookies

Table11 represents the total phenolic compound and antioxidant properties (DPPH and FRAP) of pumpkin peels cookies. All pumpkin peels cookies showed higher total phenolic compound as compared to control (market wheat flour). This is because of higher total phenolic compound present in pumpkin peels samples. DPPH scavenging activity of pumpkin peels cookies samples ranged from 111.50 to 199.29 m mole/100g.The highest amount of DPPH properties (199.29 **m mole/100g**) was observed for the cookie sample containing 20% peel powder (sample D). Ferric reducing power (FRAP) assay was evaluated for pumpkin peels cookies which was ranged from 1029.75 to 1187.25 m mole/100g. It was evaluated that the market wheat flour (control) had the highest (1187.25 mmole/100g) FRAP value.

Table 11: Total phenolic compound and antioxidant properties (DPPH and FRAP) of pumpkin peels cookies

Cookie Samples	Total Phenolic Compound (mg/100g)	DPPH scavenging activity (m mole/100g)	FRAP Ferric reducing power assay (mmole/100g)
P_A	24.51^{b}±0.57	162.52^{a}±12.47	1187.25^{b}±58.33
P_B	80.47^{a}±2.51	138.55^{a}±13.51	1048.50^{a}±63.63
P_C	46.48^{ab}±1.02	111.50^{a}±7.27	1063.50^{a}±30.75
P_D	60.45^{ab}±5.02	199.294^{a}±25.37	1029.75^{a}±5.30

Values are means of triplicate analysis ± Standard deviation. P_A=Control (100% wheat, Cookie), P_B=95%wheat/5%pumpkin peel cookie, P_C=90%wheat/10%pumpkin peel cookie, P_D=80%wheat/20%pumpkin peel cookie)
All values are express as mean ± SD.
Mean followed by different superscript letters in each column are significantly different ($p<0.05$)

3.4.4 Total Phenolic Compound and Antioxidant Properties (DPPH) of noodles samples

Table 12 displayed total phenolic compound of all noodles sample ranged from 9.55 to 34.96 mg/100g. Sample N_B had the highest total phenolic compound (34.96mg/100g). On the other hand, DPPH scavenging activity of all noodles sample ranged from 14.03 to 27.08 m mole/100g. Sample N_C had the highest DPPH scavenging activity (27.08 m mole/100g). All noodles samples had higher DPPH scavenging activity than control. It indicated that wheat flour can be fortified with pumpkin seeds and peels powders to enrich nutritional properties specially antioxidant activities.

Table 12: Total Phenolic Compound and Antioxidant Properties (DPPH) **of noodles samples.**

Noodles Samples	Total Phenolic Compound (mg/100g)	DPPH scavenging activity (m mole/100g)
N_A	18.86±0.49^{ab}	14.03±2.54^{b}
N_B	34.96±0.27^{a}	16.28±1.79^{b}
N_C	21.82±1.09^{ab}	27.08±1.27^{a}
N_D	9.55±1.09^{b}	18.75±1.42^{ab}

Values are means of triplicate analysis ± Standard deviation. N_A=Control (100% wheat flour noodles), N_B=90%wheat/10% pumpkin seeds noodles, N_C=90%wheat/10% pumpkin peels noodles, N_D=80%wheat/10%pumpkin seeds and 10% pumpkin peels noodles
All values are express as mean ± SD.
Mean followed by different superscript letters in each column are significantly different ($p<0.05$)

3.5 Hydration properties of powder samples:

Hydration properties such as water absorption index, water solubility index and swelling capacity of market wheat flour, pumpkin seed powder and pumpkin peel powder are shown in Table 13. The values of all powder samples ranged from 187.40 to 764.60 g/100g, 5.60 to 17.60 ml/100g and 5.93 to 21.37ml/100g for water absorption index, water solubility index and swelling capacity, respectively. Hussain et al. (2023) reported 60.31/100g and 60.98/100g water absorption index of the pumkin peels powder and pumpkin seeds powder [38]. The data showed pumpkin peels powder and seeds powder had hydration properties which is close to the market wheat flour (control) sample. It indicates that these two-powder ingredients (seeds powder and peels powder) can be partially replaced by wheat flour for making less gluten cookie. It is impossible completely reduced the gluten by the seeds and peels powder but we can enrich the noodles nutrition value by partially adding the waste materials of pumpkin.

Table 13: Hydration Properties (water absorption index, water solubility index and swelling capacity of powder samples)

Cookie Samples	Water Absorption Index (g/100g)	Water Solubility Index (ml/100g)	Swelling Capacity (ml/100g)
Wheat Flour	764.60±20.64^{a}	17.60±1.13^{a}	21.37±1.66^{a}
Pumpkin seed powder	240.40±6.70^{b}	11.20±0.56^{b}	9.53±0.71^{b}
Pumpkin peel powder	187.40±0.20^{b}	5.60±0.01^{c}	5.93±0.01^{c}

All values are express as mean ± SD.
Mean followed by different superscript letters in each column are significantly different (p<0.05)

3.6 Sensory evaluation

Ten judges' evaluated cookies and noodles made with different ratios of pumpkin powder based on texture, aroma, color, taste, and overall acceptability. Tables 13, 14, and 15 present mean scores for each criterion. Two-way ANOVA for color preference indicated significant differences ($p<0.05$) among the samples. However, DMRT showed no significant taste differences in pumpkin peel cookies. Among peel cookies, sample P_C scored highest in color, aroma, texture, and overall acceptability. In pumpkin seed cookies, sample S_D with 15% powder achieved the highest scores. For noodles, sample P_C with 10% pumpkin peel powder outperformed others, securing the highest scores in color, texture, aroma, taste, and overall

acceptability. The findings suggest optimal ratios for enhancing sensory attributes in pumpkin-enriched cookies and noodles.

The sensory characteristics of cookies containing varying levels of pumpkin seeds are detailed in Table 15. The results of sensory evaluation reveal significant variations among different cookie products. Notably, Sample PC (90% wheat/10% pumpkin peel cookie) achieved the highest ratings in color, aroma, taste, texture, and overall acceptability, scoring 8, 7.33, 7.67, 8, and 8.33, respectively, as illustrated in Table 14. Similarly, Sample SD (85% market wheat flour/15% pumpkin seed cookie) demonstrated superior attributes, scoring highest in color, aroma, taste, texture, and overall acceptability, with ratings of 7.50, 7.67, 8, 7.67, and 8.33, respectively, as shown in Table 15. Additionally, in Table 16, NC (90% wheat/10% pumpkin peels noodles) emerged with the highest score among others.

The enhancement in texture upon increasing pumpkin seeds in cookies may be attributed to their high protein content, as observed by Atuonwu and Akobundu (2010). The color score ranged from 6.33 in control cookies to 7.50 in cookies containing 20% pumpkin seeds, reflecting the desirable effect of pumpkin seeds' high protein content. Furthermore, the gradual decrease in the color score of cookies may be attributed to sugar caramelization and Maillard reactions between sugars and amino acids, consistent with findings by Alshehry et al. (2020) indicating an improvement in color score with higher supplementation levels [33, 34].

Cookies exhibited significant variations in flavor, with the highest score observed in those containing 15% pumpkin seeds and the lowest in control cookies. This increase in flavor was also noted by Atuonwu and Akobundu (2010) with higher levels of pumpkin seeds. Moreover, the highest taste score was found in products containing 15% pumpkin seeds, emphasizing the importance of pumpkin seed inclusion for developing taste characteristics in cookies (Murkovic, 2004) [33, 36].

Studies by Alshehry (2020) and Kanwal et al. (2015) demonstrated successful incorporation of pumpkin seed flour as a partial replacement for wheat flour, resulting in cookies with enhanced nutritional value and wholesomeness without compromising overall acceptability [34, 35].

Table 14: Mean score for texture, aroma, color, taste and overall acceptability of Pumpkin peel cookies.

Cookie's type	Color	Aroma	Taste	Texture	Overall acceptability
P_A	7.67± 0.99[a]	7.00±0 .85[ab]	7.00±0. 86[a]	7.33±0.98[a]	7.33±1.31[b]
P_B	7.67± 0.49[a]	6.58±0 .51[bc]	6.91±0. 80[a]	7.33±1.30[a]	7.66±0.98[ab]
P_C	8.00± 0.86[a]	7.33±0 .49[a]	7.67±0. 99[a]	8.00±0.85[a]	8.330.49±[a]
P_D	6.67± 0.99[b]	6.33±0 .50[c]	7.17±0. 84[a]	6.33±0.50[b]	6.00±0.01[c]

Values are means of triplicate analysis ± Standard deviation. P_A=Control (100% wheat, Cookie), P_B=95%wheat/5%pumpkin peel cookie, P_C=90%wheat/10%pumpkin peel cookie, P_D=80%wheat/20%pumpkin peel cookie)
All values are express as mean ± SD.
Mean followed by different superscript letters in each column are significantly different ($p<0.05$)

Table 15: Mean score for texture, aroma, color, taste and overall acceptability of Pumpkin seed cookies.

Cookie's type	Color	Aroma	Taste	Texture	Overall acceptability
S_A	6.42± 0.51[b]	7.33±1 .30[ab]	7.00± 0.85[b]	7.33±0.98[a]	7.33±1.30[b]
S_B	6.58± 0.50[b]	6.67±0 .49[b]	6.33± 0.98[b]	6.33±0.49[b]	7.33±1.30[b]
S_C	6.33± 0.77[b]	6.67±0 .98[b]	6.67± 0.49[b]	7.00±0.85[ab]	7.00±0.85[b]
S_D	7.50± 0.67[a]	7.67±0 .98[a]	8.00± 0.85[a]	7.67±0.78[a]	8.33±0.49[a]
S_E	6.58± 0.51[b]	7.00±0 .96[ab]	6.67± 0.50[b]	7.00±0.85[ab]	7.33±1.32[b]

Values are means of triplicate analysis ± Standard deviation. S_A=Control (100% market wheat flour, Cookie), S_B=95% market wheat flour /5%pumpkin seed cookie, S_C=90% market wheat flour /10%pumpkin seed cookie, S_D=85% market wheat flour /15%pumpkin seed cookie, S_E=80%market wheat flour /20%pumpkin seed cookie)
All values are express as mean ± SD
Mean followed by different superscript letters in each column are significantly different ($p<0.05$)

Table 16 Mean score for texture, aroma, color, taste and overall acceptability of Pumpkin seed and peel noodles.

Noodles type	Color	Aroma	Taste	Texture	Overall acceptability
N_A	7.67± 0.98^a	7.25±1 .21^a	7.00± 0.84^a	7.33±0. 98^a	7.33± 1.31^{bc}
N_B	7.67± 0.49^a	7.33±0 .50^a	7.00± 0.95^a	7.33±1. 30^a	7.67± 0.99^{ab}
N_C	8.00± 0.85^a	7.67±0 .65^a	7.67± 0.99^a	8.00±0. 85^a	8.33± 0.50^a
N_D	6.67± 0.98^b	6.33±0 .49^b	6.00± 0.01^b	6.33±0. 49^b	6.58± 0.58^c

Values are means of triplicate analysis ± Standard deviation. N_A=Control (100% wheat flour noodles), N_B=90%wheat/10% pumpkin seeds noodles, N_C=90%wheat/10% pumpkin peels noodles, N_D=80%wheat/10%pumpkin seeds and 10% pumpkin peels noodles
All values are express as mean ± SD.
Mean followed by different superscript letters in each column are significantly different ($p<0.05$)

4. Conclusions

Enriching confectionery products with pumpkin seeds and peels enhances their nutritional value. This process also ensures that the products have satisfactory technological and sensory characteristics. The effects of seeds and peels on the physicochemical and nutritional characteristics of different confectionary products such as cookies and noodles were investigated. Pumpkin seed and peel powders could be used not only enrich nutritious value but also to enhance the quality of food products antioxidant properties such as total phenolic compounds where all pumpkin peels sample showed the enrichment of the confectionary products. The increase in fibre and ash content in the cookies and noodles showed qualitative and quantitative advantages when compared to the market flour. The sensory acceptability rate reached 78%, which refers to "Good" in the hedonic rating scale, which is considered good acceptability. The results demonstrate production viability for this alternative food due to its very low cost and easy to preparation but more importantly, it can be destined to different meals because it can improve the nutritional life.

ACKNOWLEDGEMENT

This study was supported by the Ministry of Science and Technology, Government of the Peoples' Republic of Bangladesh Special Allocation (Grant number ES 382; 2020-2021).

REFERENCES

[1] Norfezah, M.N., Hardacre, A. and Brennan, C.S., 2011. Comparison of waste pumpkin material and its potential use in extruded snack foods. Food Science and Technology International, 17(4), pp.367-373.

[2] Statistics, B.B.O., 2021. Statistical yearbook of Bangladesh. Statistics Division, Ministry of Planning, Dhaka, Government of the People’s Republic of Bangladesh.

[3] El-Adawy, T.A. and Taha, K.M., 2001. Characteristics and composition of watermelon, pumpkin, and paprika seed oils and flours. Journal of agricultural and food chemistry, 49(3), pp.1253-1259.

[4] Brennan, C.S. and Samyue, E., 2004. Evaluation of starch degradation and textural characteristics of dietary fiber enriched biscuits. International Journal of Food Properties, 7(3), pp.647-657.

[5] Fagan, C.C., O’Donnell, C.P., Cullen, P.J. and Brennan, C.S., 2006. The effect of dietary fibre inclusion on milk coagulation kinetics. Journal of Food Engineering, 77(2), pp.261-268.

[6] Tudorica, C.M., Jones, T.E.R., Kuri, V. and Brennan, C.S., 2004. The effects of refined barley β- glucan on the physico- structural properties of low- fat dairy products: curd yield, microstructure, texture and rheology. Journal of the Science of Food and Agriculture, 84(10), pp.1159-1169.

[7] Malkanthi, H.H.A. and Umadevi, S.H., 2018. Effect of dried pumpkin pulp and seed powder on physical, chemical and sensory properties of biscuits. Int J Sci Res, 7, pp.32-4.

[8] Kim, M.Y., Kim, E.J., Kim, Y.N., Choi, C. and Lee, B.H., 2012. Comparison of the chemical compositions and nutritive values of various pumpkin (Cucurbitaceae) species and parts. Nutrition research and practice, 6(1), pp.21-27.

[9] Adams, G.G., Imran, S., Wang, S., Mohammad, A., Kok, S., Gray, D.A., Channell, G.A., Morris, G.A. and Harding, S.E., 2011. The hypoglycaemic effect of pumpkins as anti-diabetic and functional medicines. Food Research International, 44(4), pp.862-867.

[10] Yadav, M., Jain, S., Tomar, R., Prasad, G.B.K.S. and Yadav, H., 2010. Medicinal and biological potential of pumpkin: an updated review. Nutrition research reviews, 23(2), pp.184-190.

[11] AOAC (1998). Official Methods of Analysis of AOAC International (14^{th}ed.). Washington, DC, USA: Association of Official AnalyticalChemistry. Methods, 950.46, 938.08, 960.39, 955.04

[12] Navacchi, M.F.P., de Carvalho, J.C.M., Takeuchi, K.P. and Danesi, E.D.G., 2012. Development of cassava cake enriched with its own bran and Spirulinaplatensis. ActaScientiarum.Technology, 34(4), pp.465-472.

[13] Akter, M.S., Ahmed, M. and Eun, J.B., 2010. Solvent effects on antioxidant properties of persimmon (Diospyros kaki L. cv. Daebong) seeds. International journal of food science & technology, 45(11), pp.2258-2264.

[14] Mestres, C., Colonna, P. and Buléon, A., 1988. Characteristics of starch networks within rice flour noodles and mungbean starch vermicelli. Journal of food science, 53(6), pp.1809-1812.

[15] Hussain, A., Kausar, T., Sehar, S., Sarwar, A., Ashraf, A.H., Jamil, M.A., Noreen, S., Rafique, A., Iftikhar, K., Quddoos, M.Y. and Aslam, J., 2022. A Comprehensive review of functional ingredients, especially bioactive compounds present in pumpkin peel, flesh and seeds, and their health benefits. Food Chemistry Advances, p.100067.

[16] Dhiman, A.K., Bavita, K., Attri, S. and Ramachandran, P., 2018. Preparation of pumpkin powder and pumpkin seed kernel powder for supplementation in weaning mix and cookies. International Journal of Chemical Studies, 6(5), pp.167-175.

[17] Syam, A., Zaenal, Y.K., Aulia, N.A., Wati, I.P. and Mansur, M.A., 2019. Development and biochemical analysis of pumpkin seed (CucurbitamoschataDurch) biscuits. Pak J Nutr, 18(8), pp.743-6.

[18] Mishra, S. and Sharma, K., 2019. Development of pumpkin peel cookies and its nutritional composition. Journal of Pharmacognosy and Phytochemistry, 8(4), pp.370-372.

[19] Saleh, S.M. and Ali, S., 2020. Physico-functional properties, nutritional quality, and sensory characteristics of pumpkin peel puree fortified biscuit. Egyptian Journal of Food Science, 48(2), pp.203-212.

[20] Von Lintig, J., 2010. Colors with functions: elucidating the biochemical and molecular basis of carotenoid metabolism. Annual review of nutrition, 30, pp.35-56.

[21] Shami, N.J.I.E. and Moreira, E.A.M., 2004. Lycopene as an antioxidant agent. Revista de nutrição, 17(2), pp.227-236.

[22] Saura-Calixto, F. and Goñi, I., 2006. Antioxidant capacity of the Spanish Mediterranean diet. Food chemistry, 94(3), pp.442-447.

[23] Pierson, J.T., Dietzgen, R.G., Shaw, P.N., Roberts- Thomson, S.J., Monteith, G.R. and Gidley, M.J., 2012. Major Australian tropical fruits biodiversity: Bioactive compounds and their bioactivities. Molecular nutrition & food research, 56(3), pp.357-387.

[24] Lee, C.H., Cho, J.K., Lee, S.J., Koh, W., Park, W. and Kim, C.H., 2002. Enhancing β- carotene content in Asian noodles by adding pumpkin powder. Cereal chemistry, 79(4), pp.593-595.

[25] Godoy, H.T. and Rodriguez-Amaya, D.B., 1998. Occurrence of cis isomers of provitaminsA in Brazilian vegetables. Journal of Agricultural and Food Chemistry, 46(8), pp.3081-3086.

[26] Maria do Socorro, M.R., Alves, R.E., de Brito, E.S., Pérez-Jiménez, J., Saura-Calixto, F. and Mancini-Filho, J., 2010. Bioactive compounds and antioxidant capacities of 18 non-traditional tropical fruits from Brazil. Food chemistry, 121(4), pp.996-1002.

[27] Park, W., Shelton, D.R., Peterson, C.J., Wehling, R.L. and Kachman, S.D., 1997. Evaluation of Korean raw noodle (SaengMyon) color and cooking properties among hard red winter and hard white wheat samples. Food Science and Biotechnology, 6(1), pp.20-25.

[28] Yap, E.S.P., Uthairatanakij, A., Laohakunjit, N. and Jitareerat, P., 2022. Influence of hot air drying on capsaicinoids, phenolics, flavonoids and antioxidant activities of 'Super Hot'chilies. PeerJ, 10, p.e13423.

[29] Ghigo, G., Maranzana, A., Causà, M. and Tonachini, G., 2007. Theoretical mechanistic studies on oxidation reactions of some saturated and unsaturated organic molecules. Theoretical Chemistry Accounts, 117, pp.699-707.

[30] Taneya, M.L.J., Biswas, M.M.H. and Shams-Ud-Din, M., 2014. The studies on the preparation of instant noodles from wheat flour supplementing withs weet potato flour. Journal of the Bangladesh Agricultural University, 12(1), pp.135-142.

[31] Saeid, A., Hoque, S., Kumar, U., Das, M., Muhammad, N., Rahman, M.M. and Ahmed, M., 2015. Comparative studies on nutritional quality of commercial wheat flour in Bangladesh. Bangladesh Journal of Scientific and Industrial Research, 50(3), pp.181-188.

[32] Hagos, M., Chandravanshi, B.S., Redi-Abshiro, M. and Yaya, E.E., 2023. Determination of total phenolic, total flavonoid, ascorbic acid contents and antioxidant activity of pumpkin flesh, peel and seeds. Bulletin of the Chemical Society of Ethiopia, 37(5), pp.1093-1108.

[33] Atuonwu AC, Akobundu A., 2010. Nutritional and Sensory Guality of Cookies Supplemented with Defatted Pumpkin (Cucurbita pepo) Seed Flour. Pakistan Journal of Nutrition, 9(7), pp.672-677.

[34] Alshehry, G.A., 2020. Preparation and nutritional properties of cookies from the partial replacement of wheat flour using pumpkin seeds powder. World, 9(2), pp.48-56.

[35] Kanwal, S., Raza, S., Naseem, K., Amjad, M., Bibi, N. and Gillani, M., 2015. Development, physico-chemical and sensory properties of biscuits supplemented with pumpkin seeds to combat childhood malnutrition in Pakistan. Pakistan Journal of Agricultural Research, 28(4).

[36] Murkovic, M., Piironen, V., Lampi, A.M., Kraushofer, T. and Sontag, G., 2004. Changes in chemical composition of pumpkin seeds during the roasting process for production of pumpkin seed oil (Part 1: non-volatile compounds). Food chemistry, 84(3), pp.359-365.

[37] Apostol, L., Moșoiu, C.L.A.U.D.I.A., Iorga, C.S. and Martínez, S.Á., 2020. Effect of the addition of pumpkin powder on the physicochemical qualities and rheological properties of wheat flour. Romanian Biotechnological Letters, 25(3), pp.1594-1600.

[38] Hussain, A., Kausar, T., Aslam, J., Quddoos, M.Y., Ali, A., Kauser, S., Zerlasht, M., Rafique, A., Noreen, S., Iftikhar, K. and Waheed Iqbal, M., 2023. Physical and rheological studies of biscuits developed with different replacement levels of pumpkin (Cucurbita Maxima) Peel, flesh, and seed powders. Journal of Food Quality, 2023.

[39] Stevenson, D.G., Eller, F.J., Wang, L., Jane, J.L., Wang, T. and Inglett, G.E., 2007. Oil and tocopherol content and composition of pumpkin seed oil in 12 cultivars. Journal of agricultural and food chemistry, 55(10), pp.4005-4013.

[40] Al Zuhair, H.A.N.A., Abd El-Fattah, A.A. and El-Sayed, M.I., 2000. Pumpkin-seed oil modulates the effect of felodipine and captopril in spontaneously hypertensive rats. Pharmacological Research, 41(5), pp.555-563.

[41] Jian, L., Du, C.J., Lee, A.H. and Binns, C.W., 2005. Do dietary lycopene and other carotenoids protect against prostate cancer?. International journal of cancer, 113(6), pp.1010-1014.

Click here to access/download
Supporting Information
Supporting Information files - Tables.docx

Click here to access/download
Supporting Information
Supporting Information files - Table 14 Data.xlsx

Click here to access/download
Supporting Information
Supporting Information files - Table 15 Data.xlsx

Click here to access/download
Supporting Information
Supporting Information files - Table 16 Data.xlsx

Click here to access/download
Supporting Information
Supporting Information files - Photo.docx

Title: "Enhancing Nutritional Value and Quality of Cookies through Pumpkin Peel and Seed Powder Fortification~~: A Wheat Flour Replacement Approach~~"

Author List:

1) Md. Asaduzzaman
   Assistant Professor,
   Department of Food Processing and Preservation, Hajee Mohammad Danesh Science and Technology University, Dinajpur, Bangladesh
   Department of Food Engineering and Technology, State University of Bangladesh, Dhaka, Bangladesh.
   Email: bitatipu@gmail.com; mzaman@sub.edu.bd

2) Mahbubur Rahman
   Department of Food Engineering and Technology, State University of Bangladesh, Dhaka, Bangladesh.
   Email:rmahbub896@gmail.com

3) Mst. Sorifa Akter*
   Assistant Professor,
   Department of nutrition and food Engineering, Daffodil International University, Dhaka, Bangladesh.
   Email: sorifa.nfe@diu.edu.bd

* Corresponding Author
Corresponding Author email address: sorifa.nfe@diu.edu.bd

Abstract

Background: Recently, the pumpkin by-products such as peels and seeds become more and more interesting for confectionary sector, because of the nutritional and bioactive composition. The study was conducted to develop cookies and noodles using different pumpkin by-products (such as peels and seeds) powders in different concentrations and to evaluate the changes in quality characteristics. The powders were prepared from pumpkin seeds and peels.

Methodology: In this study, various concentrations of pumpkin peel powder (5%, 10%, and 20%) and seeds powder (5%, 10%, 15%, and 20%) were mixed with commercially available wheat flour to produce cookies and noodles. Furthermore, the nutritional, antioxidant, functional and sensory properties of the developed cookies and noodles were evaluated.

Result: ~~There were significant differences ($p<0.05$)~~Pumkin peels and seeds powder and their respective products enrich significantly in protein, fiber, beta-carotene, antioxidants such as total phenolic content (TPC), 2,2-diphenyl-1-picrylhydrazyl (DPPH scavenging activity), Ferric reducing ability (FRAP Ferric reducing power assay), ash content and hunter color values~~ between powders from pumpkin seeds and peels powders~~. However, pumpkin seed powder had higher protein content (33.25%) than other powders but pumpkin peel powder (16.83%±1.74) had better fiber content than seeds powder. The highest amount of fiber content was found in 10% pumpkin peel cookies. . The analysis of the composition of pumpkin peels and seeds powder has been done in order to further promote their functionality in bakery products based, especially, on wheat flour.

Conclusion: The best quality and overall acceptable product were obtained when samples were 10% pumpkin peel powder, 15% pumpkin seed powder, and, 10% pumpkin peel powder noodles.

Keywords: Pumpkin,Seeds,Peels,Cookies,Noodles,NutritionalProperties.

1. INTRODUCTION

Pumpkin (*Cucurbita moschata*) is a fruit vegetable, not only grown in the Western Hemisphere but also in Tropical Asia in countries such as Bangladesh, India, Indonesia, Malaysia and Philippines [1].The total world agricultural production has increased annually ~~overthe~~over the ~~past10~~years from ~~50~~111.986 million tons in ~~1996~~2018 to ~~203~~136.27 million tons in ~~2005~~2020 while in Bangladesh pumpkin cultivation has an annual production of 340 thousand metric tons [1,2].

Pumpkins have a large range of uses as a potentially valuable food source for both humans and animals. The edible flesh (pulp) of the pumpkin fruit may be eaten as a vegetable or incorporated into food products such as curry, pies, bread, soup, or jams. The food industry utilizes pumpkin powders and flakes as thickeners, flavoring and coloring agents in soups and sauces [1, 3]. However, food processing techniques create large quantities of waste in the form of peels (skin) and seeds. These waste streams can be utilized as an added value ingredient with the potential of being processed into food products in a similar way to other waste stream products and fiber-rich components [4-6].

Pumpkin peels and seeds are rich in vitamins, proteins, minerals and antioxidants (β-carotene and tocopherols) [7,8]. In addition to nutrient composition, it is composed of various biological active components such as polysaccharides, protein, peptide, sterols and para-amino benzoic acids [7, 9]. These biological active components have shown to be wide range of medicinal properties such as anti-diabetic activity, antioxidant activity, anti-carcinogenic effect and anti-microbial effect [7, 10].

With these beneficial effects of pumpkin peels and seeds, it can be incorporated into different value-added products such as cookies and snacks (noodles). In recent times, fruits and vegetables by-products ~~has~~have attracted more attention due to their health benefits including pumpkin pie, biscuits, bread, desserts, puddings, beverages, and soups. Pumpkins are celebrated in festivals and in flower and vegetable shows in many countries. Therefore, it has been focused on under-utilized indigenous crops waste, for example the pumpkin peels and seeds are useful in food

industries for the formulation of value-added products. "Several previous studies have demonstrated the strong antioxidant activity of pumpkin peels and seeds (Apostol et al., 2017; Stevenson et al., 2007) and their potential role in preventing hypertension and carcinogenic diseases (Zuhair et al., 2000; Jian et al., 2005) [37, 39-41]. Although there have been studies on formulations of cookies and noodles incorporating pumpkin peels and seeds, mixtures of wheat flour with various added ingredients are known to be rich in numerous active principles such as antioxidants, macro elements, microelements, and fiber, making them highly valuable raw materials for the confectionery industry [37].

In recent times, there has been increasing attention toward finding uses for by-products and waste generated from food processing. Utilizing these by-products would not only help maximize available resources but also introduce new food products to the market, while simultaneously addressing waste disposal issues."~~Although there have been studies with cookies and noodles formulations with pumpkin peels and seeds.~~

Therefore, the objective of this study was to utilize pumpkin by-products and develop pumpkin peels and seeds powders mixed cookies and noodles and evaluation of its physical, chemical and sensory properties.

2. MATERIALS AND METHODS

2.1 Raw Materials, chemicals and other ingredients

Fully ~~Ripe~~ ripe pumpkins (Cucurbita moschata) were sourced from a local market in Dhaka, Bangladesh chosen for uniformity, color, and lack of defects during July-august, 2023 harvest season. Pumpkin seeds, wheat flour (Local Brand: ACI Pure flour), oil, sugar, skimmed milk powder, butter, eggs, baking powder, salt, and margarine were obtained locally. Citric acid and packaging materials came from a local company. All items, except butter and eggs stored in a refrigerator, were kept at room temperature. High-density polyethylene bags stored peel powder, seed powder, cookies, and noodle samples. All analytical grade chemicals were supplied by the food analytical laboratory and food processing laboratory, State University of Bangladesh, Dhaka.

2.2 Preparation of pumpkin peels and seeds powder

Ripe pumpkin was washed, sliced, and its peels collected. The peels were treated with 0.5% citric acid, dried at 65°C for 8 hours. Purchased seeds were oven-dried at 80°C for 22 hours. Both dried peels and seeds (With husk) were blended by blender machine (Jaipan CM/L -7360065,), sieved by A.S.T.M. (E-11), Endecotts limited, London, England through a 50-mesh size, and packed in high-density polyethylene bags for analysis.

2.3 Preparation of cookies and noodles from pumpkin seeds and peels

2.3.1 Powders Formulation of Cookies

There were two types of cookies were formulated. Cookies were prepared from pumpkin seeds and peels powders. Pumpkin seeds powders and peels powders were substituted at 5%, 10%, 15%, 20% and 5%, 10%, 20%, respectively with marketed wheat flour which were represented in the figure 01 and 02. Cookies prepared from market wheat flour serve as control. The list of the samples are

S_A = P_A = Control (100% market wheat flour, Cookie)
S_B = 95% market wheat flour /5%pumpkin seed cookie
S_C = 90% market wheat flour /10%pumpkin seed cookie
S_D = 85% market wheat flour /15%pumpkin seed cookie
S_E = 80%market wheat flour /20%pumpkin seed cookie

P_B = 95%wheat/5%pumpkin peel cookie
P_C = 90%wheat/10%pumpkin peel cookie
P_D = 80%wheat/20%pumpkin peel cookie

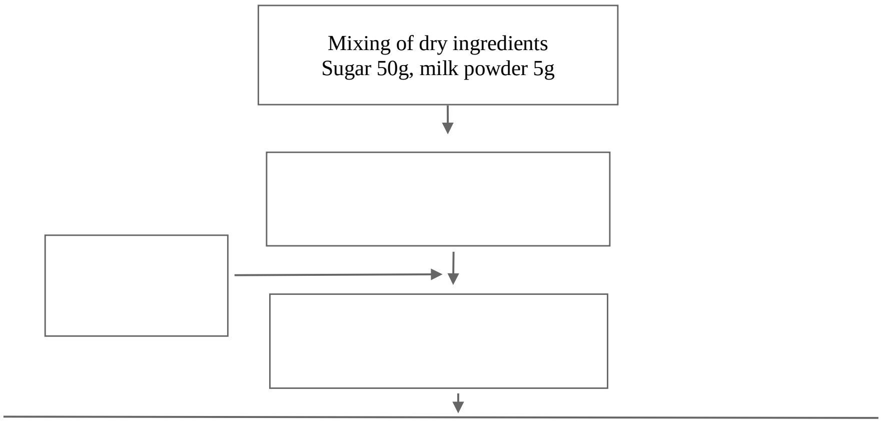

Formatted: Subscript
Formatted: Subscript
Formatted: Subscript
Formatted: Subscript

Figure 01: Flow diagram of preparation of pumpkin peel cookies samples

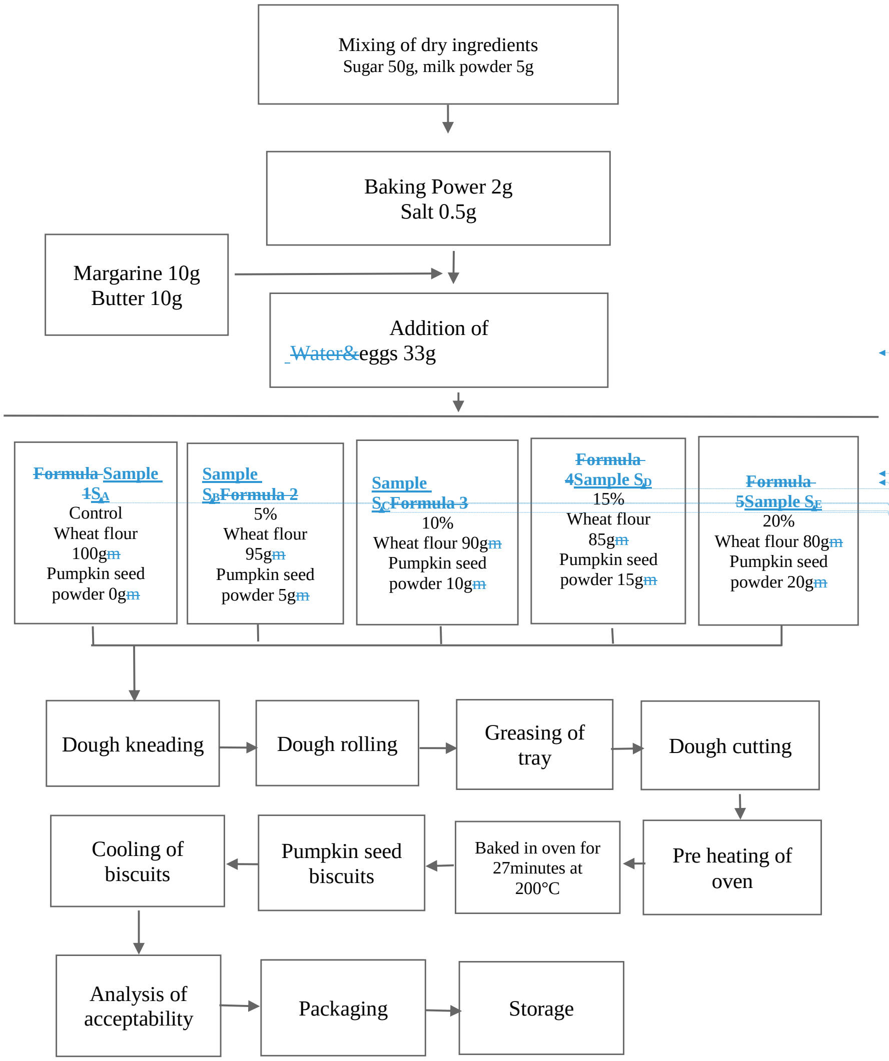

Flow diagram 02: Preparation of pumpkin seed cookies samples

2.3.2 Formulation of Noodles

Noodles were also made from pumpkin seeds powder and pumpkin peels powders. Three types of noodles were formulated such as wheat four fortified with 10% seed_powder,_10% peel powder_and_10% seed_powder_mixed_with 10% peel powder which were represented in the figure 03.Noodles prepared from market wheat flour serve as control._The list of the noodles samples are

N_A = Control (100% wheat flour noodles)
N_B = 90%wheat/10% pumpkin seeds noodles
N_C = 90%wheat/[illegible]ls noodles
N_D = 80%wheat/[illegible]ls and 10% pumpkin peels noodles

Dough kneading

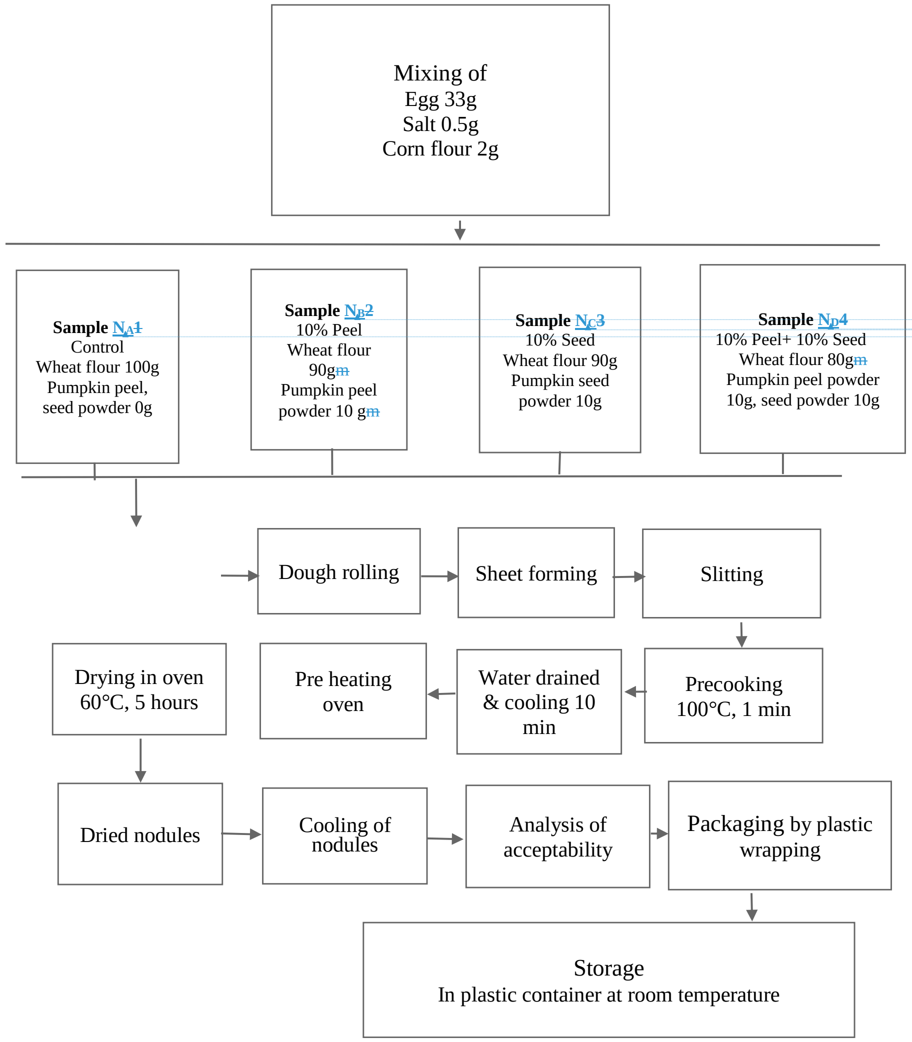

Formatted: Subscript
Formatted: Subscript
Formatted: Subscript
Formatted: Subscript

Figure 03: Flow diagram of Preparation of noodles samples

2.4 Proximate composition

Moisture (oven drying method), protein (Kjeldahl method, where the N conversion factor is 6.25), fat (Soxhlet method), ~~and~~ ash (Muffle furnace method) and crude fiber content of ~~sweet potato~~pumpkin seeds powder, peels powders~~ and~~, cookies and noodles ~~baked cakes~~ were determined by official methods of Association of Official Analytical chemists (AOAC, 1984; 1998) [11] and the gluten content was determined according to the method reported by Taneya et al. (2014) [30]. ~~Carbohydratewasexpressedasthedifferencefrom moisture,protein,fat,andash.Thecaloricvaluewascalculatedtoconversionfactorsof4kcalg-1 for proteins, 9 kcal g-1 for fats and 4 kcal g-1 for carbohydrates [12]~~. ~~All the analyses were done in triplicates.~~

~~2.5 Determinationofcrudefiber~~

Acid and alkali hydrolysis followed by ignition of the residue as described by the method AOAC (1984) was used to estimate the fiber content in ~~sweet potato~~pumpkin seeds & peels powders and ~~cakes~~ cookies and noodles samples [11]. All the analyses were done in triplicates. Then the fiber content was determined by following equation

$$\%\ of Fibre = \frac{Initial\ weight - Final\ Weight}{Sample\ weight} \times 100$$

2.6 5 Determination of total polyphenol content

Determination of the total polyphenol content was done by Sorifa et al., (2010) method with some modifications [13]. 1 gram of samples was extracted using 20 ml of 25% ethanol for 15 minutes and subsequently filtered through Whatman no. 2 filter paper. Following this, a mixture of 1 ml of the sample, 1 ml of Folin reagent, and 5 ml of Na_2CO_3 was transferred to a volumetric flask and left at room temperature for 1 hour. Total phenols were calculated based on standard curves of gallic acid and expressed as mg/1 g utilizing spectrophotometer (T80 U/VIS, United Kingdom).

2.7 6 Determination of DPPH:

The scavenging effects of samples for 1,1-diphenyl-2-picrylhydrazyl (DPPH) radical were monitored according to the modified method of the previous report by Sorifa et al., 2010 [13]. The scavenging effects of the samples on 1,1-diphenyl-2-picrylhydrazyl (DPPH) radical were evaluated following a modified method based on the previous report by Sorifa et al. (2010). Each sample (1 milliliter) was combined with 5 milliliters of freshly prepared 0.1 mM DPPH methanolic solution. The resulting mixture was shaken and incubated in darkness for 50 minutes at room temperature. Absorbance was subsequently measured at 517 nm using a spectrophotometer (T80 U/VIS, United Kingdom The DPPH radical scavenging activity was then calculated as follows:

$$Scavenging\ effect\ (\%) = \left[Abs_{control} - \left(Abs_{sample} - Abs_{sample\ backgroung}\right)\right] \times 100/Abs_{control}$$

2.8 7 Determination of FRAP

ThechelatingcapacityofPSEwasdeterminedwithamethoddescribedbySorifaetal.,2010 [13].The chelating capacity of PSE was assessed using a method outlined by Sorifa et al. (2010). Various concentrations of samples (0.1 mL) were combined with 0.1 mL of 2 mM $FeCl_2 \cdot 4H_2O$ and 0.2 mL of 5 mM ferrozine. Subsequently, 3.7 mL of MeOH was added to the mixture in a test tube and allowed to react for 10 minutes. The absorbance at 562 nm was then measured, and chelating activity was determined using the following equation:

$$Chelating\ activity\ (\%) = \left[Abs_{control} - \left(Abs_{sample} - Abs_{sample\ backgroung}\right)\right] \times 100/Abs_{control}$$

2.9 8 Cooking properties

The cooking properties of noodles were evaluated by a method similar to that described by Mestres et al., 1988 [14]. Noodles weighing 1g were added to 150mL of boiling water and cooked for 1 minute to achieve optimal cooking. The sample was subsequently drained for 5 minutes and swiftly weighed (W_1, g). The cooked product was then dried in an oven at 130°C until a constant weight was achieved (W_2, g). The cooking water was centrifuged at 7500 rpm for 10 minutes, and the dry matter contents of the sediment and supernatant (W_3, g and W_4, g, respectively) were determined. These evaluations of cooking properties were all done in duplicate. Cooking yield and losses were calculated with the following equations (DM = dry matter ratio of crude samples):

$$Cooking\ yield\ (\%) = \frac{(W_2) \times 100}{W_1}$$

Formatted: Font: (Default) Times New Roman, 12 pt

Formatted: Font: (Default) Times New Roman, 12 pt, Subscript

Formatted: Font: (Default) Times New Roman, 12 pt

Formatted: Font: (Default) Times New Roman, 12 pt, Subscript

Formatted: Font: (Default) Times New Roman, 12 pt

$$Cooking\ loss\ (\%) = \frac{(DM - W_2) \times 100}{DM}$$

These evaluations of cooking properties were all done in duplicate.

2.10 9 Sensory evaluation

A sensory analysis of cookies was conducted using a 9-point Hedonic scale by a panel of 10 members from the Department of Food Engineering and Technology at the State University of Bangladesh, Dhaka. The assessment covered attributes such as color, flavor, taste, texture, and overall acceptability. Similarly, sensory evaluations of both cookies and noodle samples were performed by a taste-testing panel, employing a hedonic rating test with ten panelists. They were tasked with evaluating various sensory parameters and assigning scores on a nine-point hedonic scale, where 9 represented 'Like extremely,' 8 for 'Like very much,' and so forth. Preference differences were subsequently evaluated through statistical analysis of the data for variance.

Cookies underwent sensory analysis using a 9-point Hedonic scale by 10 panelists from the Department of Food Engineering and Technology, State University of Bangladesh. Attributes such as color, flavor, taste, texture, and overall acceptability were assessed. Statistical analysis, including variance and Duncan's Multiple Range Test (DMRT), was performed using the Statistical Analysis System (SAS, 1985) procedures.

2.11 10 Statistical analysis

The results were expressed as the mean, standard error deviationof the mean (SEM), and coefficient of variation of each species for each parameter was determined. All evaluations of samples were all done in triplicate. Data were statistically analyzed (R statistical software 3.4.1) by one-way analysis of variance (ANOVA). **Statistical analysis for the sensory analysis, including variance and Duncan's Multiple Range Test (DMRT), was performed using the Statistical Analysis System (SAS, 1985) procedures.** Mean comparisons were performed using Duncan's multiple range tests for significant effect at $P < 0.05$.

Formatted: Normal, Left, Indent: Left: 0", Right: 0", Line spacing: single

3. RESULT AND DISCUSSION

3.1 Proximate analysis

3.1.1 Proximate analysis of wheat flour, pumpkin seeds and peels powder

The three kinds of powders were analyzed for proximate analyses which are wheat flour which collected from local market, pumpkin seed powder and pumpkin peel powder. The results showed in the table 01 where results showed significantly higher amounts of moisture, ash, protein, fat, fiber and beta-carotene contents in pumpkin seeds powder and peels powder as compared to marketed wheat flour (control). Similar results were observed for pumpkin seeds powder and peels powder by Ashik et al., 2022; who reported that moisture, ash, protein, fat and fiber content of pumpkin seeds powder was 2.75%, 5.20%, 34.19%,44.01% and 4.12 %, respectively where as pumpkin peels powder was 10.31%,6.04%, 12.30 %,5.77 % and 13.91%, respectively [15].Sometimes the difference in protein and fat value in pumpkin seeds and peels were observed. This might be due to the variation in environment during harvesting such as application of fertilizer (nitrogen content), growing conditions and time and also location of growing areas, oxidation of the fat because most of the fat content in seeds and peels is unsaturated and undergo oxidation easily by atmospheric oxygen. Atmospheric oxidation normally product of alkyl peroxyls of hydrocarbons. Normaly when the concentration is low, they can react with themselves which is called self-reaction of alkyl peroxyl radicals. If the concentration is high, in the unsaturated condition the oxygen molecules are added in the subsequent stage, and the self reactions can be lead to aldehydes and alcohols [29]. The gluten content of the wheat flour which collected form local renowned company was 14.47% while pumpkin seeds and peels powder was no gluten, which also we found that the gluten value of Bangladesh around 11% to 15% [31].

Table01: Proximate compositions of powder samples

Powder Samples	Moisture Content (%)	Ash Content (%)	Protein Content (%)	Fat Content (%)	Fiber Content (%)	Beta- carotene (μg/g)	Gluten ([illegible]
Wheat flour	10.60±0.87^{a}	0.57±0.02^{b}	9.62 ±0.01^{b}	1.08±0.03^{ba}	0.51±0.09^{b}	14.56±0.34^{b}	14.45±0.76^{a}
Pumpkin seed powder	3.21±0.16^{ba}	5.18±0.03^{a}	33.25±0.01^{a}	43.96±0.33^{a}	3.9.1±0.48^{b}	39.46±0.36^{b}	0
Pumpkin peel powder	10.21±0.13^{a}	4.40±0.03^{a}	15.09±0.30^{ab}	2.94±0.01^{ba}	16.83±1.74^{a}	123.55±0.33^{a}	0

All values are express as mean ± SD.
Mean followed by different superscript letters in each column are significantly different (p<0.05)

3.1.2 Proximate analysis of pumpkin seed cookies

The proximate compositions of cookies from pumpkin seeds was analyzed for moisture, ash, protein, fat, fiber and beta-carotene which presented in Table 02. Results demonstrated that all pumpkin seed cookies had higher amount of protein, fat, fiber and beta-carotene content as compared to control (100% wheat flour cookie).

Protein content of different cookies varied from 8.09 to 12.03/100 g that was similar to found by Alshehry (2020), reported protein value of in pumpkin seed cookies 12.62 to 13.75% on the variation of the percentage of the pumpkin seeds powder using to develop, Syam et al. (2019) who reported protein value in pumpkin seed cookie 11.20/100 g and also by Dhiman et al. (2018) who reported protein value in cookies as 8.47 to 12.56/100g [16, 17, 32]. Sample ~~E~~S_E had highest protein value (12.03/100g) compared with other recipes. On the contrary, sample S_A which is backed by market wheat flour had the lowest value of protein (8.09/100 g) than other samples. The variations in protein value increase because pumpkin seed contain more protein than market wheat flour.

Fat values were appreciably different among cookies and highest was found in sample S_D (13.95/100g), where lowest content (11.62/100g) were studied in sample S_A. This observation was comparable to that found by Alshehry (2020) ~~Syam et al. (2019)~~ who reported fat value in pumpkin seed cookies 7.13~~33.05~~/100g [~~17~~32].The variation in fat value in different cookies may be due to oxidation of the fat because most of the fat content in cookies is unsaturated and undergo oxidation easily by atmospheric oxygen. The ash content of different cookies varied between 0.91/100 to 1.36/100g. This result was consistent to be found by Alshehry (2020) and Syam et al. (2019) who reported ash content in cookies as 1.62/100g and 1.65/100g respectively [17]. Lowest ash value was found in sample S_A which is backed from market wheat flour (0.91/100g) variety while highest value (1.36/100g) was studied in sample S_E which backed from 85% market wheat flour /15% pumpkin seed cookie.

Fiber values were appreciably different among cookies and highest was found in sample S_D (1.61/100g), where lowest content (0.32/100g) were studied in sample S_A. This observation was comparable to that found by Apostol et al. (2020), Alshehry (2020) and Syam et al. (2019) who reported fat value in pumpkin seed cookies 5.74/100g, 6.24/100g and 1.64/100g respectively [17, 32, 37]. Low molecular weight components such as minerals, vitamins and sugars might be lost during washing treatment, leading to a relative increase in the dietary fibre content (Wenberg et al., 2006). The Beta-carotene content of different cookies varied between 4.07 ug/g to 6.36 ug/g. This result was consistent to be found by Dhiman et al. (2018) who reported Beta-carotene

Formatted: Font: 12 pt, Subscript
Formatted: Font: 12 pt, Subscript
Formatted: Font: 12 pt, Subscript
Formatted: Font: 12 pt, Subscript
Formatted: Font: 12 pt, Subscript
Formatted: Font: 12 pt, Subscript
Formatted: Font: 12 pt, Subscript
Formatted: Font: 12 pt, Subscript

content in cookies as 3.22 to 3.78 mg/100g [16]. Lowest Beta-carotene content was found in sample S_A which is backed from market wheat flour (4.07ug/g) variety while highest value (6.36ug/g) was studied in sample S_E which backed from 80% market wheat flour 20% pumpkin seed cookie.

Table 02: Proximate compositions of pumpkin seeds cookies

Cookie Samples	Moisture content (%)	Ash content (%)	Protein content (%)	Fat content (%)	Fiber content (%)	Beta-carotene (ug/g)
S_A	6.68 ± 0.19^{bc}	0.91 ± 0.01^{c}	8.09 ± 1.54^{c}	11.62 ± 0.56^{d}	0.32 ± 0.15^{c}	4.07 ± 0.36^{c}
S_B	5.91 ± 0.31^{c}	1.02 ± 0.03^{c}	9.84 ± 0.31^{b}	12.03 ± 0.02^{cd}	0.62 ± 0.19^{bc}	5.18 ± 0.34^{b}
S_C	7.79 ± 0.56^{ab}	1.24 ± 0.01^{ab}	9.84 ± 0.31^{b}	12.89 ± 0.01^{bc}	0.92 ± 0.06^{b}	5.27 ± 0.39^{ab}
S_D	7.96 ± 0.06^{a}	1.15 ± 0.02^{b}	11.15 ± 0.31^{a}	13.95 ± 0.70^{a}	1.61 ± 0.31^{a}	5.77 ± 0.74^{ab}
S_E	7.74 ± 0.54^{ab}	1.36 ± 0.03^{a}	12.03 ± 0.31^{a}	13.78 ± 0.86^{ab}	1.52 ± 0.30^{a}	6.36 ± 1.08^{a}

Values are means of triplicate analysis ± Standard deviation. S_A=Control (100% market wheat flour, Cookie), S_B=95% market wheat flour /5%pumpkin seed cookie, S_C=90% market wheat flour /10%pumpkin seed cookie, S_D=85% market wheat flour /15%pumpkin seed cookie, S_E=80%market wheat flour /20%pumpkin seed cookie)

All values are express as mean ± SD

Mean followed by different superscript letters in each column are significantly different ($p<0.05$)

3.1.3 Proximate compositions of pumpkin peel cookies

The composition of cookies from pumpkin peels was analyzed for moisture, ash, protein, fat, fiber and beta-carotene. The results presented in table 03 where sample P_D contained the highest amount of moisture and sample P_A had the lowest level of moisture. These values were higher to those reported by Mishra and Sharma (2019) who found that moisture content in cookies is 6.59/100g and also by Saleh and Ali (2020) found that moisture content in pumpkin peel cookie was 5.62/100g to 7.09/100g [18, 19].

Protein content of different pumpkin peel cookies varied from 8.09 to 9.04/100g that was similar to found by Saleh and Ali (2020) who reported protein value in pumpkin peel cookie 7.73/100g to 9.42/100g and higher found by Mishra and Sharma (2019) who reported protein value in

cookies as 0.08/100g [18]. Sample P_D had highest protein value (9.40/100g) compared with other recipes. On the contrary, sample P_A which is backed by market wheat flour had the lowest value of protein (8.09/100 g) than other samples. The ~~variation in protein value increase~~variations in protein value increase because pumpkin peel contains more protein than market wheat flour.

Fat values were appreciably different among cookies and highest was found in sample P_C (11/100g), where lowest content (10.84/100g) were studied in sample P_A. This observation was comparable to that found by Mishra and Sharma (2019) who reported fat value in pumpkin peel cookies 21.75/100g and also found by Saleh and Ali (2020) who reported fat value in pumpkin peel cookie 14.82/100g to 15.14/100g [18, 19]. The variation in fat value in different cookies may be due to oxidation of the fat because most of the fat content in cookies is unsaturated and undergo oxidation easily by atmospheric oxygen. The ash content of different cookies varied between 0.91/100 to 1.34/100g. This result was consistent to be found by Saleh and Ali (2020) who reported ash content in cookies as 1.30/100g to 2.62/100g [19].Lowest ash value was found in sample P_A which is backed from market wheat flour (0.91/100 g) variety while highest value (1.34/100 g) was studied in sample P_D which backed from 85% market wheat flour /15%pumpkin peel cookie.

Fiber values were appreciably different among cookies and highest was found in sample P_C (5.08/100g), where lowest content (0.32/100g) were studied in sample P_A. This observation was comparable to that found by Saleh and Ali (2020) who reported fiber value in pumpkin peel cookies1.18/100g to 3.17/100 g and higher then found by Mishra and Sharma (2019) who reported fiber value in pumpkin peel cookies 0.16/100g [18, 19].

The Beta-carotene content of different cookies varied between 4.07ug/g to 29.38ug/g. This result was ~~highertо~~higher to be found by Saleh and ~~Ali(~~Ali (2020) who reported Beta-carotene content in cookies as

3.08 to 11.54 mg/100g [19]. Lowest Beta-carotene content was found in sample A which is backed from market wheat flour (4.07ug/g) variety while highest value (39.38ug/g) was studied in sample P_D which backed from 80% market wheat flour /20%pumpkin peel cookie. The variation in beta- carotene value increase because pumpkin peel contain more beta-carotene then market wheat flour.

Table 03: Proximate compositions of pumpkin peel cookies

Cookie Samples	Moisture Content (%)	Ash Content (%)	Protein Content (%)	Fat Content (%)	Fiber Content (%)	Beta-carotene (ug/g)
P_A	6.68 ±0.19^c	0.91±0.01^c	8.09 ±1.54^a	11.62±0.56^a	0.32±0.15^c	4.07±0.36^c

P_B	7.17±0.17^{bc}	1.04±0.01^{b}	8.75±0.61^{a}	10.84±0.16^{b}	2.07±0.19^{bc}	12.40±0.36^{b}
P_C	7.63±0.52^{b}	1.10±0.02^{b}	8.96 ±0.31^{a}	11.00±0.21^{ab}	5.08±0.06^{a}	25.52±0.19^{a}
P_D	9.02± 0.43^{a}	1.34±0.01^{a}	9.40±0.30^{a}	10.95±0.13^{ab}	3.08±0.31^{ab}	29.38±0.37^{a}

Values are means of triplicate analysis ± Standard deviation. P_A=Control (100% wheat, Cookie), P_B=95%wheat/5%pumpkin peel cookie, P_C=90%wheat/10%pumpkin peel cookie, P_D=80%wheat/20%pumpkin peel cookie)
All values are express as mean ± SD.
Mean followed by different superscript letters in each column are significantly different ($p<0.05$)

3.1.4 Proximate compositions of noodles samples:

The composition of noodles from pumpkin seeds and peels powder was analyzed for moisture content, protein, ash and fat content. The results obtained were presented in Table 04. Sample had lower moisture content as compared to all other peels and seeds fortified noodles. Although these values had no significant ~~differences.The~~differences. The protein content of noodles sample ranged from 4.81 to 5.47 %. There were no significant differences in protein content among all noodles sample. All noodles' samples had fat content ranged from 0.59 to 4.96%. Sample N_D (80% wheat/ 10% pumpkin seeds and 10% pumpkin peels noodles) had highest fat content which is slightly higher than control. The ash content of noodles sample ranged from 1.60 to 2.2 7%. Sample N_D (80%wheat/10%pumpkin seeds and 10% pumpkin peels noodles) sample had highest ash content. There was significantly different in ash content was observed for sample N_D. This might be due to the higher amount of mineral content present in the seeds and peels samples. The fiber content of noodles sample ranged from 1.49 to 8.88%. Control (wheat flour noodles) sample had lower fiber content as compared to all other peels and seeds fortified noodles.

Table04: Proximate compositions of noodles samples.

Noodles Samples	Moisture Content(%)	AshContent (%)	Protein Content (%)	Fat Content (%)	Fiber Content (%)	Beta-carotene (ug/g)
N_A	8.94±0.62^{a}	1.60±0.026^{b}	5.47 ±0.31^{a}	4.30±0.47^{a}	1.49 ±0.23^{a}	0.324 ±0.003^{a}
N_B	9.67±0.97^{a}	1.85±0.20^{b}	5.03±0.30^{a}	0.59 ±0.01^{a}	6.36 ±1.58^{a}	0.315 ±0.004^{a}
N_C	9.44±0.85^{a}	1.72±0.034^{b}	4.81±0.02^{a}	4.96 ±0.44^{a}	8.88±1.13^{a}	0.222±0.004^{b}
N_D	9.23±0.42^{a}	2.27±0.140^{a}	5.25 ±0.03^{a}	0.59± 0.07^{a}	8.82 ± 0.19^{a}	0.318 ± 0.002^{a}

Values are means of triplicate analysis ± Standard deviation. N_A=Control (100% wheat flour noodles), N_B=90%wheat/10% pumpkin seeds noodles, N_C=90%wheat/10% pumpkin peels noodles, N_D=80%wheat/10%pumpkin seeds and 10% pumpkin peels noodles
All values are express as mean ± SD.
Mean followed by different superscript letters in each column are significantly different ($p<0.05$)

3.~~2~~ 1.5 β-Carotene determination for noodles

Almeida et al. (2011) reported that, foods which are rich in antioxidants play an essential role in

Formatted: Subscript
Formatted: Subscript
Formatted: Subscript
Formatted: Subscript
Formatted: Subscript
Formatted: Subscript
Formatted: Subscript
Formatted: Subscript
Formatted: Subscript
Formatted: Subscript
Formatted: Font: 11 pt, Subscript
Formatted: Font: 11 pt, Subscript
Formatted: Font: 11 pt, Subscript
Formatted: Font: 11 pt, Subscript
Formatted: Subscript
Formatted: Subscript
Formatted: Subscript
Formatted: Subscript

the prevention of carcinogenic diseases. The antioxidant capacities of leaves samples such as pumpkin peels and seeds may vary depending on their contents of vitamin C, vitamin E, carotenoids, and particularly β-carotene [20], and lycopene [21] as well as other pigments such as flavonoids and other polyphenols [22]. The results for β-carotene content of the noodles are listed on Table 04. Carotenoids are tetraterpenoids found throughout not only the flowering plant kingdom but also in vegetables and fruits as a pigment mostly responsible for various color such as red, orange or yellow color in peels, seeds, flash leaves and fruits which are important as vitamin A precursors. As they are found widely in all kinds' plants and plant parts, it is not surprising that a large number of carotenoidcarotenoids shave been reported in the pumpkin seeds and peels powder [23]. Noodles from pumpkin seeds powder showed the highest ($P < 0.05$) content of β-carotene except control. Noodles manufactured from peels powder and mixture of peels and seeds have also shown to be an excellent source of β-carotene. Similar levels of β-carotene have been previously reported for pumpkin flesh powder [24]. However, these pumpkin peels and seeds powder should not be considered as a rich source of carotenoids, where values as high as 161mg/100 gd.b. for wine palm (*Mauritiavinifera*)oneofone of the most important vitamin A precursors has been represented [25, 26].

3.23 Cooking properties (Cooking yield)

Generally speaking, high cooking yield gains and low cooking loss would be desirable characteristics for high quality of any kinds of noodles. Flour noodles with added different types of pumpkin powder such as peels powder, seeds powder and mixture of peels and seeds powders had lower cooking yield gains than the flour noodle samples with no added pumpkin (Table 05). It was found that control sample (N_A) contained the highest amount of cooking yield and 10% seeds powder noodles sample (N_B) had the lowest level of cooking yield while 10% pumpkin seeds powder has lowest cooking loss and the mix powder has highest cooking loss. Including controls all noodles' samples cooking loss is considerable higher then Apostol et al. (2020) & Mestres et al. (1988) [14]. But all noodles' samples cooking yield is much higher [24].

The parameters of cooked noodles varied considerably for gumminess and hardness. However, springiness, cohesiveness, and chewiness were not different among the samples, indicating the pumpkin powder did not affect such parameters [24, 27].

Table 05: Cooking properties of noodles

Sample	Noodles cooking Yield (%)	Noodles cooking Loss (%)
N_A Control	275.254±0.10^a	3.723±0.010^b
N_B 10%seedspowder	256.368±0.10^b	3.421 ±0.010^b
N_C 10%Peels powder	250.885±0.10^b	4.217±0.010^{ab}
N_D 10%seeds+10%Peelspowder	260.089±0.01^b	5.007±0.002^a

Formatted: Subscript

Formatted: Subscript

Values are means of triplicate analysis ± Standard deviation. N_A=Control (100% wheat flour noodles), N_B=90%wheat/10% pumpkin seeds noodles, N_C=90%wheat/10% pumpkin peels noodles, N_D=80%wheat/10%pumpkin seeds and 10% pumpkin peels noodles
All values are express as mean±SD.
Mean followed by different superscript letters in each column are significantly different ($p<0.05$)

3.34 Hunter Color Values

Hunter color values were represented by L*, a*, b*, and ΔE where L* is a measure of lightness, a* redness, b* yellowness and ΔE color differences. The Hunter color parameters L*, a*, b*, and ΔE have been widely used to evaluate color changes during dehydration of fruits and vegetable products. There are three types of powdered samples such as wheat flour which collected from local market served as control, Pumpkin seed powder and Pumpkin peel powder. The results of Hunter Color Values (L*,a*,b*,ΔE) of pumpkin seeds and peels powder are represented in table 06, pumpkin seed cookies are shown in Table 07 & pumpkin peels cookies are shown in Table 08.

Results indicates lightly decrease in L*, b* and ΔE value while increase in a* value of all cookies samples prepared from both seeds and peels powders with the level of increasing sample concentrations. The increase of hunter a* values (redness) with increase of sample concentration for all cookie samples might be due to the influence of beta-carotene concentration. The flour colors and cookies color can best be described by the change in total color difference, ΔE values, where locally collected wheat flour has a significance difference with both pumpkin seeds and peels powder. Pumpkin seeds cookies S_A, S_E and pumpkin peels cookies P_A, P_B and P_c shows almost similar results. The lower ΔE values may be due to the loss of phenolics and oxidation during drying, boiling treatment and oven temperature [28]. ~~Therewereno significant differences in ΔE values among all cookie's samples.~~

Table 06: Hunter Color Values (L*, a*, b*,ΔE) of pumpkin seeds and peels powder samples

Powder Samples	L*	a*	b*	ΔE
Wheat Flour	80.53±2.87^{a}	1.82±0.19^{a}	10.57±0.55^{b}	71.98±13.59^{a}
Pumpkin seed powder	52.79±1.24^{b}	3.57±0.74^{a}	19.46±1.35^{a}	12.62±2.58^{a}
Pumpkin peel powder	65.09±4.74^{ab}	1.34±0.26^{a}	18.72±4.27^{a}	17.51±2.38^{a}

All values are express as mean ± SD.
Mean followed by different superscript letters in each column are significantly different ($p<0.05$).

Table 07: Hunter Color Values (L*, a*,b*,ΔE) of pumpkin seeds cookies

Cookie Samples	L*	a*	b*	ΔE
S_A ~~A~~	50.94±1.85^{ab}	16.20±0.80^{a}	28.46±0.45^{a}	60.56±1.98^{ab}

S_BB	49.14±1.90^{ab}	13.51±0.96^{b}	26.88±0.41^{ab}	57.621±2.04^{ab}
S_CC	44.04±2.93^{b}	14.40±1.45^{ab}	23.40±2.43^{b}	51.91±3.98^{b}
S_DD	43.02±0.41^{b}	14.46±0.48^{ab}	24.50±0.82^{ab}	51.58±.87^{b}
S_EE	54.07±1.67^{a}	13.95±1.18^{ab}	27.60±0.13^{ab}	62.29±1.77^{a}

Values are means of triplicate analysis ± Standard deviation. S_A=Control (100% market wheat flour, Cookie), S_B=95% market wheat flour /5%pumpkin seed cookie, S_C=90% market wheat flour /10%pumpkin seed cookie, S_D=85% market wheat flour /15%pumpkin seed cookie, S_E=80%market wheat flour /20%pumpkin seed cookie)
All values are express as mean ± SD
Mean followed by different superscript letters in each column are significantly different ($p<0.05$)
Valuesaremeansoftriplicateanalysis±Standarddeviation.A=Control(100%wheat,Cookie),B=95%wheat/5%pumpkinseedcookie,C=90%wheat/10%pumpkin seed cookie, D=85%wheat/15%pumpkin seed cookie, E=80%wheat/20%pumpkin seed cookie)
Allvaluesare expressasmean±SD.
Meanfollowedbydifferentsuperscriptlettersineachcolumnaresignificantlydifferent($p<0.05$)

Table 08: Hunter Color Values (L*, a*,b*,ΔE) of pumpkin peels cookies

Cookie Samples	L*	a*	b*	ΔE
P_AA	50.94±1.85^{a}	16.20±0.80^{a}	28.46±0.45^{a}	60.56±1.98^{a}
P_BB	55.84±1.60^{a}	10.67±0.19^{b}	28.97±0.24^{a}	63.81±1.54^{a}
P_CC	51.19±1.13^{a}	9.70±0.72^{b}	29.48±0.86^{a}	59.86±1.51^{a}
P_DD	41.50±2.34^{b}	13.25±0.61^{ab}	24.08±2.27^{b}	49.78±3.21^{b}

Values are means of triplicate analysis ± Standard deviation. P_A=Control (100% wheat, Cookie), P_B=95%wheat/5%pumpkin peel cookie, P_C=90%wheat/10%pumpkin peel cookie, P_D=80%wheat/20%pumpkin peel cookie)
All values are express as mean ± SD.
Mean followed by different superscript letters in each column are significantly different ($p<0.05$)
Valuesaremeansoftriplicateanalysis±Standarddeviation.A=Control(100%wheat, Cookie),B=95%wheat/5%pumpkinpeel cookie,C=90%wheat/10%pumpkin peel cookie, D=80%wheat/20%pumpkin peel cookie)
Allvaluesareexpressasmean±SD.
Meanfollowedbydifferentsuperscriptlettersineachcolumnaresignificantlydifferent($p<0.05$)

3.5 4 Antioxidant Properties

3.54.1 Total Phenolic Compound and Antioxidant Properties (DPPH scavenging activity and FRAP Ferric reducing power assay) of powder samples

The total phenolic compound, DPPH and FRAP antioxidant properties of market wheat flour, pumpkin seed powder and pumpkin peel powder are shown in Table 09. The highest total phenolic compound (268.38mg/100g) was found in pumpkin seed powder sample. It is commonly known as seeds are an excellent source of nutritional compositions and other bioactive compound specially phenols but pumpkin peels also very excellent dource of antioxidant which also supported by the Hagos et al. 2023 -where Hagos et al. 2023 reported the phenolic compound are almost four times higher the pumpkin seeds in pumpkin peels [32]. The variety of growth circumstances, plant parts, processing, analytical methods and environment,

Formatted: Font: (Default) Times New Roman, 12 pt
Formatted: Font: (Default) Times New Roman, 12 pt

could all be considered in explaining the considerable variances in the phenolic contents [32].

Table 09: Total phenolic compound and antioxidant properties (DPPH and FRAP) of market wheat flour, pumpkin seed powder and pumpkin peel powder sample

Powder Samples	Total Phenolic Compound (mg/100g)	DPPH scavenging activity (m mole/100g)	FRAP Ferric reducing power assay (m mole/100g)
Wheat Flour	83.62±2.85^{b}	122.23±6.86^{c}	279.75±37.12^{c}
Pumpkin seed powder	268.38±1.02^{a}	160.17±14.55^{b}	688.50±10.60^{b}
Pumpkin peel powder	87.82±1.03^{b}	217.52±27.76^{a}	1374.75±15.90^{a}

All values are express as mean ± SD.
Mean followed by different superscript letters in each column are significantly different ($p<0.05$)
~~Allvaluesareexpressasmean±SD.~~
~~Meanfollowedbydifferentsuperscriptlettersineachcolumnaresignificantlydifferent(p<0.05)~~

3.54.2 Total Phenolic Compound and Antioxidant Properties (DPPH scavenging activity and FRAP Ferric reducing power assay) of pumpkin seeds cookies

Table 10 shows the total phenolic compound and antioxidant properties (DPPH and FRAP) of pumpkin seeds cookies. All pumpkin seeds cookies ~~showed~~comparatively showed higher total phenolic compound as compared to control (market wheat flour). This is because of higher total phnolic compound present in pumpkin seeds samples. As a result, pumpkin seeds powders fortification in wheat flour cookies can be excellent substitute of wheat flour to enrich nutrients. It was observed that pumpkin seeds cookies had higher DPPH scavenging activity as compared to control (market wheat flour). On the other hand, FRAP antioxidant properties of pumpkin seeds cookies samples ranged from 1157.25 to 2079.75 m mole/100g. It was also evaluated that pumpkin seeds cookies had higher FRAP antioxidant properties as compared to control (market wheat flour). The higher values of DPPH and FRAP antioxidant activity of pumpkin seeds fortified cookies samples might be due to the presence of higher phenolic compounds in pumpkin seeds.

Table 10: Total phenolic compound and antioxidant properties (DPPH and FRAP) of pumpkin seeds cookies

Formatted: Superscript
Formatted: Superscript
Formatted: Superscript
Formatted: Superscript
Formatted: Superscript
Formatted: Superscript
Formatted: Superscript
Formatted: Superscript
Formatted: Superscript

Cookie Samples	Total Phenolic Compound (mg/100g)	DPPH scavenging activity (m mole/100g)	FRAP Ferric reducing power assay (m mole/100g)
S_A~~A~~	24.51^a±0.57	162.52^{ab}±12.47	1187.25^b±58.33
S_B~~B~~	82.57^a±0.01	117.97^b±18.09	1157.25^b±26.51
S_C~~C~~	~~26~~76.37^a±0.22	196.35^{ab}±39.09	1269.75^b±47.72
S_D~~D~~	71.75^a±2.74	286.64^a±22.46	2079.75^a±79.54
S_E~~E~~	60.28^a±1.14	179.00^{ab}±5.40	1873.50^a±265.1

Values are means of triplicate analysis ± Standard deviation. S_A=Control (100% market wheat flour, Cookie), S_B=95% market wheat flour /5%pumpkin seed cookie, S_C=90% market wheat flour /10%pumpkin seed cookie, S_D=85% market wheat flour /15%pumpkin seed cookie, S_E=80%market wheat flour /20%pumpkin seed cookie)
All values are express as mean ± SD
Mean followed by different superscript letters in each column are significantly different ($p<0.05$)
~~Valuesaremeansoftriplicateanalysis±Standarddeviation.A=Control(100%wheat,Cookie),B=95%wheat/5%pumpkinseedcookie,C=90%wheat/10%pumpkin seed cookie, D=85%wheat/15% pumpkin seed cookie, E=80%wheat/20%pumpkin seed cookie)~~
~~Allvaluesareexpressasmean±SD.~~
~~Meanfollowedbydifferentsuperscriptlettersineachcolumnaresignificantlydifferent(p<0.05)~~

3.~~5~~4.3 Total Phenolic Compound and Antioxidant Properties (DPPH scavenging activity and FRAP Ferric reducing power assay) of pumpkin peels cookies

Table11 represents the total phenolic compound and antioxidant properties (DPPH and FRAP) of pumpkin peels cookies. All pumpkin peels cookies showed higher total phenolic compound as compared to control (market wheat flour). This is because of higher total phenolic compound present in pumpkin peels samples. DPPH scavenging activity of pumpkin peels cookies samples ranged from 111.50 to 199.29 m mole/100g.The highest amount of DPPH properties (199.29 **m mole/100g**) was observed for the cookie sample containing 20% peel powder (sample D). Ferric reducing power (FRAP) assay was evaluated for pumpkin peels cookies which was ranged from 1029.75 to 1187.-25 m mole/100g. It was evaluated that the market wheat flour (control) had the highest (1187.25 mmole/100g) FRAP~~F RAP~~ value.

Table 11: Total phenolic compound and antioxidant properties (DPPH and FRAP) of pumpkin peels cookies

Cookie Samples	Total Phenolic Compound (mg/100g)	DPPH scavenging activity (m mole/100g)	FRAP Ferric reducing power assay (mmole/100g)
P_AA	$24.51^b \pm 0.57$	$162.52^a \pm 12.47$	$1187.25^b \pm 58.33$
P_BB	$80.47^a \pm 2.51$	$138.55^a \pm 13.51$	$1048.50^a \pm 63.63$
P_CC	$46.48^{ab} \pm 1.02$	$111.50^a \pm 7.27$	$1063.50^a \pm 30.75$
P_DD	$60.45^{ab} \pm 5.02$	$199.294^a \pm 25.37$	$1029.75^a \pm 5.30$

Values are means of triplicate analysis ± Standard deviation. P_A=Control (100% wheat, Cookie), P_B=95%wheat/5%pumpkin peel cookie, P_C=90%wheat/10%pumpkin peel cookie, P_D=80%wheat/20%pumpkin peel cookie)
All values are express as mean ± SD.
Mean followed by different superscript letters in each column are significantly different ($p<0.05$)
~~Valuesaremeansoftriplicateanalysis±Standarddeviation.A=Control(100%wheat, Cookie),B=95%wheat/5%pumpkinpeel cookie,C=90%wheat/10%pumpkin peel cookie, D=80%wheat/20%pumpkin peel cookie)~~
~~Allvaluesareexpressasmean±SD.~~
~~Meanfollowedbydifferentsuperscriptlettersineachcolumnaresignificantlydifferent(p<0.05)~~

3.54.4 Total Phenolic Compound and Antioxidant Properties (DPPH) of noodles samples

Table 12 displayed total phenolic compound of all noodles sample ranged from 9.55 to 34.96 mg/100g. Sample N_B had the highest total phenolic compound (34.96mg/100g). On the other hand, DPPH scavenging activity of all noodles sample ranged from 14.03 to 27.08 m mole/100g. Sample N_C had the highest DPPH scavenging activity (27.08 m mole/100g). All noodles samples had higher DPPH scavenging activity than control. It indicated that wheat flour can be fortified with pumpkin seeds and peels powders to enrich nutritional properties specially antioxidant activities.

Table 12: Total Phenolic Compound and Antioxidant Properties (DPPH) ~~**TotalphenoliccompoundandDPPHantioxidantproperties**~~ **of noodles samples.**

Noodles Samples	Total Phenolic Compound (mg/100g)	DPPH scavenging ~~activity~~ activity (m mole/100g)
N_AA	18.86 ± 0.49^{ab}	14.03 ± 2.54^b
N_BB	34.96 ± 0.27^a	16.28 ± 1.79^b
N_CC	21.82 ± 1.09^{ab}	27.08 ± 1.27^a
N_DD	9.55 ± 1.09^b	18.75 ± 1.42^{ab}

Values are means of triplicate analysis ± Standard deviation. N_A=Control (100% wheat flour noodles), N_B=90%wheat/10% pumpkin seeds noodles, N_C=90%wheat/10% pumpkin peels noodles, N_D=80%wheat/10%pumpkin seeds and 10% pumpkin peels noodles
All values are express as mean ± SD.

Formatted: Subscript

Formatted: Subscript

Mean followed by different superscript letters in each column are significantly different ($p<0.05$)
Values are meansof triplicate analysis ± Standard deviation. A=Control (100% wheat flournoodles), B=90%wheat/10% pumpkin seedsnoodles,C=90%wheat/10% pumpkin peels noodles, D=80%wheat/10%pumpkin seeds and 10% pumpkin peels noodles)
Allvaluesareexpressasmean±SD.
Meanfollowedbydifferentsuperscriptlettersineachcolumnaresignificantlydifferent(p<0.05)

3.6 5 Hydration properties of powder samples:

Hydration properties such as water absorption index, water solubility index and swelling capacity of market wheat flour, pumpkin seed powder and pumpkin peel powder are shown in Table 13. The values of all powder samples ranged from 187.40 to 764.60 g/100g, 5.60 to 17.60 ml/100g and 5.93 to 21.37ml/100g for water absorption index, water solubility index and swelling capacity, respectively. Hussain et al. (2023) reported 60.31/100g and 60.98/100g water absorption index of the pumkin peels powder and pumpkin seeds powder [38]. The data showed pumpkin peels powder and seeds powder had hydration properties which is close to the market wheat flour (control) sample. It indicates that these two-powder ingredients (seeds powder and peels powder) can be partially replaced by wheat flour for making less gluten cookie. It is impossible completely reduced the gluten by the seeds and peels powder but we can enrich the noodles nutrition value by partially adding the waste materials of pumpkin.

Table 13: Hydration Properties (water absorption index, water solubility index and swelling capacity of powder samples)

Cookie Samples	Water Absorption Index (g/100g)	Water Solubility Index (ml/100g)	Swelling Capacity (ml/100g)
Wheat Flour	764.60±20.64^a	17.60±1.13^a	21.37±1.66^a
Pumpkin seed powder	240.40±6.70^b	11.20±0.56^b	9.53±0.71^b
Pumpkin peel powder	187.40±0.20^b	5.60±0.01^c	5.93±0.01^c

All values are express as mean ± SD.
Mean followed by different superscript letters in each column are significantly different ($p<0.05$)
Allvaluesareexpressasmean±SD.
Meanfollowedbydifferentsuperscriptlettersineachcolumnaresignificantlydifferent(p<0.05)

3.7 6 Sensory evaluation

Twelve Ten judgesjudges' evaluated cookies and noodles made with different ratios of pumpkin powder based on texture, aroma, color, taste, and overall acceptability. Tables 13, 14, and 15 present mean scores for each criterion. Two-way ANOVA for color preference indicated significant differences

($p<0.05$) among the samples. However, DMRT showed no significant taste differences in pumpkin peel cookies. Among peel cookies, sample P_CS3 scored highest in color, aroma, texture, and overall acceptability. In pumpkin seed cookies, sample S_DS4 with 15% powder achieved the highest scores. For noodles, sample S3P_C with 10% pumpkin peel powder outperformed others, securing the highest scores in color, texture, aroma, taste, and overall acceptability. The findings suggest optimal ratios for enhancing sensory attributes in pumpkin-enriched cookies and noodles.

The sensory characteristics of cookies containing varying levels of pumpkin seeds are detailed in Table 15. The results of sensory evaluation reveal significant variations among different cookie products. Notably, Sample PC (90% wheat/10% pumpkin peel cookie) achieved the highest ratings in color, aroma, taste, texture, and overall acceptability, scoring 8, 7.33, 7.67, 8, and 8.33, respectively, as illustrated in Table 14. Similarly, Sample SD (85% market wheat flour/15% pumpkin seed cookie) demonstrated superior attributes, scoring highest in color, aroma, taste, texture, and overall acceptability, with ratings of 7.50, 7.67, 8, 7.67, and 8.33, respectively, as shown in Table 15. Additionally, in Table 16, NC (90% wheat/10% pumpkin peels noodles) emerged with the highest score among others.

The enhancement in texture upon increasing pumpkin seeds in cookies may be attributed to their high protein content, as observed by Atuonwu and Akobundu (2010). The color score ranged from 6.33 in control cookies to 7.50 in cookies containing 20% pumpkin seeds, reflecting the desirable effect of pumpkin seeds' high protein content. Furthermore, the gradual decrease in the color score of cookies may be attributed to sugar caramelization and Maillard reactions between sugars and amino acids, consistent with findings by Alshehry et al. (2020) indicating an improvement in color score with higher supplementation levels [33, 34].

Cookies exhibited significant variations in flavor, with the highest score observed in those containing 15% pumpkin seeds and the lowest in control cookies. This increase in flavor was also noted by Atuonwu and Akobundu (2010) with higher levels of pumpkin seeds. Moreover, the highest taste score was found in products containing 15% pumpkin seeds, emphasizing the importance of pumpkin seed inclusion for developing taste characteristics in cookies (Murkovic, 2004) [33, 36].

Studies by Alshehry (2020) and Kanwal et al. (2015) demonstrated successful incorporation of pumpkin seed flour as a partial replacement for wheat flour, resulting in cookies with enhanced nutritional value and wholesomeness without compromising overall acceptability [34, 35].

Table 14: Mean score for texture, aroma, color, taste and overall acceptability of Pumpkin peel cookies.

Cookie's type	Color	Aroma	Taste	Texture	Overall acceptability
P_ASampleS$_1$	7.67±0.99^{a}	7.00±0.85^{ab}	7.00±0.86^{a}	7.33±0.98^{a}	7.33±1.31^{b}
P_BSampleS$_2$	7.67±0.49^{a}	6.58±0.51^{bc}	6.91±0.80^{a}	7.33±1.30^{a}	7.66±0.98^{ab}

P_C ~~SampleS$_3$~~	8.00±0.86^a	7.33±0.49^a	7.67±0.99^a	8.00±0.85^a	8.330.49±^{a}
P_D ~~SampleS$_4$~~	6.67±0.99^b	6.33±0.50^c	7.17±0.84^a	6.33±0.50^b	6.00±0.01^c

Values are means of triplicate analysis ± Standard deviation. P_A=Control (100% wheat, Cookie), P_B=95%wheat/5%pumpkin peel cookie, P_C=90%wheat/10%pumpkin peel cookie, P_D=80%wheat/20%pumpkin peel cookie)
All values are express as mean ± SD.
Mean followed by different superscript letters in each column are significantly different ($p<0.05$)
~~Allvaluesareexpressasmean;Meanfollowedbydifferentsuperscriptlettersineachcolumnaresignificantlydifferent (p<0.05)~~
~~Sample S_1=Control (Market Flour);Sample S_2=05%pumpkinpeel powder; Sample S_3=10%pumpkinpeelpowder; SampleS$_4$= 20% pumpkinpeelpowder~~

Table 15: Mean score for texture, aroma, color, taste and overall acceptability of Pumpkin seed cookies.

Cookie's type	Color	Aroma	Taste	Texture	Overall acceptability
S_A ~~SampleS$_1$~~	6.42±0.51^b	7.33±1.30^{ab}	7.00±0.85^b	7.33±0.98^a	7.33±1.30^b
S_B ~~SampleS$_2$~~	6.58±0.50^b	6.67±0.49^b	6.33±0.98^b	6.33±0.49^b	7.33±1.30^b
S_C ~~SampleS$_3$~~	6.33±0.77^b	6.67±0.98^b	6.67±0.49^b	7.00±0.85^{ab}	7.00±0.85^b
S_D ~~SampleS$_4$~~	7.50±0.67^a	7.67±0.98^a	8.00±0.85^a	7.67±0.78^a	8.33±0.49^a
S_E ~~SampleS$_5$~~	6.58±0.51^b	7.00±0.96^{ab}	6.67±0.50^b	7.00±0.85^{ab}	7.33±1.32^b

Values are means of triplicate analysis ± Standard deviation. S_A=Control (100% market wheat flour, Cookie), S_B=95% market wheat flour /5%pumpkin seed cookie, S_C=90% market wheat flour /10%pumpkin seed cookie, S_D=85% market wheat flour /15%pumpkin seed cookie, S_E=80%market wheat flour /20%pumpkin seed cookie)
All values are express as mean ± SD
Mean followed by different superscript letters in each column are significantly different ($p<0.05$)
~~Allvaluesareexpressasmean;Meanfollowedbydifferentsuperscriptlettersineachcolumnaresignificantlydifferent(p<0.05)~~
~~SampleS$_1$=Control(MarketFlour);SampleS$_2$=05%pumpkinseedpowder;SampleS$_3$=10%pumpkinseedpowder;SampleS$_4$=15%pumpkinseed powder; Sample S_5 = 20% pumpkin seed powder~~

Table 16 Mean score for texture, aroma, color, taste and overall acceptability of Pumpkin seed and peel noodles.

Formatted: Superscript

Noodles type	Color	Aroma	Taste	Texture	Overall acceptability
N_A SampleS$_1$	7.67±0.98^{a}	7.25±1.21^{a}	7.00±0.84^{a}	7.33±0.98^{a}	7.33±1.31^{bc}
N_B SampleS$_2$	7.67±0.49^{a}	7.33±0.50^{a}	7.00±0.95^{a}	7.33±1.30^{a}	7.67±0.99^{ab}
N_C SampleS$_3$	8.00±0.85^{a}	7.67±0.65^{a}	7.67±0.99^{a}	8.00±0.85^{a}	8.33±0.50^{a}
N_D SampleS$_4$	6.67±0.98^{b}	6.33±0.49^{b}	6.00±0.01^{b}	6.33±0.49^{b}	6.58±0.58^{c}

Values are means of triplicate analysis ± Standard deviation. N_A=Control (100% wheat flour noodles), N_B=90%wheat/10% pumpkin seeds noodles, N_C=90%wheat/10% pumpkin peels noodles, N_D=80%wheat/10%pumpkin seeds and 10% pumpkin peels noodles
All values are express as mean ± SD.
Mean followed by different superscript letters in each column are significantly different ($p<0.05$)
Allvaluesareexpressasmean;Meanfollowedbydifferentsuperscriptlettersineachcolumnaresignificantlydifferent ($p<0.05$)SampleS$_1$=Control(MarketFlour);SampleS$_2$=10%pumpkinseedpowdernoodles;SampleS$_3$=10%pumpkinpeelpowdernoodles;SampleS$_4$ = 10% pumpkin seed powder + 10% pumpkin peel powder noodles

Formatted: Superscript

Formatted: Superscript

Minerals content

Table 17 outlines mineral content variations in different noodle samples, with significant differences observed in phosphorus, calcium, magnesium, zinc, and iron levels. Notably, 10% pumpkin seed powder noodles exhibited the highest calcium content, while pumpkin peel powder noodles had elevated magnesium concentrations. Zinc and iron levels were notably higher in samples with pumpkin seed and peel powders. These findings underscore the potential of these additives to enhance noodle nutrition. The study suggests that adjusting pumpkin-derived ingredient types and concentrations can optimize mineral enrichment, making these noodles a nutritious alternative to traditional counterparts. The research provides valuable insights into the mineral content of pumpkin-enriched noodles, encouraging further exploration of formulations for optimal nutritional benefits in staple foods.

Table17: Meanscoreformineral contentofPumpkinseedand peel noodles.

Sample	P (mg/kg)	Ca (mg/kg)	Mg (mg/kg)	Zn (mg/kg)	Fe (mg/kg)
S1	309.5±0.990^{b}	7050.25±3.748^{a}	7176.7±2.687^{a}	37.82±1.329^{b}	115.7±1.216^{a}
S2	686.7±5.515^{ab}	4953.9±6.081^{b}	4958±1.131^{b}	55.62±1.329^{ab}	307.915±1.096^{a}
S3	444.1±5.798^{ab}	6025.6±3.960^{ab}	6135±21.213^{ab}	39.88±0.509^{b}	78.94±1.216^{a}
S4	816.5±2.970^{a}	5690.5±3.253^{ab}	5494±5.091^{ab}	68.64±0.735^{a}	131.61±0.778^{a}

Formatted Table

Allvaluesareexpressasmean;Meanfollowedbydifferentsuperscriptlettersineachcolumnaresignificantlydifferent ($p<0.05$)SampleS$_1$=Control(MarketFlour);SampleS$_2$=10%pumpkinseedpowdernoodles;SampleS$_3$=10%pumpkinpeelpowdernoodles;SampleS$_4$ = 10% pumpkin seed powder + 10% pumpkin peel powder noodles

4. Conclusions

Enriching confectionery products with pumpkin seeds and peels enhances their nutritional value. This process also ensures that the products have satisfactory technological and sensory characteristics. ~~Theproductionofapumpkinseedsand peelsenrichedmakesanykindsof confectionary product morenutritiouswhichwehaveenoughsatisfactorytechnologicalandsensorycharacteristics.~~The effects of seeds and peels on the physicochemical and nutritional characteristics of different confectionary products such as cookies and noodles were investigated. Pumpkin seed and peel powders could be used not only enrich nutritious value but also to enhance the quality of food products ~~texture~~ antioxidant properties such as total phenolic compounds where all pumpkin peels sample showed the enrichment of the confectionary products~~and other rheological properties such as color and flavor~~. The increase in fibre and ash content in the cookies and noodles showed qualitative and quantitative advantages when compared to the market flour. The sensory acceptability rate reached 78%, which refers to "Good" in the hedonic rating scale, which is considered good acceptability. The results demonstrate production viability for this alternative food due to its very low cost and easy to preparation but more importantly, it can be destined to different meals because it can improve the nutritional life.

References

[1] Norfezah, M.N., Hardacre, A. and Brennan, C.S., 2011. Comparison of waste pumpkin material and its potential use in extruded snack foods. Food Science and Technology International, 17(4), pp.367-373.

[2] Statistics, B.B.O., ~~2011~~2021. Statistical yearbook of Bangladesh. Statistics Division, Ministry of Planning, Dhaka, Government of the People's Republic of Bangladesh.

[3] El-Adawy, T.A. and Taha, K.M., 2001. Characteristics and composition of watermelon, pumpkin, and paprika seed oils and flours. Journal of agricultural and food chemistry, 49(3), pp.1253-1259.

[4] Brennan, C.S. and Samyue, E., 2004. Evaluation of starch degradation and textural characteristics of dietary fiber enriched biscuits. International Journal of Food Properties, 7(3), pp.647-657.

[5] Fagan, C.C., O'Donnell, C.P., Cullen, P.J. and Brennan, C.S., 2006. The effect of dietary fibre inclusion on milk coagulation kinetics. Journal of Food Engineering, 77(2), pp.261-268.

[6] Tudorica, C.M., Jones, T.E.R., Kuri, V. and Brennan, C.S., 2004. The effects of refined barley β- glucan on the physico- structural properties of low- fat dairy products: curd yield, microstructure, texture and rheology. Journal of the Science of Food and Agriculture, 84(10), pp.1159-1169.

[7] Malkanthi, H.H.A. and Umadevi, S.H., 2018. Effect of dried pumpkin pulp and seed powder on physical, chemical and sensory properties of biscuits. Int J Sci Res, 7, pp.32-4.

[8] Kim, M.Y., Kim, E.J., Kim, Y.N., Choi, C. and Lee, B.H., 2012. Comparison of the chemical compositions and nutritive values of various pumpkin (Cucurbitaceae) species and parts. Nutrition research and practice, 6(1), pp.21-27.

[9] Adams, G.G., Imran, S., Wang, S., Mohammad, A., Kok, S., Gray, D.A., Channell, G.A., Morris, G.A. and Harding, S.E., 2011. The hypoglycaemic effect of pumpkins as anti-diabetic and functional medicines. Food Research International, 44(4), pp.862-867.

[10] Yadav, M., Jain, S., Tomar, R., Prasad, G.B.K.S. and Yadav, H., 2010. Medicinal and biological potential of pumpkin: an updated review. Nutrition research reviews, 23(2), pp.184-190.

[11] AOAC (1998). Official Methods of Analysis of AOAC International (14^{th}ed.). Washington, DC, USA: Association of Official AnalyticalChemistry. Methods, 950.46, 938.08, 960.39, 955.04

[12] Navacchi, M.F.P., de Carvalho, J.C.M., Takeuchi, K.P. and Danesi, E.D.G., 2012. Development of cassava cake enriched with its own bran and Spirulinaplatensis. ActaScientiarum.Technology, 34(4), pp.465-472.

[13] Akter, M.S., Ahmed, M. and Eun, J.B., 2010. Solvent effects on antioxidant properties of persimmon (Diospyros kaki L. cv. Daebong) seeds. International journal of food science & technology, 45(11), pp.2258-

2264.

[14] Mestres, C., Colonna, P. and Buléon, A., 1988. Characteristics of starch networks within rice flour noodles and mungbean starch vermicelli. Journal of food science, 53(6), pp.1809-1812.

[15] Hussain, A., Kausar, T., Sehar, S., Sarwar, A., Ashraf, A.H., Jamil, M.A., Noreen, S., Rafique, A., Iftikhar, K., Quddoos, M.Y. and Aslam, J., 2022. A Comprehensive review of functional ingredients, especially bioactive compounds present in pumpkin peel, flesh and seeds, and their health benefits. Food Chemistry Advances, p.100067.

[16] Dhiman, A.K., Bavita, K., Attri, S. and Ramachandran, P., 2018. Preparation of pumpkin powder and pumpkin seed kernel powder for supplementation in weaning mix and cookies. International Journal of Chemical Studies, 6(5), pp.167-175.

[17] Syam, A., Zaenal, Y.K., Aulia, N.A., Wati, I.P. and Mansur, M.A., 2019. Development and biochemical analysis of pumpkin seed (CucurbitamoschataDurch) biscuits. Pak J Nutr, 18(8), pp.743-6.

[18] Mishra, S. and Sharma, K., 2019. Development of pumpkin peel cookies and its nutritional composition. Journal of Pharmacognosy and Phytochemistry, 8(4), pp.370-372.

[19] Saleh, S.M. and Ali, S., 2020. Physico-functional properties, nutritional quality, and sensory characteristics of pumpkin peel puree fortified biscuit. Egyptian Journal of Food Science, 48(2), pp.203-212.

[20] Von Lintig, J., 2010. Colors with functions: elucidating the biochemical and molecular basis of carotenoid metabolism. Annual review of nutrition, 30, pp.35-56.

[21] Shami, N.J.I.E. and Moreira, E.A.M., 2004. Lycopene as an antioxidant agent. Revista de nutrição, 17(2), pp.227-236.

[22] Saura-Calixto, F. and Goñi, I., 2006. Antioxidant capacity of the Spanish Mediterranean diet. Food chemistry, 94(3), pp.442-447.

[23] Pierson, J.T., Dietzgen, R.G., Shaw, P.N., Roberts- Thomson, S.J., Monteith, G.R. and Gidley, M.J., 2012. Major Australian tropical fruits biodiversity: Bioactive compounds and their bioactivities. Molecular nutrition & food research, 56(3), pp.357-387.

[24] Lee, C.H., Cho, J.K., Lee, S.J., Koh, W., Park, W. and Kim, C.H., 2002. Enhancing β- carotene content in Asian noodles by adding pumpkin powder. Cereal chemistry, 79(4), pp.593-595.

[25] Godoy, H.T. and Rodriguez-Amaya, D.B., 1998. Occurrence of cis isomers of provitaminsA in Brazilian vegetables. Journal of Agricultural and Food Chemistry, 46(8), pp.3081-3086.

[26] Maria do Socorro, M.R., Alves, R.E., de Brito, E.S., Pérez-Jiménez, J., Saura-Calixto, F. and Mancini-Filho, J., 2010. Bioactive compounds and antioxidant capacities of 18 non-traditional tropical fruits from Brazil. Food chemistry, 121(4), pp.996-1002.

[27] Park, W., Shelton, D.R., Peterson, C.J., Wehling, R.L. and Kachman, S.D., 1997. Evaluation of Korean raw noodle (SaengMyon) color and cooking properties among hard red winter and hard white wheat samples. Food

Science and Biotechnology, 6(1), pp.20-25.

[28] Yap, E.S.P., Uthairatanakij, A., Laohakunjit, N. and Jitareerat, P., 2022. Influence of hot air drying on capsaicinoids, phenolics, flavonoids and antioxidant activities of 'Super Hot'chilies. PeerJ, 10, p.e13423.

[29] Ghigo, G., Maranzana, A., Causà, M. and Tonachini, G., 2007. Theoretical mechanistic studies on oxidation reactions of some saturated and unsaturated organic molecules. *Theoretical Chemistry Accounts*, *117*, pp.699-707.

Formatted: Font: (Default) Times New Roman, 12 pt

[30] Taneya, M.L.J., Biswas, M.M.H. and Shams-Ud-Din, M., 2014. The studies on the preparation of instant noodles from wheat flour supplementing withs weet potato flour. *Journal of the Bangladesh Agricultural University*, *12*(1), pp.135-142.

Formatted: Font: (Default) Times New Roman, 12 pt

[31] Saeid, A., Hoque, S., Kumar, U., Das, M., Muhammad, N., Rahman, M.M. and Ahmed, M., 2015. Comparative studies on nutritional quality of commercial wheat flour in Bangladesh. *Bangladesh Journal of Scientific and Industrial Research*, *50*(3), pp.181-188.

Formatted: Font: (Default) Times New Roman, 12 pt

[32] Hagos, M., Chandravanshi, B.S., Redi-Abshiro, M. and Yaya, E.E., 2023. Determination of total phenolic, total flavonoid, ascorbic acid contents and antioxidant activity of pumpkin flesh, peel and seeds. *Bulletin of the Chemical Society of Ethiopia*, *37*(5), pp.1093-1108.

Formatted: Font: (Default) Times New Roman, 12 pt

[33] Atuonwu AC, Akobundu A., 2010. Nutritional and Sensory Guality of Cookies Supplemented with Defatted Pumpkin (Cucurbita pepo) Seed Flour. *Pakistan Journal of Nutrition*, *9*(7), pp.672-677.

[34] Alshehry, G.A., 2020. Preparation and nutritional properties of cookies from the partial replacement of wheat flour using pumpkin seeds powder. *World*, *9*(2), pp.48-56.

[35] Kanwal, S., Raza, S., Naseem, K., Amjad, M., Bibi, N. and Gillani, M., 2015. Development, physico-chemical and sensory properties of biscuits supplemented with pumpkin seeds to combat childhood malnutrition in Pakistan. *Pakistan Journal of Agricultural Research*, *28*(4).

[36] Murkovic, M., Piironen, V., Lampi, A.M., Kraushofer, T. and Sontag, G., 2004. Changes in chemical composition of pumpkin seeds during the roasting process for production of pumpkin seed oil (Part 1: non-volatile compounds). *Food chemistry*, *84*(3), pp.359-365.

[37] Apostol, L., Moşoiu, C.L.A.U.D.I.A., Iorga, C.S. and Martínez, S.Á., 2020. Effect of the addition of pumpkin powder on the physicochemical qualities and rheological properties of wheat flour. *Romanian Biotechnological Letters*, *25*(3), pp.1594-1600.

Formatted: Font: (Default) Times New Roman, 12 pt

[38] Hussain, A., Kausar, T., Aslam, J., Quddoos, M.Y., Ali, A., Kauser, S., Zerlasht, M., Rafique, A., Noreen, S., Iftikhar, K. and Waheed Iqbal, M., 2023. Physical and rheological studies of biscuits developed with different replacement levels of pumpkin (Cucurbita Maxima) Peel, flesh, and seed powders. *Journal of Food Quality*, *2023*.

Formatted: Font: (Default) Times New Roman, 12 pt

[39] Stevenson, D.G., Eller, F.J., Wang, L., Jane, J.L., Wang, T. and Inglett, G.E., 2007. Oil and tocopherol content and composition of pumpkin seed oil in 12 cultivars. *Journal of agricultural and food chemistry*, *55*(10), pp.4005-4013.

[40] Al Zuhair, H.A.N.A., Abd El-Fattah, A.A. and El-Sayed, M.I., 2000. Pumpkin-seed oil modulates the effect of felodipine and captopril in spontaneously hypertensive rats. *Pharmacological Research*, *41*(5), pp.555-563.

[41] Jian, L., Du, C.J., Lee, A.H. and Binns, C.W., 2005. Do dietary lycopene and other carotenoids protect against prostate cancer?. *International journal of cancer*, *113*(6), pp.1010-1014.

Detailed response to reviewers

Manuscript Reference: PONE-D- 23-44103

Manuscript Title: Enhancing Nutritional Value and Quality of Cookies through Pumpkin Peel and Seed Powder Fortification.

Author(s): Md. Asaduzzaman[1,2], Mahbubur Rahman[1], Mst. Sorifa Akter[3*]

1) Department of Food Engineering and Technology, State University of Bangladesh, Dhaka, Bangladesh.
2) Department of Food Processing and Preservation, Hajee Mohammad Danesh Science and Technology University, Dinajpur, Bangladesh
3) Department of nutrition and food Engineering, Daffodil International University, Dhaka, Bangladesh.

*Corresponding Author

Dear Editor and Reviewers,

Thank you very much for your constructive and useful suggestions. Those comments were valuable and very helpful for revising and improving our paper, aswell as for increasing the significance of our research. The responses to editor andreviewer's comments/suggestions of our manuscript have been answered carefullypoint-by-point as follows. Some minor mistakes within the manuscript have alsobeen corrected.

Note: All the line numbers in our responses refer to the revised manuscript. Thered color in the manuscript indicates the changes made during revision.

Response to reviewers

Reviewer #1:

Thank you for your positive perspective on the manuscript.

The comments of the reviewer

Title:
Is it really going to be "A Wheat Flour Replacement Approach"? It's tough to say with such a low amount of seeds and peel powder. Besides, pumpkin seeds are very small amounts, considering the massive amount of wheat flour produced worldwide. I suggest deleting this portion: "A Wheat Flour Replacement Approach."

Comments of the answer:

According to the suggestion of the reviewer, "A Wheat Flour Replacement Approach" is deleting from the title.

Abstract:
-"Significant differences ($p<0.05$) in protein, fibre, beta……..''---This statement is not clear. For this sentence, we do not know if the amounts are higher or lower or if they have a positive or negative impact on product quality. Please make it clear.

As you mentioned % of peel, seed, etc., in the results and conclusion of the abstract, you should add this to the methodology, too.

You have also mentioned Overall acceptability in the results of the abstract but did not mention it in the methods of the abstract.

Comments of the answer:

The abstract is rewritten according to the suggestion of the reviewer 01. The methodology is added in the abstract.

Introduction:
-Use updated data. You used data from 2005, and now it's 2024!

-The introduction section is very short; it lacks literature study and references. The novelty of the work has not been stated properly.

Comments of the answer:

The updated data added in the introduction section and data are updated. There more literature and references added in the introduction.

Material and Methods:

-Section 2.2: Were the seeds husked before milling?

Comments of the answer: we did not husked the seeds before milling, we add the husked to in the cookies and noodles development.

-Using both Figures 1 and 2 is unnecessary. You can merge them and mention the seed/peel portion in different samples. Otherwise, you may discard both of the figures and illustrate them in your writing.

Comments of the answer: While it is true that both figures share many commonalities, combining them would result in overly complicated design and complexity in understanding. Hence, we have chosen to maintain them as separate figures. However, should you suggest amalgamating them into one figure; we will provide detailed representation of the leveling process in the methodology section.

The samples in Figures 1 to 3 originally had disparate labeling, which has now been standardized uniformly under the same notation.

S_A = Control (100% market wheat flour, Cookie)

S_B = 95% market wheat flour /5%pumpkin seed cookie

S_C = 90% market wheat flour /10%pumpkin seed cookie

S_D = 85% market wheat flour /15%pumpkin seed cookie

S_E = 80%market wheat flour /20%pumpkin seed cookie

P_A = Control (100% market wheat flour, Cookie)

P_B = 95%wheat/5%pumpkin peel cookie

P_C = 90%wheat/10%pumpkin peel cookie

P_D = 80%wheat/20%pumpkin peel cookie

N_A = Control (100% wheat flour noodles)

N_B = 90%wheat/10% pumpkin seeds noodles

N_C = 90%wheat/10% pumpkin peels noodles

N_D = 80%wheat/10%pumpkin seeds and 10% pumpkin peels noodles

-Section 2.4 and 2.5: Where does sweet potato powder come here?

Comments of the answer: Mistakenly we wrote “sweet potato peel” which corrected to pumpkin seeds powder, peels powders, cookies and noodles in the section 2.4 and 2.5.

-Section 2.4: If the Protein determination process is through the Kjeidhal method, please mention the N conversion factor.

Comments of the answer: In the section 2.4 – the N conversion factor is added.

-Section 2.5-2.9: You must have to add all the details about the methodology. These methods are not reproducible and not suitable for publication.

Comments of the answer: according to your suggestion all section 2.5 to 2.8 are described. One section is added to the previous section, so that, one section is minimized.

-Section 2.10: The sensory analysis should be vividly described. Also, the number of panellists needs to be higher. Add whether they were trained/semi-trained, how samples were presented to them, etc. The statistical analysis portion should be moved to section 2.11.

Comments of the answer: The sensory analysis methodology has been updated in accordance with the reviewer's suggestions. The statistical analysis section has been relocated to Section 2.10. All panelists are faculty members of the Department of Food Engineering and Technology, and prior to the sensory analysis, they underwent training for the tests. I regret the limited number of panelists; however, currently, our department lacks qualified sensory panelists. We are actively working to increase the number of panelists and are organizing training sessions to recruit and train new members.

-Section 2.11: Mention how many replicates for each observation were used.

Comments of the answer: All evaluations of samples were all done in triplicate.

Results and Discussion:

-Table 01: Check 3.9.1±0.48b?

Comments of the answer: the data was corrected. It will be 3.91 ± 0.48^{b}

-In methodology, you mentioned Sample 1, 2, 3, etc…in Results and Discussion, you are saying Sample A, B, C, etc…

Comments of the answer: The sample indications were wrong which was corrected and described according to the data in the methodology section.

-If every result of your study is comparable to those of Syam et al. (2019), then what is the justification of your study?

Comments of the answer: Along with syam et al. (2019), more literature added in the section 3.1.2. such as Alshehry (2020) and Apostol et al. (2020)

-Where is the Carbohydrate content in your results???

Comments of the answer: There are no carbohydrate discussions in this study, so the carbohydrate portion was removed from methodology.

-Section 3.2: "The antioxidant capacities of leaves????????? may vary………..."? You should be careful while writing a manuscript for publication.

Comments of the answer: thank you the suggestion, we will be more careful to write the manuscript for publishing in this renowned journal.

-Section 3.3: Add the units of cooking loss and cooking yield. From the standard deviation, it seems the indications of significant differences are not correct.

Comments of the answer: We add more literature in this section to differentiate differences.

-Table 12: The results of sample D are not homogenous with the other results. Besides, where are the FRAP results of Noodles?

Comments of the answer: The description is corrected and due to the some difficulties in the laboratory the FRAP test are not include for the noodles sample.

-Section 3.6: You should mention the significance of Hydration properties. The discussion of this section is also inadequate. Like other parameters, you didn't measure the hydration properties of your samples.

Comments of the answer: in this section firstly we discussed the importance of the hydration properties and then we add more discussion and literatures.

-Section 3.7: Previously, you mentioned 10 panellists!

Comments of the answer: Mistakenly we count our self as a panelist which was corrected.

-Table 14, 15, 16: You need to add the standard deviations in the results.

-Besides, this section lacks discussion.

Comments of the answer: The standard deviation is added in table 14 to 16. Description and literature is also added.

-Minerals content: You did not mention this in the methodology. If you would like to keep it, you should add it before sensory evaluation. Obviously, you should add mineral contents to all samples and ingredients as well. You should improve the discussion section also.

Comments of the answer: The section on mineral content has been removed because no other confectionery items besides noodles and samples were examined for minerals, which is not relevant to this study.

Conclusions:
-The first sentence is not clear. Make it simple.

Comments of the answer: The conclusion sentence has been simplified for brevity. The antioxidant activity is discussed in the corresponding section.

References:
Most of the references used in the manuscripts are too old. You should use more updated references.

Comments of the answer: In the references section, updated refinances are added which cited in the study.

Reviewer #2:

P11 2.1.the variety and degree of ripeness should be mentioned. The city of Dhaka should also be mentioned with the country. The wheat flour used should be characterised more precisely, gliadin, protein, starch content or technological property should be mentioned.

Comments of the answer:

In Section 2.1, in compliance with the reviewer's suggestion, Dhaka is now accompanied by the country name in the author's affiliation and the materials and methods section. Additionally, the gluten value was included in the manuscript following the powder test as per the reviewer's recommendation. Furthermore, the scientific name of the pumpkin variety and the degree of ripeness are detailed in the materials and methods section.

Figures 1-3 have different labelling of the samples, there are mixed samples1-4, formula1-4 and in the results are discus samples A-B. This should be standardised. This figure also shows the abbreviation gm, this should be explained in the note, or if it is g, it should be changed.

Comments of the answer:

Figure 1 to 3 had different labeling of the samples which now converted in to same notation and standardised uniformly where

S_A = Control (100% market wheat flour, Cookie)

S_B = 95% market wheat flour /5%pumpkin seed cookie

S_C = 90% market wheat flour /10%pumpkin seed cookie

S_D = 85% market wheat flour /15%pumpkin seed cookie

S_E = 80%market wheat flour /20%pumpkin seed cookie

P_A = Control (100% market wheat flour, Cookie)

P_B = 95%wheat/5%pumpkin peel cookie

P_C = 90%wheat/10%pumpkin peel cookie

P_D = 80%wheat/20%pumpkin peel cookie

N_A = Control (100% wheat flour noodles)

N_B = 90%wheat/10% pumpkin seeds noodles

N_C = 90%wheat/10% pumpkin peels noodles

N_D = 80%wheat/10%pumpkin seeds and 10% pumpkin peels noodles

P13 Figure 2 the weight of the ingredients should be stated separately "Water & eggs 33g".

Comments of the answer: In the figure 02, the weight of the ingredients mistakenly wrote water and egg 33g. It should be only egg 33 g which is corrected in the figure.

P15 2.4 and 25 why is it written "sweet potato peel"?

Comments of the answer: Mistakenly we wrote “sweet potato peel” which corrected to pumpkin seeds powder, peels powders, cookies and noodles in the section 2.4 and 2.5.

P16 For used equipment the manufacturer and country of origin should be stated. The source of the chemical used should also be mentioned.

Comments of the answer: The equipment manufacturer and origin are added in the 2.2 section including country. The source of the chemical used in the study also included in the section 2.1.

P17 Table 1 shows no statistical differences between the samples, but the mean is different and the SD is low. The author should explain this.

Comments of the answer: The statistical differences between the samples are corrected and the explanation is added in the section 3.1.1.

P18 biscuits33.05/100g - missing space

Comments of the answer: the space is added between biscuits 33.05/100g in the section 3.1.2.

P18 2nd column (C) and P19 3C - the author should explain how fat oxidation can change fat content or delete this sentence.

Comments of the answer: The explanation of fat oxidation is added in the section 3.1.1. This also repeated in the section 3.1.2.

P18 "Dietary fibre values were significantly different between the cookies and the highest was found in sample D (1.61/100g), while the lowest content (0.32/100g) was investigated in sample A. This observation was comparable to that of Syam et al. (2019) who reported a fat value of 1.64/100g in pumpkin seed cookies [17]." - Correctly described ingredients

Comments of the answer: The sample indications were wrong which was corrected and described according to the data.

P21 Chapter 3.2 should be part of 3.1 as in previous biscuits.

Comments of the answer: chapter 3.2 is changed to 3.1.5

P22 Table 05 sample labelling should be identical in manuscript,

Comments of the answer: Table 05 sample labeling is corrected and wrote according to the formulation figure and data.

P23 Table 06 use the same orientation in the cell, for Delta E there are no significant differences but the values are different and the SD is low, this should be explained.

Comments of the answer: in the table 06, the leveling data is corrected and the explanations are added in the section 3.3.4. More literature is added describe in the section.

P24 1C I did not agree that seeds are a better source of polyphenols. This should be confirmed by literature or discussed in more detail. In my opinion there are interferences with proteins in seeds.

Comments of the answer: the pumpkin peels are more better source of the total phenolic compounds then pumpkin seeds which was discussed in the section 3.4.1

P24 Table 09 were there no statistical differences?

Comments of the answer: the statistical significance added in the table 09 and discussed in the section 3.4.1.

P24 3.5.2 and Table 10 should explain why Sample C has the lowest TPC and the highest antioxidant activity, this is not consistent with the results in Table 09.

Comments of the answer: the value of the sample S_c was incorrectly imputed.

P25 F RAP value - correct to FRAP value.

Comments of the answer: the “F RAP value” corrected to “FRAP value”.

P25 The sentence "showed higher total phenolic compound compared to control" is misleading, the statistical differences were not found for all samples.

Comments of the answer: the sentence was corrected.

P26 3.5.4 and Table 12 do not describe FRAP, it should be stated why it is not used or added.

Comments of the answer: Due to the some difficulties in the laboratory the FRAP test are not include for the noodles sample.

P27 The fact that the water absorption index, water solubility index and swelling capacity are high does not mean that they are capable of forming a 3D network. The sentence "It indicates that these two powdered ingredients (seed powder and hull powder) can be replaced by wheat flour for making less gluten biscuits" should be more clearly described or deleted.

Comments of the answer: in the section 3.5, the sentence was changed and more literature and description was added.

P28 Tables 14 and 15: standardise the labelling of the samples, 6.00c- use the superscript.

Comments of the answer: the tables labeling and the superscript are corrected.

P29 Effect of mineral compounds on nutrition should be discussed with literature.

Comments of the answer: the tables labeling and the superscript are corrected.

P30 Conclusion, antioxidant activity should be mentioned, rheological properties were not instrumentally analysed so should not be mentioned in the conclusion.

Comments of the answer: The sentence of the conclusion describe briefly and make it simple. The antioxidant activity is mentioned in the section.

Reviewer #3:

Rewrite the abstract, introduction and conclusions.

Need to add photographs of the products and raw material used in the materials.

Comments of the answer:

Thanks you for your positive view about the manuscript. We rewrite the abstract, introduction and conclusion section according to your suggestion. We add more information and references and more literature in the introduction and discussion section.

The photo of the samples are added in “Supporting Information files - Photo”
