# Supplementary material for: Enhancing nutritional value and quality of cookies through pumpkin peel and seed powder fortification
Source: PLoS One. 2025 Feb 27;20(2):e0307506. doi: 10.1371/journal.pone.0307506 (PMC11867389; doi:10.1371/journal.pone.0307506)
Supplement: Attachment — Submitted filename: review plosONE.docx [file pone.0307506.s001.docx]

The article deals with a topical issue. The replacement of the proportion of wheat in bakery products is still topical and leads to the production of healthier and greener products. Pumpkin pulp and seeds are an impact product, where their further use is clearly related to the rational use of waste products in production plants.

I recommend the article for publication after incorporating the comments.

P11 2.1. the variety and degree of ripeness should be mentioned. The city of Dhaka should also be mentioned with the country. The wheat flour used should be characterised more precisely, gliadin, protein, starch content or technological property should be mentioned.

Figures 1-3 have different labelling of the samples, there are mixed samples1-4, formula1-4 and in the results are discus samples A-B. This should be standardised. This figure also shows the abbreviation gm, this should be explained in the note, or if it is g, it should be changed.

P13 Figure 2 the weight of the ingredients should be stated separately "Water & eggs 33g".

P15 2.4 and 25 why is it written "sweet potato peel"?

P16 For used equipment the manufacturer and country of origin should be stated. The source of the chemical used should also be mentioned.

P17 Table 1 shows no statistical differences between the samples, but the mean is different and the SD is low. The author should explain this.

P18 biscuits33.05/100g - missing space

P18 2nd column (C) and P19 3C - the author should explain how fat oxidation can change fat content or delete this sentence.

P18 "Dietary fibre values were significantly different between the cookies and the highest was found in sample D (1.61/100g), while the lowest content (0.32/100g) was investigated in sample A. This observation was comparable to that of Syam et al. (2019) who reported a fat value of 1.64/100g in pumpkin seed cookies [17]." - Correctly described ingredients

P21 Chapter 3.2 should be part of 3.1 as in previous biscuits.

P22 Table 05 sample labelling should be identical in manuscript,

P23 Table 06 use the same orientation in the cell, for Delta E there are no significant differences but the values are different and the SD is low, this should be explained.

P24 1C I did not agree that seeds are a better source of polyphenols. This should be confirmed by literature or discussed in more detail. In my opinion there are interferences with proteins in seeds.

P24 Table 09 were there no statistical differences?

P24 3.5.2 and Table 10 should explain why Sample C has the lowest TPC and the highest antioxidant activity, this is not consistent with the results in Table 09.

P25 F RAP value - correct to FRAP value.

P25 The sentence "showed higher total phenolic compound compared to control" is misleading, the statistical differences were not found for all samples.

P26 3.5.4 and Table 12 do not describe FRAP, it should be stated why it is not used or added.

P27 The fact that the water absorption index, water solubility index and swelling capacity are high does not mean that they are capable of forming a 3D network. The sentence "It indicates that these two powdered ingredients (seed powder and hull powder) can be replaced by wheat flour for making less gluten biscuits" should be more clearly described or deleted.

P28 Tables 14 and 15: standardise the labelling of the samples, 6.00c- use the superscript.

P29 Effect of mineral compounds on nutrition should be discussed with literature.

P30 Conclusion, antioxidant activity should be mentioned, rheological properties were not instrumentally analysed so should not be mentioned in the conclusion.
