# Supplementary material for: Enhancing nutritional value and quality of cookies through pumpkin peel and seed powder fortification
Source: PLoS One. 2025 Feb 27;20(2):e0307506. doi: 10.1371/journal.pone.0307506 (PMC11867389; doi:10.1371/journal.pone.0307506)
Supplement: Attachment — Submitted filename: Response to reviewers.docx [file pone.0307506.s003.docx]

**Detailed response to reviewers**

Manuscript Reference: PONE-D- 23-44103

Manuscript Title: Enhancing Nutritional Value and Quality of Cookies through Pumpkin Peel and Seed Powder Fortification.

Author(s): Md. Asaduzzaman^1,2^, Mahbubur Rahman^1^, Mst. Sorifa Akter^3*^

1) Department of Food Engineering and Technology, State University of Bangladesh, Dhaka, Bangladesh.
2) Department of Food Processing and Preservation, Hajee Mohammad Danesh Science and Technology University, Dinajpur, Bangladesh
3) Department of nutrition and food Engineering, Daffodil International University, Dhaka, Bangladesh.

*Corresponding Author

Dear Editor and Reviewers,

Thank you very much for your constructive and useful suggestions. Those comments were valuable and very helpful for revising and improving our paper, aswell as for increasing the significance of our research. The responses to editor andreviewer’s comments/suggestions of our manuscript have been answered carefullypoint-by-point as follows. Some minor mistakes within the manuscript have alsobeen corrected.

Note: All the line numbers in our responses refer to the revised manuscript. Thered color in the manuscript indicates the changes made during revision.

**Response to reviewers**

**Reviewer #1:**

Thank you for your positive perspective on the manuscript.

The comments of the reviewer

Title:
Is it really going to be “A Wheat Flour Replacement Approach”? It's tough to say with such a low amount of seeds and peel powder. Besides, pumpkin seeds are very small amounts, considering the massive amount of wheat flour produced worldwide. I suggest deleting this portion: “A Wheat Flour Replacement Approach.”

Comments of the answer:

According to the suggestion of the reviewer, “A Wheat Flour Replacement Approach” is deleting from the title.

Abstract:
-“Significant differences (p<0.05) in protein, fibre, beta……..”---This statement is not clear. For this sentence, we do not know if the amounts are higher or lower or if they have a positive or negative impact on product quality. Please make it clear.

As you mentioned % of peel, seed, etc., in the results and conclusion of the abstract, you should add this to the methodology, too.

You have also mentioned Overall acceptability in the results of the abstract but did not mention it in the methods of the abstract.

Comments of the answer:

The abstract is rewritten according to the suggestion of the reviewer 01. The methodology is added in the abstract.

Introduction:
-Use updated data. You used data from 2005, and now it’s 2024!

-The introduction section is very short; it lacks literature study and references. The novelty of the work has not been stated properly.

Comments of the answer:

The updated data added in the introduction section and data are updated. There more literature and references added in the introduction.

Material and Methods:

-Section 2.2: Were the seeds husked before milling?

Comments of the answer: we did not husked the seeds before milling, we add the husked to in the cookies and noodles development.

-Using both Figures 1 and 2 is unnecessary. You can merge them and mention the seed/peel portion in different samples. Otherwise, you may discard both of the figures and illustrate them in your writing.

Comments of the answer: While it is true that both figures share many commonalities, combining them would result in overly complicated design and complexity in understanding. Hence, we have chosen to maintain them as separate figures. However, should you suggest amalgamating them into one figure; we will provide detailed representation of the leveling process in the methodology section.

The samples in Figures 1 to 3 originally had disparate labeling, which has now been standardized uniformly under the same notation.

S_A_ = Control (100% market wheat flour, Cookie)

S_B_ = 95% market wheat flour /5%pumpkin seed cookie

S_C_ = 90% market wheat flour /10%pumpkin seed cookie

S_D_ = 85% market wheat flour /15%pumpkin seed cookie

S_E_ = 80%market wheat flour /20%pumpkin seed cookie

P_A_ = Control (100% market wheat flour, Cookie)

P_B_ = 95%wheat/5%pumpkin peel cookie

P_C_ = 90%wheat/10%pumpkin peel cookie

P_D_ = 80%wheat/20%pumpkin peel cookie

N_A_ = Control (100% wheat flour noodles)

N_B_ = 90%wheat/10% pumpkin seeds noodles

N_C_ = 90%wheat/10% pumpkin peels noodles

N_D_ = 80%wheat/10%pumpkin seeds and 10% pumpkin peels noodles

-Section 2.4 and 2.5: Where does sweet potato powder come here?

Comments of the answer: Mistakenly we wrote “sweet potato peel” which corrected to pumpkin seeds powder, peels powders, cookies and noodles in the section 2.4 and 2.5.

-Section 2.4: If the Protein determination process is through the Kjeidhal method, please mention the N conversion factor.

Comments of the answer: In the section 2.4 – the N conversion factor is added.

-Section 2.5-2.9: You must have to add all the details about the methodology. These methods are not reproducible and not suitable for publication.

Comments of the answer: according to your suggestion all section 2.5 to 2.8 are described. One section is added to the previous section, so that, one section is minimized.

-Section 2.10: The sensory analysis should be vividly described. Also, the number of panellists needs to be higher. Add whether they were trained/semi-trained, how samples were presented to them, etc. The statistical analysis portion should be moved to section 2.11.

Comments of the answer: The sensory analysis methodology has been updated in accordance with the reviewer's suggestions. The statistical analysis section has been relocated to Section 2.10. All panelists are faculty members of the Department of Food Engineering and Technology, and prior to the sensory analysis, they underwent training for the tests. I regret the limited number of panelists; however, currently, our department lacks qualified sensory panelists. We are actively working to increase the number of panelists and are organizing training sessions to recruit and train new members.

-Section 2.11: Mention how many replicates for each observation were used.

Comments of the answer: All evaluations of samples were all done in triplicate.

Results and Discussion:

-Table 01: Check 3.9.1±0.48b?

Comments of the answer: the data was corrected. It will be 3.91±0.48^b^

-In methodology, you mentioned Sample 1, 2, 3, etc…in Results and Discussion, you are saying Sample A, B, C, etc…

Comments of the answer: The sample indications were wrong which was corrected and described according to the data in the methodology section.

-If every result of your study is comparable to those of Syam et al. (2019), then what is the justification of your study?

Comments of the answer: Along with syam et al. (2019), more literature added in the section 3.1.2. such as Alshehry (2020) and Apostol et al. (2020)

-Where is the Carbohydrate content in your results???

Comments of the answer: There are no carbohydrate discussions in this study, so the carbohydrate portion was removed from methodology.

-Section 3.2: “The antioxidant capacities of leaves????????? may vary………..”? You should be careful while writing a manuscript for publication.

Comments of the answer: thank you the suggestion, we will be more careful to write the manuscript for publishing in this renowned journal.

-Section 3.3: Add the units of cooking loss and cooking yield. From the standard deviation, it seems the indications of significant differences are not correct.

Comments of the answer: We add more literature in this section to differentiate differences.

-Table 12: The results of sample D are not homogenous with the other results. Besides, where are the FRAP results of Noodles?

Comments of the answer: The description is corrected and due to the some difficulties in the laboratory the FRAP test are not include for the noodles sample.

-Section 3.6: You should mention the significance of Hydration properties. The discussion of this section is also inadequate. Like other parameters, you didn’t measure the hydration properties of your samples.

Comments of the answer: in this section firstly we discussed the importance of the hydration properties and then we add more discussion and literatures.

-Section 3.7: Previously, you mentioned 10 panellists!

Comments of the answer: Mistakenly we count our self as a panelist which was corrected.

-Table 14, 15, 16: You need to add the standard deviations in the results.

-Besides, this section lacks discussion.

Comments of the answer: The standard deviation is added in table 14 to 16. Description and literature is also added.

-Minerals content: You did not mention this in the methodology. If you would like to keep it, you should add it before sensory evaluation. Obviously, you should add mineral contents to all samples and ingredients as well. You should improve the discussion section also.

Comments of the answer: The section on mineral content has been removed because no other confectionery items besides noodles and samples were examined for minerals, which is not relevant to this study.

Conclusions:
-The first sentence is not clear. Make it simple.

Comments of the answer: The conclusion sentence has been simplified for brevity. The antioxidant activity is discussed in the corresponding section.

References:
Most of the references used in the manuscripts are too old. You should use more updated references.

Comments of the answer: In the references section, updated refinances are added which cited in the study.

**Reviewer #2:**

P11 2.1.the variety and degree of ripeness should be mentioned. The city of Dhaka should also be mentioned with the country. The wheat flour used should be characterised more precisely, gliadin, protein, starch content or technological property should be mentioned.

Comments of the answer:

In Section 2.1, in compliance with the reviewer's suggestion, Dhaka is now accompanied by the country name in the author's affiliation and the materials and methods section. Additionally, the gluten value was included in the manuscript following the powder test as per the reviewer's recommendation. Furthermore, the scientific name of the pumpkin variety and the degree of ripeness are detailed in the materials and methods section.

Figures 1-3 have different labelling of the samples, there are mixed samples1-4, formula1-4 and in the results are discus samples A-B. This should be standardised. This figure also shows the abbreviation gm, this should be explained in the note, or if it is g, it should be changed.

Comments of the answer:

Figure 1 to 3 had different labeling of the samples which now converted in to same notation and standardised uniformly where

S_A_ = Control (100% market wheat flour, Cookie)

S_B_ = 95% market wheat flour /5%pumpkin seed cookie

S_C_ = 90% market wheat flour /10%pumpkin seed cookie

S_D_ = 85% market wheat flour /15%pumpkin seed cookie

S_E_ = 80%market wheat flour /20%pumpkin seed cookie

P_A_ = Control (100% market wheat flour, Cookie)

P_B_ = 95%wheat/5%pumpkin peel cookie

P_C_ = 90%wheat/10%pumpkin peel cookie

P_D_ = 80%wheat/20%pumpkin peel cookie

N_A_ = Control (100% wheat flour noodles)

N_B_ = 90%wheat/10% pumpkin seeds noodles

N_C_ = 90%wheat/10% pumpkin peels noodles

N_D_ = 80%wheat/10%pumpkin seeds and 10% pumpkin peels noodles

P13 Figure 2 the weight of the ingredients should be stated separately "Water & eggs 33g".

Comments of the answer: In the figure 02, the weight of the ingredients mistakenly wrote water and egg 33g. It should be only egg 33 g which is corrected in the figure.

P15 2.4 and 25 why is it written "sweet potato peel"?

Comments of the answer: Mistakenly we wrote “sweet potato peel” which corrected to pumpkin seeds powder, peels powders, cookies and noodles in the section 2.4 and 2.5.

P16 For used equipment the manufacturer and country of origin should be stated. The source of the chemical used should also be mentioned.

Comments of the answer: The equipment manufacturer and origin are added in the 2.2 section including country. The source of the chemical used in the study also included in the section 2.1.

P17 Table 1 shows no statistical differences between the samples, but the mean is different and the SD is low. The author should explain this.

Comments of the answer: The statistical differences between the samples are corrected and the explanation is added in the section 3.1.1.

P18 biscuits33.05/100g - missing space

Comments of the answer: the space is added between biscuits 33.05/100g in the section 3.1.2.

P18 2nd column (C) and P19 3C - the author should explain how fat oxidation can change fat content or delete this sentence.

Comments of the answer: The explanation of fat oxidation is added in the section 3.1.1. This also repeated in the section 3.1.2.

P18 "Dietary fibre values were significantly different between the cookies and the highest was found in sample D (1.61/100g), while the lowest content (0.32/100g) was investigated in sample A. This observation was comparable to that of Syam et al. (2019) who reported a fat value of 1.64/100g in pumpkin seed cookies [17]." - Correctly described ingredients

Comments of the answer: The sample indications were wrong which was corrected and described according to the data.

P21 Chapter 3.2 should be part of 3.1 as in previous biscuits.

Comments of the answer: chapter 3.2 is changed to 3.1.5

P22 Table 05 sample labelling should be identical in manuscript,

Comments of the answer: Table 05 sample labeling is corrected and wrote according to the formulation figure and data.

P23 Table 06 use the same orientation in the cell, for Delta E there are no significant differences but the values are different and the SD is low, this should be explained.

Comments of the answer: in the table 06, the leveling data is corrected and the explanations are added in the section 3.3.4. More literature is added describe in the section.

P24 1C I did not agree that seeds are a better source of polyphenols. This should be confirmed by literature or discussed in more detail. In my opinion there are interferences with proteins in seeds.

Comments of the answer: the pumpkin peels are more better source of the total phenolic compounds then pumpkin seeds which was discussed in the section 3.4.1

P24 Table 09 were there no statistical differences?

Comments of the answer: the statistical significance added in the table 09 and discussed in the section 3.4.1.

P24 3.5.2 and Table 10 should explain why Sample C has the lowest TPC and the highest antioxidant activity, this is not consistent with the results in Table 09.

Comments of the answer: the value of the sample S_c_ was incorrectly imputed.

P25 F RAP value - correct to FRAP value.

Comments of the answer: the “F RAP value” corrected to “FRAP value”.

P25 The sentence "showed higher total phenolic compound compared to control" is misleading, the statistical differences were not found for all samples.

Comments of the answer: the sentence was corrected.

P26 3.5.4 and Table 12 do not describe FRAP, it should be stated why it is not used or added.

Comments of the answer: Due to the some difficulties in the laboratory the FRAP test are not include for the noodles sample.

P27 The fact that the water absorption index, water solubility index and swelling capacity are high does not mean that they are capable of forming a 3D network. The sentence "It indicates that these two powdered ingredients (seed powder and hull powder) can be replaced by wheat flour for making less gluten biscuits" should be more clearly described or deleted.

Comments of the answer: in the section 3.5, the sentence was changed and more literature and description was added.

P28 Tables 14 and 15: standardise the labelling of the samples, 6.00c- use the superscript.

Comments of the answer: the tables labeling and the superscript are corrected.

P29 Effect of mineral compounds on nutrition should be discussed with literature.

Comments of the answer: the tables labeling and the superscript are corrected.

P30 Conclusion, antioxidant activity should be mentioned, rheological properties were not instrumentally analysed so should not be mentioned in the conclusion.

Comments of the answer: The sentence of the conclusion describe briefly and make it simple. The antioxidant activity is mentioned in the section.

**Reviewer #3:**

Rewrite the abstract, introduction and conclusions.

Need to add photographs of the products and raw material used in the materials.

Comments of the answer:

Thanks you for your positive view about the manuscript. We rewrite the abstract, introduction and conclusion section according to your suggestion. We add more information and references and more literature in the introduction and discussion section.

The photo of the samples are added in “Supporting Information files - Photo”
