# Supplementary material for: Enhancing nutritional value and quality of cookies through pumpkin peel and seed powder fortification
Source: PLoS One. 2025 Feb 27;20(2):e0307506. doi: 10.1371/journal.pone.0307506 (PMC11867389; doi:10.1371/journal.pone.0307506)
Supplement: Attachment — Submitted filename: Response to Academic Editor & reviewers.docx [file pone.0307506.s005.docx]

**Detailed response to Academic Editor & reviewers**

Manuscript Reference: PONE-D-23-44103_R1

Manuscript Title: Enhancing Nutritional Value and Quality of Cookies through Pumpkin Peel and Seed Powder Fortification.

Author(s): Md. Asaduzzaman^1,2^, Mahbubur Rahman^1^, Mst. Sorifa Akter^3*^

1) Department of Food Engineering and Technology, State University of Bangladesh, Dhaka, Bangladesh.
2) Department of Food Processing and Preservation, Hajee Mohammad Danesh Science and Technology University, Dinajpur, Bangladesh
3) Department of nutrition and food Engineering, Daffodil International University, Dhaka, Bangladesh.

*Corresponding Author

Dear Editor and Reviewers,

Thank you very much for your constructive and useful suggestions. Those comments were valuable and very helpful for revising and improving our paper, as well as for increasing the significance of our research. The responses to editor and reviewer’s comments/suggestions of our manuscript have been answered carefully point-by-point as follows. Some minor mistakes within the manuscript have also been corrected.

Note: All the line numbers in our responses refer to the revised manuscript. The red color in the manuscript indicates the changes made during revision.

**RESPONSE TO EDITOR**

01. **Comments:**

The manuscript titled “Enhancing Nutritional Value and Quality of Cookies through Pumpkin Peel and Seed Powder Fortification” presented by Md. Asaduzzaman discussed the novel approach to incorporate pumpkin seed powder as raw material for cookies and noodle and to provide a sustainable way of producing the food. Overall manuscript written well but I feel the manuscript has serious flows in presentation and formatting.

**Answer of the Comments:** Thank you for your positive perspective on the manuscript.

02. **Comments:**

Author should carefully check the manuscript.

Reference- Author has marked reference with both numbered and author et al., . Author should read the Journal instruction for reference. There are many small paragraph that needs to be merged along with content for understanding of the readers. Discussion stills needs improvement.
**Answer of the Comments:** All reference is corrected including marked in the introduction section and written according to the journal instruction. According to the suggestion the small paragraphs are merged. We try our best to improve the discussion section by adding more references and secondary data.

03. **Comments:**

A detailed reviewer report mark in pdf file, kindly refer to pdf page.

**Answer of the Comments:** The suggestions are corrected according to reviewer and in needed next time, all answers and remarks will be written on the basis of PDF page.

04. **General comments:**

At several places there is spacing issue, authors are suggested to correct it

**Answer of the Comments:** The spacing issues are corrected including keywords which were happened due to the version change of the Microsoft word.

05. Comments: Minor English and grammar mistakes are present that needs to be thoroughly checked to improve the quality of the manuscript. Additionally, there are few sentences that are too large for the readers with many comma ( , ), that needs author attention.

**Answer of the Comments:**  we do our best improve the language of the manuscript. Some sentence are broke down and simplify, so that it will more easy to understand.

06. **Comments:**

**Page 17 section 1:** Remove underline & merge paragraph one to three.

**Answer of the Comments:** The underline removed and the paragraphs are merged.

07. **Comments:**

**Page 19, section 2.1:** Italic

**Answer of the Comments:** The scientific name of pumpkin changed into italic format.

08. **Comments:**

**Page 19, section 2.2:** Author recommended to put reference.

**Answer of the Comments:** References are added.

09. **Comments:**

**Page 19, section 3.3:** Check for word size.

**Answer of the Comments:** The word sizes are corrected.

10. **Comments:**

**Page 19, section 3.4.3:** why bold

**Answer of the Comments:** The word format is corrected.

**RESPONSE TO REVIEWERS**

**Reviewer:**

Thank you for your positive perspective on the manuscript.

**The comments of the reviewer:**

 All recommendations have been accepted. I have recommended the article for publication. Only one comment: in chapter 3.4.3, first column, m mole/100g is in bold and should be in normal font.

Comments of the answer:

According to the suggestion of the reviewer, the text was corrected.
