# Supplementary material for: Enhancing nutritional value and quality of cookies through pumpkin peel and seed powder fortification
Source: PLoS One. 2025 Feb 27;20(2):e0307506. doi: 10.1371/journal.pone.0307506 (PMC11867389; doi:10.1371/journal.pone.0307506)
Supplement: Attachment — Submitted filename: Rev comments_PONE-D-23-44103_R2.docx [file pone.0307506.s006.docx]

**Review Report** for the Manuscript PONE-D-23-44103R2 entitled:

“Enhancing Nutritional Value and Quality of Cookies through Pumpkin Peel and Seed Powder Fortification”

This manuscript makes a valuable contribution to the field of circular economy, describing a complex research that provides new insights into the possibility of exploiting the nutritional and bioactive composition of pumpkin by-products, such as peels and seeds, in the confectionery sector.

The study extends the knowledge of incorporating different pumpkin by-products (peels and seeds) in different concentrations to develop value-added cookie and noodle formulas.

The introduction is coherent and adequately structured, the literature review has been critically followed in line with the current state of knowledge. The aim of the research is clearly stated, the objectives are realistic, well defined and achievable.

The study is well planned and the methods are well suited to achieve the proposed objectives. The research methodology is generally well detailed, but needs some improvement, in particular in terms of design and presentation of flowcharts, as follows:

The cookie recipe, including ingredient quantities, could be put into one table for all cookies formulated with pumpkin seeds and pumpkin seeds, and a single cookie-making flowchart could be included because the process is identical. Quantities of materials do not have to be listed in the chart.

The same for noodles, a table with the recipe and a flowchart for their preparation.

The labelling of the biscuit and noodle samples respectively will be included in the tables in which the recipes were presented, so the list of samples will no longer be presented separately.

The part below will be removed:

SA = PA = Control (100% market wheat flour, Cookie)

SB = 95% market wheat flour /5%pumpkin seed cookie

SC = 90% market wheat flour /10%pumpkin seed cookie

SD = 85% market wheat flour /15%pumpkin seed cookie

SE = 80%market wheat flour /20%pumpkin seed cookie

PB = 95%wheat/5%pumpkin peel cookie

PC = 90%wheat/10%pumpkin peel cookie

PD = 80%wheat/20%pumpkin peel cookie

The list of the noodles samples are

NA = Control (100% wheat flour noodles)

NB = 90%wheat/10% pumpkin seeds noodles

NC = 90%wheat/10% pumpkin peels noodles

ND = 80%wheat/10%pumpkin seeds and 10% pumpkin peels noodles

Please pay more attention to the wording, e.g. “There were two types of cookies were formulated”, which could better be “Two types of cakes have been formulated.”

The results are clearly presented, but the number of 16 tables is excessive.

Tables 2, 3 and 4 could be merged (Proximate compositions of formulated cookies and noodles ), also Tables 7, 8 and 9 *(*Hunter Color Values (L*, a*,b*,ΔE) of formulated cookies and noodles), Tables 10, 11 and 12 (Total phenolic compound and antioxidant properties (DPPH and FRAP) of formulated cookies and noodles), Tables 15, 16 and 17 (Mean score for texture, aroma, color, taste and overall acceptability of formulated cookies and noodles).

The results obtained are discussed in detail and relevant explanations are provided, with the findings resulting from this research being well highlighted. This is a wide-ranging paper, which has generated many useful results of considerable practical value.

Some improvements are needed in the discussion to better highlight the added value of the research. Also, the RESULTS AND DISCUSSION section will be rearranged and renumbered accordingly.

The conclusions support the results of this study. This section needs to be improved, showing the genuine value and innovation of this study. What are the limitations of including pumpkin peel and seed powder in cookies and noodles?

The references are relevant to this research topic.

The study has high applicative potential, is a high quality and high impact approach in the field, therefore I recommend its publication after review.
