# Supplementary material for: Enhancing nutritional value and quality of cookies through pumpkin peel and seed powder fortification
Source: PLoS One. 2025 Feb 27;20(2):e0307506. doi: 10.1371/journal.pone.0307506 (PMC11867389; doi:10.1371/journal.pone.0307506)
Supplement: Attachment — Submitted filename: Response to Academic Editor & reviewers.docx [file pone.0307506.s007.docx]

**Detailed response to Academic Editor & reviewers**

Manuscript Reference: PONE-D-23-44103_R2

Manuscript Title: Enhancing Nutritional Value and Quality of Cookies through Pumpkin Peel and Seed Powder Fortification.

Author(s): Md. Asaduzzaman^1,2^, Mahbubur Rahman^1^, Mst. Sorifa Akter^3*^

1) Department of Food Engineering and Technology, State University of Bangladesh, Dhaka, Bangladesh.
2) Department of Food Processing and Preservation, Hajee Mohammad Danesh Science and Technology University, Dinajpur, Bangladesh
3) Department of nutrition and food Engineering, Daffodil International University, Dhaka, Bangladesh.

*Corresponding Author

Dear Editor and Reviewers,

Thank you very much for your constructive and useful suggestions. Those comments were valuable and very helpful for revising and improving our paper, as well as for increasing the significance of our research. The responses to editor and reviewer’s comments/suggestions of our manuscript have been answered carefully point-by-point as follows. Some minor mistakes within the manuscript have also been corrected.

Note: All the line numbers in our responses refer to the revised manuscript. The red color in the manuscript indicates the changes made during revision.

**RESPONSE TO EDITOR**

01. **Comments:**

Please review your reference list to ensure that it is complete and correct. If you have cited papers that have been retracted, please include the rationale for doing so in the manuscript text, or remove these references and replace them with relevant current references. Any changes to the reference list should be mentioned in the rebuttal letter that accompanies your revised manuscript. If you need to cite a retracted article, indicate the article’s retracted status in the References list and also include a citation and full reference for the retraction notice.

**Answer of the Comments:** We are extremely sorry; we cannot find any reference that should be retracted. We have reviewed all references, but none warrant retraction.

**RESPONSE TO REVIEWERS**

**Reviewer:**

Thank you for your positive perspective on the manuscript. We are pleased to hear that the introduction, methodology & references met your expectations. We will try to follow your suggestions to improve the study.

01) **The comments of the reviewer:**

The cookie recipe, including ingredient quantities, could be put into one table for all cookies formulated with pumpkin seeds and pumpkin seeds, and a single cookie-making flowchart could be included because the process is identical. Quantities of materials do not have to be listed in the chart.

The same for noodles, a table with the recipe and a flowchart for their preparation.

The labelling of the biscuit and noodle samples respectively will be included in the tables in which the recipes were presented, so the list of samples will no longer be presented separately.

**Answer of the Comments:** Following the reviewer's suggestion, we have included two tables in the study where all ingredient quantities are listed. The labeling has been added to the table, and the quantities have been removed from the chart. A flowchart has been prepared to represent all the cookies. Thank you for the excellent recommendation.

02) **The comments of the reviewer:**

The part below will be removed:

SA = PA = Control (100% market wheat flour, Cookie)

SB = 95% market wheat flour /5%pumpkin seed cookie

SC = 90% market wheat flour /10%pumpkin seed cookie

SD = 85% market wheat flour /15%pumpkin seed cookie

SE = 80%market wheat flour /20%pumpkin seed cookie

PB = 95%wheat/5%pumpkin peel cookie

PC = 90%wheat/10%pumpkin peel cookie

PD = 80%wheat/20%pumpkin peel cookie

The list of the noodles samples are

NA = Control (100% wheat flour noodles)

NB = 90%wheat/10% pumpkin seeds noodles

NC = 90%wheat/10% pumpkin peels noodles

ND = 80%wheat/10%pumpkin seeds and 10% pumpkin peels noodles

**Answer of the Comments:** According to the suggestion of the reviewer, this is removed.

03) **The comments of the reviewer:**

Please pay more attention to the wording, e.g. “There were two types of cookies were formulated”, which could better be “Two types of cakes have been formulated.”

**Answer of the Comments:** According to the suggestion of the reviewer, this is corrected.

04) **The comments of the reviewer:**

The results are clearly presented, but the number of 16 tables is excessive.
Tables 2, 3 and 4 could be merged (Proximate compositions of formulated cookies and noodles ), also Tables 7, 8 and 9 (Hunter Color Values (L*, a*,b*,ΔE) of formulated cookies and noodles), Tables 10, 11 and 12 (Total phenolic compound and antioxidant properties (DPPH and FRAP) of formulated cookies and noodles), Tables 15, 16 and 17 (Mean score for texture, aroma, color, taste and overall acceptability of formulated cookies and noodles).

**Answer of the Comments:** Following the reviewer's suggestion, Tables 2, 3, and 4 can be merged, as well as Tables 7, 8, and 9; Tables 10, 11, and 12; and Tables 15, 16, and 17. However, the statistical analysis differs for each table, and merging these tables would complicate the understanding of the sample differences. Nonetheless, if the reviewer recommends merging the tables, we will proceed accordingly.

05) **The comments of the reviewer:**

The results obtained are discussed in detail and relevant explanations are provided, with the findings resulting from this research being well highlighted. This is a wide-ranging paper, which has generated many useful results of considerable practical value.

Some improvements are needed in the discussion to better highlight the added value of the research. Also, the RESULTS AND DISCUSSION section will be rearranged and renumbered accordingly.

**Answer of the Comments:**

We are pleased to hear that the result and discussion section met your expectations. All tables are rearranged and renumbered accordingly.

06) **The comments of the reviewer:**

The conclusions support the results of this study. This section needs to be improved, showing the genuine value and innovation of this study. What are the limitations of including pumpkin peel and seed powder in cookies and noodles?

**Answer of the Comments:** Thank you for your comments. This section now includes some important values as well as the limitations.
